# Downregulation of mTOR Signaling Increases Stem Cell Population Telomere Length during Starvation of Immortal Planarians

**DOI:** 10.1016/j.stemcr.2019.06.005

**Published:** 2019-07-25

**Authors:** Marta Iglesias, Daniel A. Felix, Óscar Gutiérrez-Gutiérrez, Maria del Mar De Miguel-Bonet, Sounak Sahu, Beatriz Fernández-Varas, Rosario Perona, A. Aziz Aboobaker, Ignacio Flores, Cristina González-Estévez

**Affiliations:** 1 Leibniz Institute on Aging-Fritz Lipmann Institute (FLI), Beutenbergstrasse 11, 07745 Jena, Germany; 2 Centro Nacional de Investigaciones Cardiovasculares Carlos III (CNIC), Melchor Fernández Almagro 3, 28029 Madrid, Spain; 3 Department of Zoology, University of Oxford, South Parks Road, Oxford OX1 3PS, UK; 4 Instituto de Investigaciones Biomédicas CSIC/UAM, IDiPaz, Arturo Duperier 4, 28029 Madrid, Spain; 5 Ciber Network on Rare Diseases (CIBERER), C/ Alvaro de Bazan, 10, 46010 Valencia, Spain

**Keywords:** planarian, mTOR, starvation, stem cell, telomere, aging, neoblast, fasting, immortal, SMG-1

## Abstract

Reduction of caloric intake delays and prevents age-associated diseases and extends the life span in many organisms. It may be that these benefits are due to positive effects of caloric restriction on stem cell function. We use the planarian model *Schmidtea mediterranea*, an immortal animal that adapts to long periods of starvation by shrinking in size, to investigate the effects of starvation on telomere length. We show that the longest telomeres are a general signature of planarian adult stem cells. We also observe that starvation leads to an enrichment of stem cells with the longest telomeres and that this enrichment is dependent on mTOR signaling. We propose that one important effect of starvation for the rejuvenation of the adult stem cell pool is through increasing the median telomere length in somatic stem cells. Such a mechanism has broad implications for how dietary effects on aging are mediated at the whole-organism level.

## Highlights

• Telomere quantitative FISH assay for planarian tissue sections and FACS-sorted cells
• Stem cells show higher median telomere length than their progeny
• Starvation leads to an enrichment of stem cells with the longest telomeres
• mTOR upregulation abolishes the effect of starvation on stem cells telomere length

## Graphical Abstract

**Figure:**
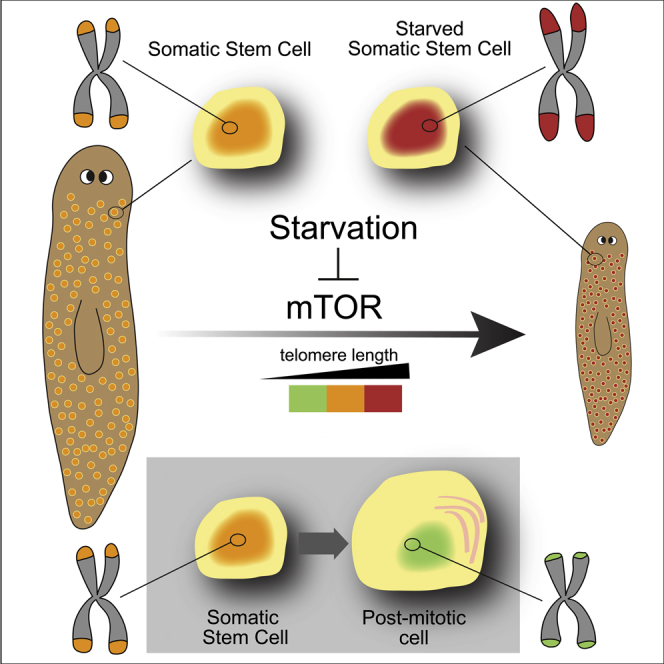

## Teaser

González-Estévez and colleagues show that long telomeres are a signature of planarian stem cells. They find that in the immortal planarian, starvation leads to an enrichment of stem cells with the longest telomeres dependent on mTOR signaling. They propose that starvation contributes to the rejuvenation of the adult stem cell pool by increasing their telomere length.

## Introduction

Calorie-restricted diets or periods of fasting (referred to as starvation in prokaryotes and invertebrates) extend the life span of many organisms and can also protect against age-related diseases in rodents, monkeys, and humans (Longo and Mattson, 2014). While there is a general consensus that diet is an important modulator of organismal physiology, molecular and physiological mechanisms through which this is mediated remain poorly described.

Many of our tissues are able to grow, respond to injury, and regenerate by relying on tissue-specific populations of adult stem cells. The regulation of self-renewal and differentiation of adult stem cells is therefore important for the function of many organs. It is now known that stem cell functionality decreases during aging contributing to age-associated pathologies and the overall aging process of the organism (Behrens et al., 2014). Diet is also an emerging important regulator of adult stem cell function. For instance, it has been reported that dietary restriction (DR) enhances stem cell functionality in muscle (Cerletti et al., 2012) and intestinal epithelium (Yilmaz et al., 2012) and improves repopulation capacity of hematopoietic stem cells in early mouse aging (Tang et al., 2016). Fasting, for example, increases the number of a subpopulation of crypt cells more primed to respond to repopulation of the intestine upon refeeding, protects mouse intestinal stem cells from lethal doses of chemotherapy (Richmond et al., 2015, Tinkum et al., 2015), and protects germline stem cells and extends reproductive longevity in *Caenorhabditis elegans* (Angelo and Van Gilst, 2009). While these examples demonstrate the effect of diet on stem cells, they do not provide much explanation of the potential mechanisms by which these effects are mediated.

Planarians are known for their astonishing power of full-body regeneration. The source of that power is the large population of adult stem cells or neoblasts in their bodies, and the planarian species *Schmidtea mediterranea* has become a consolidated model for the study of stem cells and regeneration (Aboobaker, 2011, Rink, 2013). The classical marker to label most proliferating planarian adult stem cells is a member of the Argonaute/PIWI protein family, *smedwi-1* (Reddien et al., 2005). We know that the *smedwi-1*-positive population is highly heterogeneous (van Wolfswinkel et al., 2014), with different degrees of potency and lineage commitment (Scimone et al., 2014). We also know that a fraction of *smedwi-1*^+^ cells are pluripotent (cNeoblasts) (Wagner et al., 2011). However, we still do not know how many pluripotent stem cells (PSCs) there are and where they would be located in the planarian body. It also remains unclear which, if any, specific molecular signatures are present in pluripotent planarian stem cells, including epigenetic signatures (Mihaylova et al., 2018) or changes in genome structural features such as telomere length.

Planarians are able to undergo prolonged starvation and shrink in size (or “degrow”); however, they maintain activity levels and normal physiology, morphological scale, and the same regenerative power as fed (or growing) animals. Growth and degrowth are repeating cycles in the planarian life history since food availability fluctuates in nature (Felix et al., 2019, Pellettieri, 2019). Planarians maintain the adult stem cell population throughout the process of starvation, as cycling cells remain at 25%–30% of the total cell population (Baguñà, 1976, Gonzalez-Estevez et al., 2012a). Recent data suggest that during starvation either the balance between asymmetric and symmetric divisions is skewed toward symmetric divisions, or there is a relative increase in the self-renewing population of stem cells in detriment of differentiation (Gonzalez-Estevez et al., 2012a, Mangel et al., 2016), in a similar way to what is observed in the mammalian intestine (Yilmaz et al., 2012). This stem cell maintenance strategy allows for a rapid response to a more favorable nutritional environment, or a regenerative response to injury (Felix et al., 2019). At the organismal level planarians do not seem to age, as the stem cell pool seems to be able to infinitely replace damaged or old cells and to respond to injury. It is currently unknown how starvation regulates planarian stem cell biology.

One of the hallmarks of aging is telomere attrition (Lopez-Otin et al., 2013). Telomeres are structures that protect chromosomes from DNA degradation and DNA repair mechanisms. Telomerase maintains telomere length and prevents the end-replication problem in those highly proliferative cells where it is expressed, usually germ stem cells and adult somatic stem cells. However the somatic levels of telomerase are usually not enough to prevent aging-related telomere shortening (Flores and Blasco, 2010). Telomere length has been linked to stem cell pluripotency. Both activation of telomerase and telomere length are known to positively correlate with embryonic stem cell/induced PSC (iPSC) pluripotency, and are also required for somatic cell reprogramming to generate functional iPSCs (Huang et al., 2011, Pucci et al., 2013, Schneider et al., 2013). Telomere quantitative fluorescence *in situ* hybridization (TelQ-FISH or telomapping) applied to diverse mice and plant tissues known to contain stem cell niches has generated topographic telomere length maps showing gradients of telomere length, with the longest telomeres marking the adult stem compartment and the shortest telomeres in the more differentiated compartments within a given tissue (Aida et al., 2008, Flores et al., 2008, Garcia-Lavandeira et al., 2009, Gonzalez-Garcia et al., 2015).

In this work we perform telomere length quantification to identify different populations of cells according to telomere length and the population of stem cells with the longest telomeres (which could potentially be the PSCs) and to check whether or not starvation has a positive effect on the telomere length of stem cells. We adapted the TelQ-FISH technology to *S*. *mediterranea* paraffin tissue sections and cells sorted by FACS (fluorescence-activated cell sorting). By measuring telomere length in cells of the planarian body *in situ* we observed that planarian adult stem cells have longer telomeres than their differentiated progeny. We also find that the *smedwi-1*^+^ stem cell population is highly heterogeneous for telomere length, correlating with their known heterogeneity with regard to potency and lineage commitment. We uncover a subclass of *smedwi-1*^+^ cells with the longest telomeres that correspond to the presumptive planarian germ stem cells and co-express the germline marker *Smed-nanos*. During starvation we observe that the pool of stem cells with the longest telomeres is highly enriched and that this enrichment is dependent on inhibition of mTOR (mammalian target of rapamycin) signaling. We show that fasting is able to rejuvenate the stem cell pool in terms of telomere length through downregulation of mTOR signaling. Our data contribute to the understanding of stem cell biology and aging, and highlight the importance of conducting aging research in long-lived animals.

## Results

### Planarian Stem Cells Display Higher Median Telomere Length Than Their Descendants

**Figure 1:**
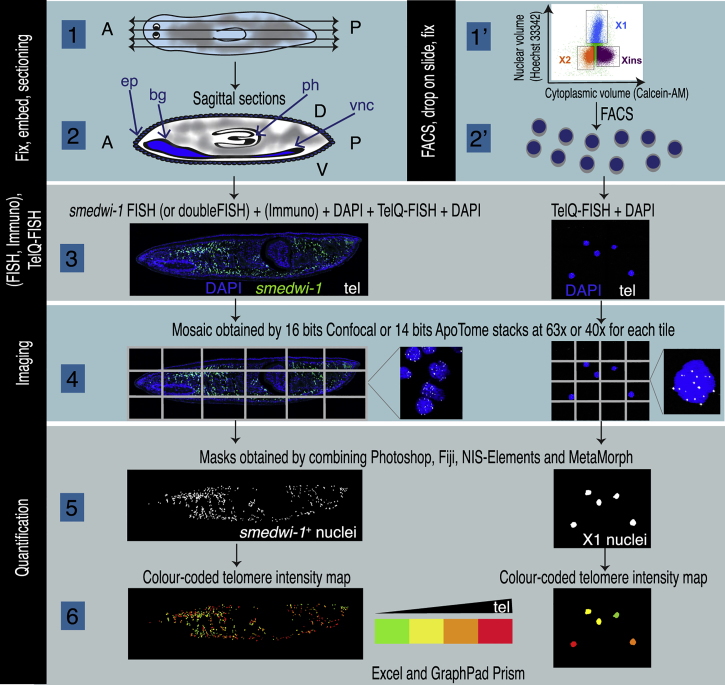
Experimental Flow Diagram (1 and 2) Fixation, embedding, and sectioning of planarians. Schematic 1 shows the sagittal sectioning planes of paraffin embedded planarians represented by double arrows. The distribution of stem cells in the planarian body is indicated in gray. Schematic 2 represents a sagittal section of the planarian. A, anterior; bg, brain ganglia; D, dorsal; ep, epidermis; ph, pharynx; P, posterior; V, ventral; vnc, ventral nerve cord. (1′ and 2′) Alternatively, planarian cells can be FACS sorted, dried, and fixed on a slide. (3) The next step consists of either single or double FISH followed or not by immunohistochemistry, DAPI staining, and TelQ-FISH or just TelQ-FISH and DAPI staining in case of FACS-sorted cells. The images represent a tissue section after FISH for *smedwi-1* (stem cells in green), TelQ-FISH (telomeres in gray), and DAPI staining (nuclei in blue) and some FACS-sorted cells. (4) Generation of high-resolution images of the telomeres as seen at high magnifications. (5 and 6) Quantification of telomere intensity is done by combining several imaging software packages. Raw data are then exported for further analysis. See also Figure S1.

We adapted TelQ-FISH or telomapping (Flores et al., 2008, Gonzalez-Suarez et al., 2000, Zijlmans et al., 1997) to asexual *S*. *mediterranea* paraffin tissue sections and fixed FACS-sorted cells (Figure 1 and Video S1; Experimental Procedures and Supplemental Experimental Procedures). Using TelQ-FISH we were able to detect an average of ∼15 telomeres of the 16 telomeres in FACS-sorted cells and ∼14 telomeres per cell in tissue sections (Figures S1A–S1C). A higher number than 16 is expected in planarian cells duplicating their DNA (and therefore their telomeres). A lower number than 16 is expected if two or more telomeres cluster together forming telomere foci, a common feature in other species (Molenaar et al., 2003). As previously reported for other organisms (Gilson and Londono-Vallejo, 2007), we also observed length variation of the telomeres within a cell (Figure S1D). As a way to further validate TelQ-FISH in planarians, we performed fluorescent *in situ* hybridization (FISH) for *smedwi-1* as a stem cell marker (Reddien et al., 2005) in paraffin tissue sections after *Smed-tert* RNAi (telomerase reverse transcriptase [TERT] planarian homolog). We observed a decrease in stem cell telomere length of approximately 3% in 5 weeks after *tert* downregulation (Figures S1E–S1G), which is comparable with the reported overall erosion rate of 1% decrease per week of treatment (290 bp per week) (Tan et al., 2012). However, Tan and colleagues saw a steep initial decline of telomere length, which we did not observe; this may be due to the fact that we analyzed only stem cells while they analyzed whole-planarian genomic DNA, and we used different methods to measure telomere length and possibly had different rates of stress during the RNAi experiment.

Video S1. Tissue Section Stained for Telomeres The video shows a zoom into a tissue section stained for telomeres with increased exposure in order to be able to observe all the telomeres from all the cells in all the planarian tissues.

**Figure 2:**
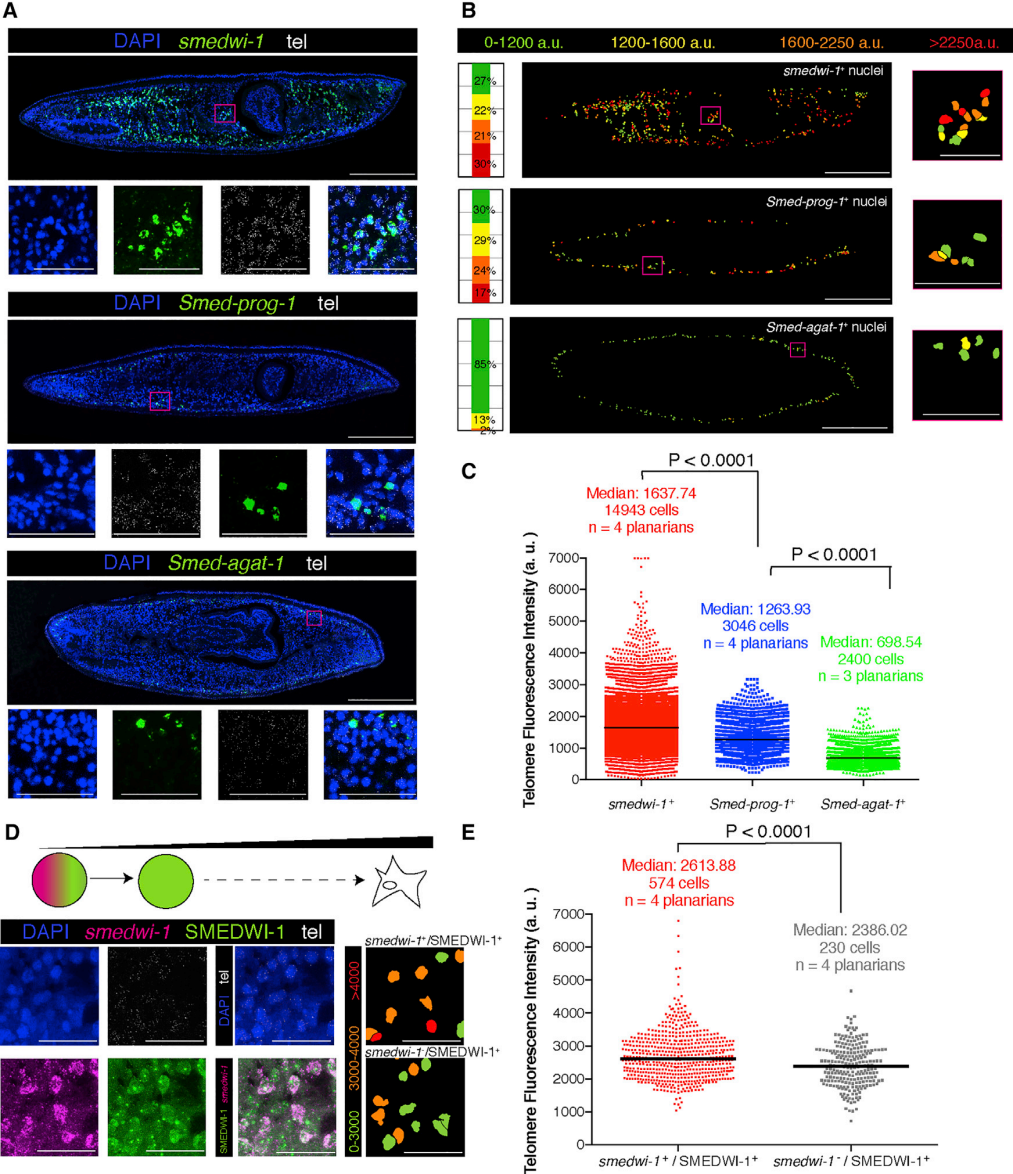
Planarian Stem Cells Display Higher Median Telomere Fluorescence Intensity than Immediate, Early, and Late Post-mitotic Descendant Cells (A) Maximum confocal projections of representative planarian paraffin sections. Magenta box indicates the region shown below. Anterior is to the left, posterior is to the right, and dorsal is up. (B) Telomere intensity maps and stacked bar graphs for the representative images in (A) show that stem cells have the longest telomeres when compared with post-mitotic progeny. The intensity maps display the nuclei colored according to their telomere fluorescence intensity. The stacked bar graphs represent the proportion of nuclei within a given category of intensity. *smedwi-1* is chosen as the reference marker and set up to allocate in each range of intensity approximately one-fourth of the total cells. Magenta box indicates the region shown below and corresponds to the high magnifications in (A). (C) The column scatterplot shows all the pooled nuclei from the total of planarians and cells indicated; the median from *smedwi-1*^+^ cells is higher than that of *prog-1*^+^ cells (two-tailed Mann-Whitney U test; p < 0.0001), whereas the median from *prog-1*^+^ cells is higher than that of *agat-1*^+^ cells (two-tailed Mann-Whitney U test; p < 0.0001). The median of *smedwi-1*^+^ cells is the highest when compared with the other conditions (Kruskal-Wallis test; p < 0.0001). (D) The schematic represents the process of stem cell differentiation. The images show a representative subregion from one of the zones used for quantification. The telomere intensity map displays all the nuclei shown in the representative image colored according to their telomere fluorescence intensity. The population of *smedwi-1*^+^/SMEDWI-1^+^ has an increased number of stem cells with strong telomere intensity (either orange or red categories) when compared with its immediate progeny. (E) The graph shows all the nuclei pooled from all planarians analyzed (four zones per planarian). The median telomere intensity is lower in the SMEDWI-1^+^/*smedwi-1*^−^ when compared with SMEDWI-1^+^/*smedwi-1*^+^ (two-tailed Mann-Whitney U test; p < 0.0001). Arbitrary units are not comparable between (C) and (E) for being two independent experiments. tel, telomeres; n, number of planarians analyzed; a.u., arbitrary units of fluorescence. Scale bars, 1 mm (A and B, main images), 220 μm (A and B, high-magnification images), and 60 μm (D). See also Figure S2.

We first aimed to elucidate whether we could distinguish planarian stem cells from their progeny specifically by quantifying telomere length. To analyze those different cell populations, we performed FISH on planarian paraffin tissue sections using *smedwi-1* as a stem cell marker. To detect the non-dividing descendants of stem cells, we used two well-established differentiation assays in planarians. The first approach consisted in performing FISH for post-mitotic epidermal cell markers (e.g., *Smed-prog-1* and *Smed-agat-1*, which detect early and late post-mitotic descendant cells, respectively) (Eisenhoffer et al., 2008). We observed that the median telomere fluorescence intensity of *smedwi-1*^+^ cells is the highest when compared with early post-mitotic progeny *prog-1*^+^ cells and with late post-mitotic progeny *agat-1*^+^ cells (Figures 2A–2C, S2A, and S2B). At the same time, the median telomere fluorescence of *prog-1*^+^ cells is higher than that of *agat-1*^+^ cells (Figures 2B and 2C).

The second approach embraces all stem cell descendants and uses a combination of FISH for *smedwi-1* (mRNA) and immunohistochemistry for SMEDWI-1 (protein) (Guo et al., 2006). SMEDWI-1 (protein) is present in all *smedwi-1* mRNA^+^ dividing cells but also temporarily detectable in all post-mitotic descendant cells that are *smedwi-1*(mRNA)^−^ (Wenemoser and Reddien, 2010). Telomere fluorescence intensity quantification in *smedwi-1*^+^/SMEDWI-1^+^ cells (stem cells) and in *smedwi-1*^−^/SMEDWI-1^+^ cells (very early descendant cells) showed that stem cells display median telomere fluorescence intensity higher than their immediate descendants (Figures 2D, 2E, and S2C–S2E). This supports the results from the other approach and also extends its conclusions from epidermis to all types of descendant cells.

In summary, telomere fluorescence intensity correlates with the degree of differentiation of the cells, with stem cells having the highest median telomere intensity, late post-mitotic descendant cells displaying the lowest, and early post-mitotic or immediate descendants showing intermediate median telomere intensities.

### Planarian Germ Stem Cells Are a Subclass of *smedwi-1*^+^ Cells with the Longest Telomeres

Combined application of telomere length quantification with detection of specific cell markers has been shown to be useful in identifying novel stem cell populations in mice (Garcia-Lavandeira et al., 2009). Indeed, we observed that stem cell distribution of telomere fluorescence intensity shows a higher heterogeneity when compared with early and late post-mitotic progeny (Figure S2A). Similarly, the *smedwi-1*^+^/SMEDWI-1^+^ stem cell population shows higher heterogeneity when compared with their immediate progeny *smedwi-1*^−^/SMEDWI-1^+^ population (Figure S2C). The data predicts that quantification of telomere length could be a useful technique to localize new subpopulations into the *smedwi-1*^+^ stem cell pool, such as PSCs.

**Figure 3:**
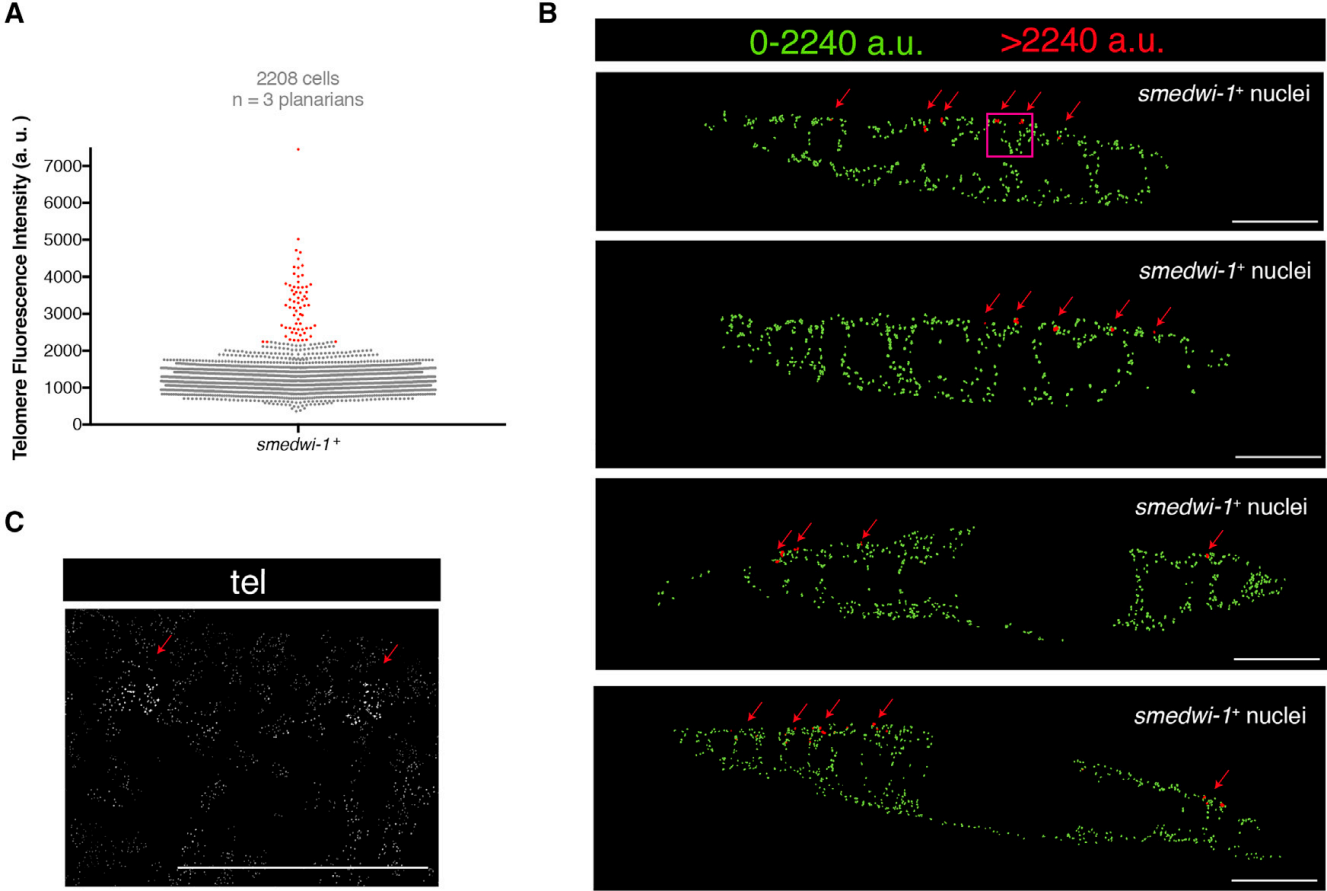
The Top 3% in Telomere Intensity of *smedwi-1*^+^ Cells Are Distributed Laterodorsally along the Whole Planarian Length (A) The column scatterplot shows all the pooled *smedwi-1*-positive nuclei. In red are the top 3% of cells in telomere intensity. (B) All sections quantified in (A) displayed as telomere intensity maps for *smedwi-1*^+^ stem cells. In red are the stem cells with the longest telomere intensity (top 3% in A; red arrows). Anterior is to the left, dorsal is up. The magenta box indicates the region in (C). (C) The telomeres with the strongest intensity (red arrows) are easily distinguishable. Scale bars, 200 μm (main images) and 30 μm (high-magnification images).

**Figure 4:**
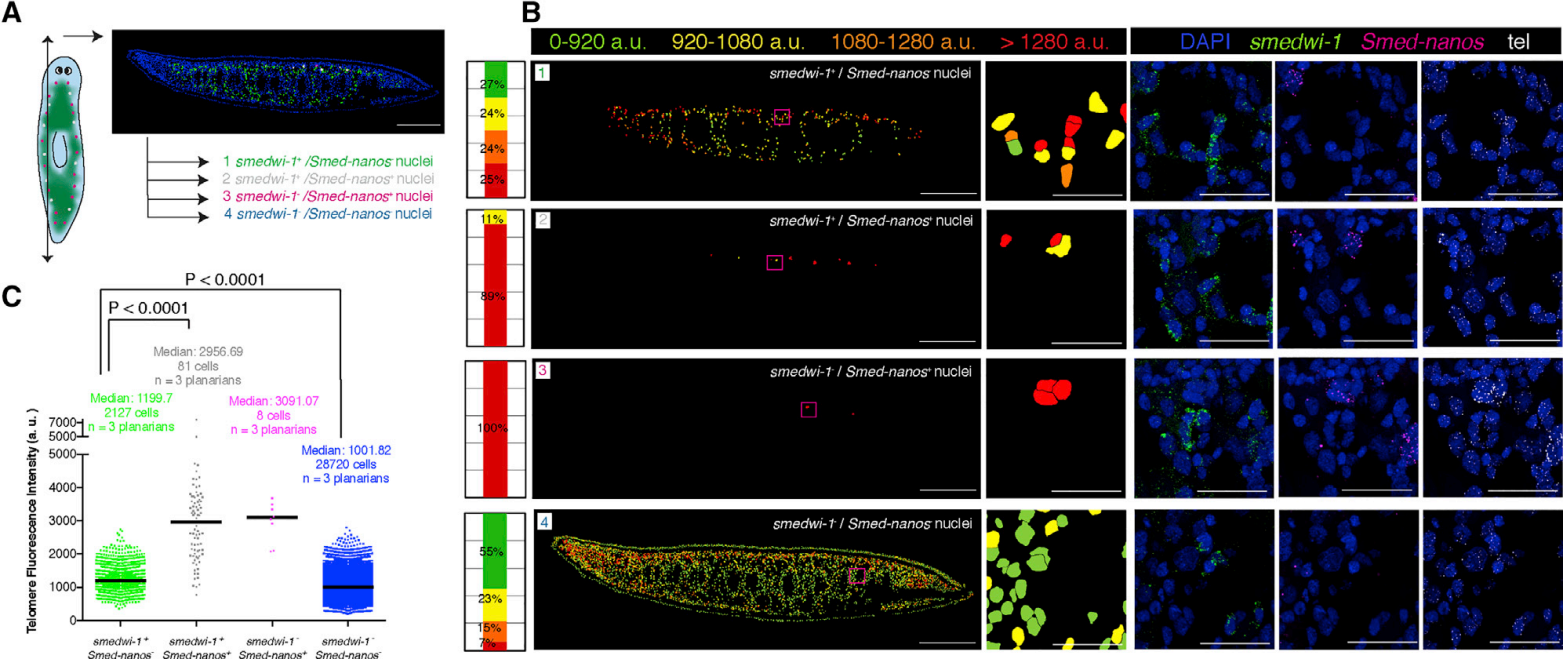
The Stem Cells with the Highest Telomere Intensity Are *nanos*^+^ (A) Schematic and tissue section indicate the distribution of four cell populations in a 7dS planarian. Double arrow indicates plane of sagittal sectioning. (B) Representative tissue section with four telomere intensity maps that represent the four cell populations displayed in (A). The intensity maps show the nuclei colored according to their telomere fluorescence intensity. The stacked bar graphs represent the proportion of nuclei within a given category of intensity. *smedwi-1*^+^*/nanos*^−^ is chosen as the reference population. Most of the stem cells with the longest telomere intensity are *nanos*^+^/*smedwi-1*^+^. Magenta box indicates the area shown. Anterior is to the left and dorsal is up. (C) The column scatterplot shows all the pooled nuclei broken down into four cell populations. Any of the three subclasses of stem cells display longer telomeres than non-stem cells *smedwi-1*^−^/*nanos*^−^ (two-tailed Mann-Whitney U test; p < 0.0001 for each of the three combinations). The median telomere intensity is higher in the *smedwi-1*^+^/*Smed-nanos*^+^ when compared with the *smedwi-1*^+^/*nanos*^−^ (two-tailed Mann-Whitney U test; p < 0.0001). n, number of planarians analyzed. Scale bars, 200 μm (main images) and 30 μm (high-magnification images). See also Figure S3.

While performing telomere quantification of *smedwi-1*^+^ cells, we noticed a subset of cells that had extremely high intensities of telomere fluorescence that stood out under a microscope with low magnification (Video S2). These were scattered cells in the dorsolateral parts of the planarian and distributed along the whole anteroposterior axis. We estimated that those cells represent approximately 3% of the total amount of *smedwi-1*^+^ cells in the planarian, since taking the top 3% in intensity values of a pool of *smedwi-1*^+^ cells from three planarians all displayed the dorsolateral distribution along the entire animal (Figure 3). Remarkably, the distribution of those rare cells resembled the expression pattern of the ortholog of the germline marker *nanos* in planarians (Handberg-Thorsager and Salo, 2007, Sato et al., 2006, Wang et al., 2007). Therefore, we hypothesized that the stem cells with the longest telomeres are *nanos*^+^ cells. To address this, we performed double FISH for *smedwi-1* and *nanos* and subsequently TelQ-FISH on paraffin sections (Figures 4A and 4B). As expected, telomere fluorescence intensity quantification showed that the stem cells positive for both *smedwi-1* and *nanos* have a higher median telomere intensity than the rest of the *smedwi-1*^+^ cells that are negative for *nanos* (Figure 4C). Of note, if we compare the cells negative for both markers, i.e., the cells in the planarians that are not stem cells, with any of the stem cell groups, we see that any of the three subclasses of stem cells display longer telomeres than non-stem cells (Figures 4B and 4C).

Video S2. The Highest Telomere Intensity in a Given Tissue Section Can Be Easily Observed The video shows a zoom into the dorsal part of a tissue section. The brightest telomeres can be easily seen (arrows).

Recently, single-cell RNA sequencing of *smedwi-1*^+^ cells has uncovered a subpopulation that contains PSCs among other stem cells (Zeng et al., 2018). To investigate its median telomere length we used the marker *Smed-tgs-1*, which defines that subpopulation and represents 25% of the total *smedwi-1*^+^ stem cells (Zeng et al., 2018) (Figure S3A). Double FISH for *tgs-1* and *smedwi-1* followed by TelQ-FISH showed no significant differences between the *smedwi-1*^+^/*tgs-1*^+^ and *smedwi-1*^+^/*tgs-1*^−^ subpopulations (Figures S3B–S3D) even after removing the *smedwi-1*^+^ cells with the longest telomeres from the comparison (Figure S3C).

### Starvation Increases the Percentage of Stem Cells with Long Telomeres through mTOR Signaling

**Figure 5:**
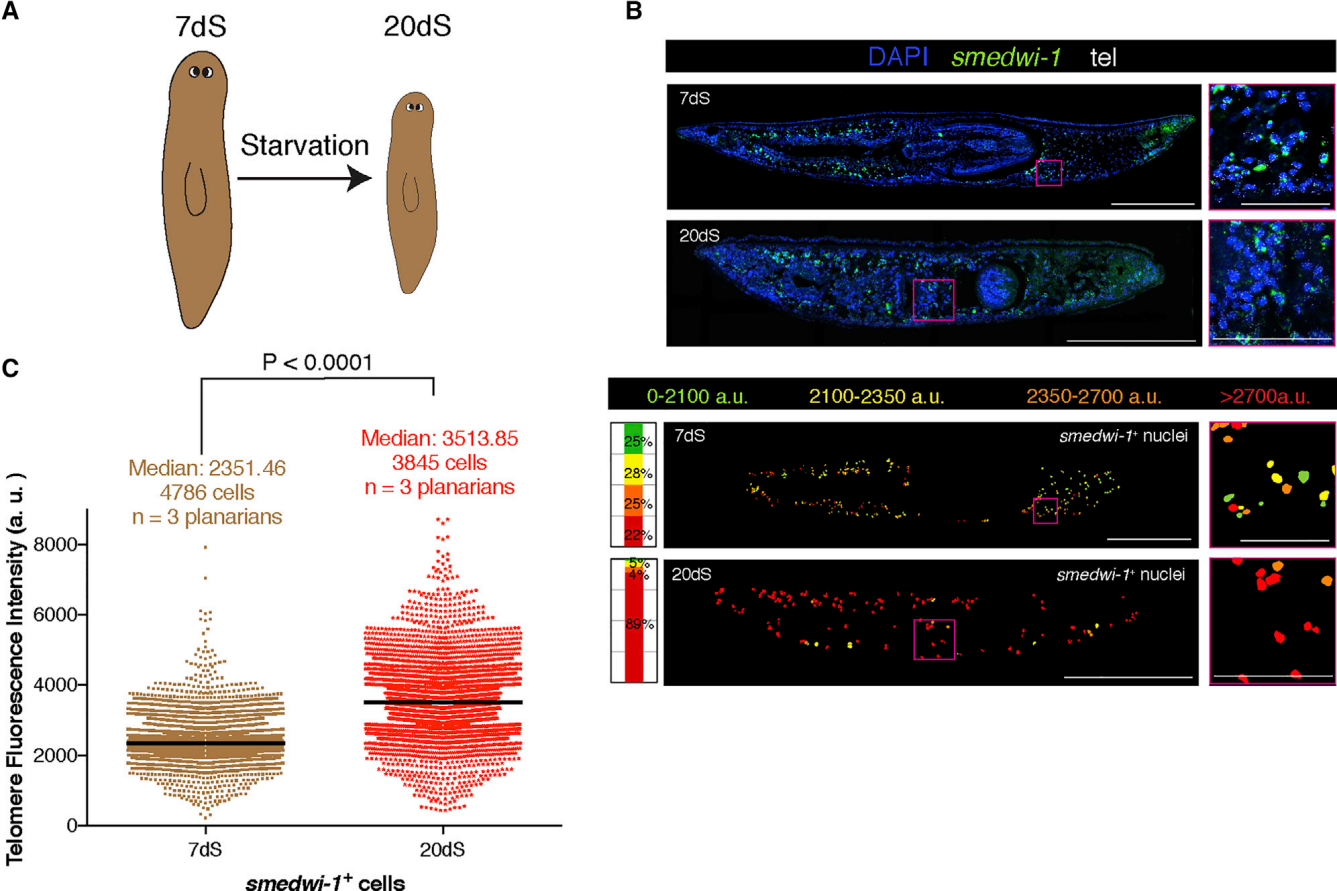
Starvation Increases the Percentage of Stem Cells with Long Telomeres (A) During the process of starvation, planarians degrow in size. (B) Maximum confocal projections for representative tissue sections of 7dS and 20dS planarian paraffin sections. Magenta box indicates the area shown. tel, telomeres; anterior is to the left and dorsal is up. Telomere intensity maps and stacked bar graphs for the representative tissue sections are also shown. The intensity maps display the nuclei colored according to their telomere fluorescence intensity. The stacked bar graphs represent the proportion of nuclei within a given category of intensity. 7dS is chosen as the reference condition. Magenta box indicates the area shown. Scale bars, 1 mm (main images) and 220 μm (high-magnification images). (C) Column scatterplot showing all pooled nuclei. The median telomere intensity is higher in 20dS than in 7dS stem cells (two-tailed Mann-Whitney U test; p < 0.0001). n, number of planarians analyzed. See also Figure S4.

To address how starvation affects stem cell biology, we studied telomere length on *smedwi-1*^+^ cells comparing planarians that were starved for 7 days (7dS; standard metabolic status used to avoid background staining by food) with planarians that were starved for 20 days (20dS) (Figure 5A). Telomere fluorescence intensity quantification in *smedwi-1*^+^ cells of 7dS planarians and in *smedwi-1*^+^ of 20dS planarians showed significantly longer telomeres in 20dS planarian stem cells than those of planarians at 7dS, as reflected by higher median telomere fluorescence intensity (Figures 5B and 5C). Two alternative approaches to measure telomere length, telomere qPCR on whole-planarian genomic DNA (Figure S4A) and TelQ-FISH on X1 (stem cells) sorted cells (Figures S4B and S4C), confirmed the initial results showing that the median telomere fluorescence intensity of stem cells at 20dS is higher than that at 7dS.

During the process of starvation, planarians degrow in size (Gonzalez-Estevez et al., 2012a). To distinguish whether the observed effects of starvation on telomeres are due to the metabolic process itself or to the smaller size of the planarian, we compared size-matched planarians that were either at 7dS or 30dS. The median telomere fluorescence intensity of *smedwi-1*^+^ cells at 30dS was higher than that of 7dS planarians (Figures S4D–S4F), similarly as with planarians of different metabolic status and size. In conclusion, starvation increases the percentage of stem cells with the longest telomeres.

**Figure 6:**
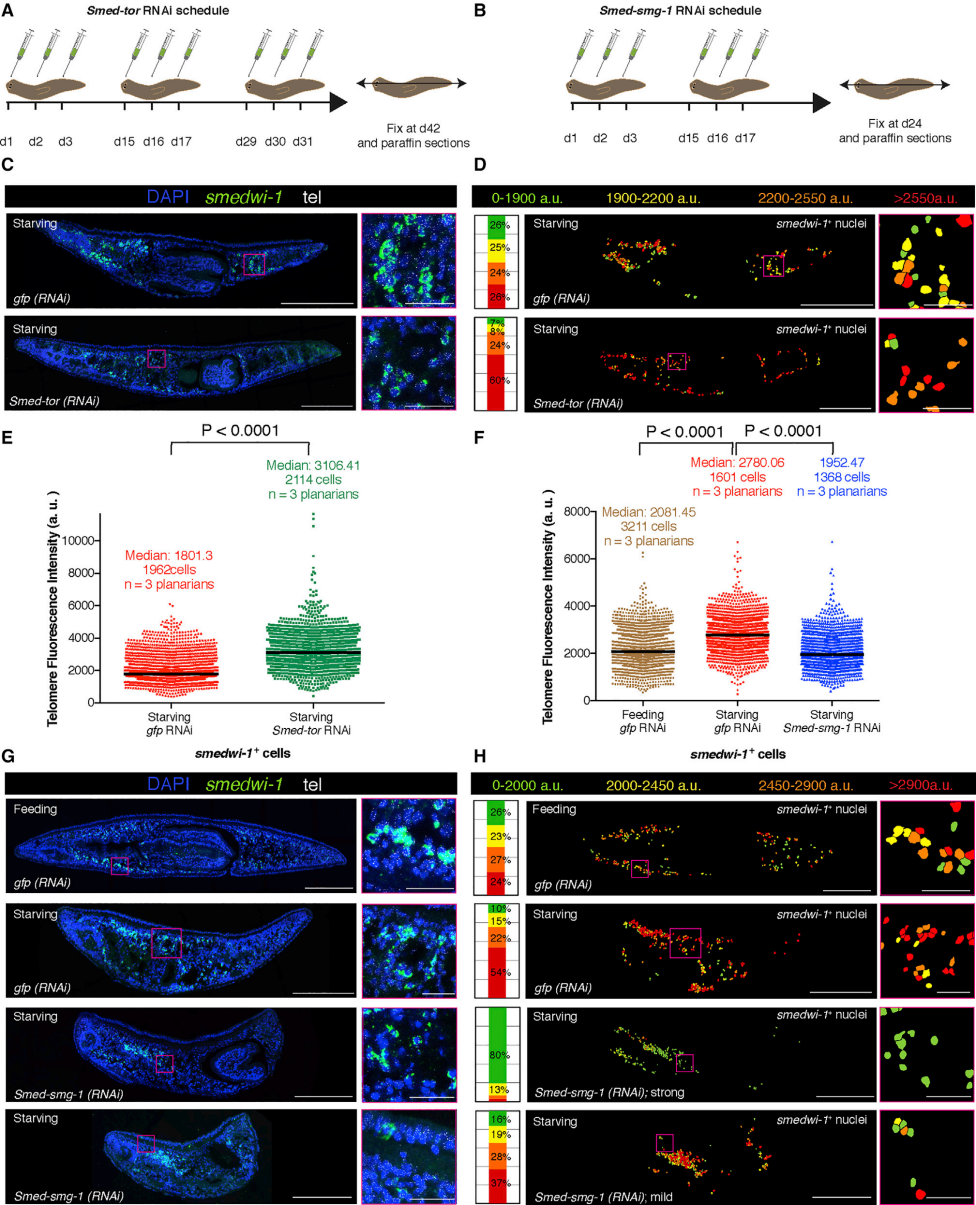
mTOR Signaling Modulates the Effects of Starvation on Stem Cell Telomeres (A) RNAi schedule for *tor* RNAi and controls. (B) RNAi schedule for *smg-1* RNAi and controls. (C) Maximum confocal projections for representative tissue sections of control planarians (starving) and *tor* RNAi (starving). Magenta box indicates the area shown. (D) Telomere intensity maps and stacked bar graphs for the *smedwi-1*^+^ cells in the representative tissue section shown in (C). The maps display the nuclei colored according to their telomere fluorescence intensity. The stacked bar graphs represent the proportion of nuclei within a given category of intensity. *gfp* RNAi is chosen as the reference condition. *tor* RNAi have a higher percentage of stem cells with long telomeres. Magenta box indicates the area shown. (E) Column scatterplot showing all the pooled cells. The median telomere intensity is higher in *tor* RNAi than in controls (two-tailed Mann-Whitney U test; p < 0.0001). (F) Column scatterplot showing all the pooled cells. The median telomere intensity is higher in controls starving than in controls feeding (two-tailed Mann-Whitney U test; p < 0.0001). *smg-1* RNAi (starving) abolishes the effects of starvation (two-tailed Mann-Whitney U test; p < 0.0001). *smg-1* RNAi (starving) has an even lower median than fed controls (two-tailed Mann-Whitney U test; p < 0.0001). Arbitrary units are not comparable between (E) and (F) for being two independent experiments. (G) Maximum confocal projections for representative tissue sections of control planarians either under starving or feeding conditions and *smg-1*(*RNAi*) planarians under starving conditions. Two images are displayed for *smg-1* RNAi (starving), which represent different degrees of phenotype penetrance. Magenta box indicates the area shown. (H) Telomere intensity maps and stacked bar graphs for the *smedwi-1*^+^ cells in the representative tissue sections shown in (G). The maps display the nuclei colored according to their telomere fluorescence intensity. The stacked bar graphs represent the proportion of nuclei within a given category of intensity. *gfp* RNAi (feeding) is chosen as the reference. *gfp* RNAi (starving) shows a higher percentage of stem cells with long telomeres, whereas this effect is abolished when planarians are injected with *smg-1* RNAi. Magenta box indicates the area shown. tel, telomeres; n, number of planarians analyzed; anterior is to the left and dorsal is up. Scale bars, 200 μm (main images) and 30 μm (high-magnification images). See also Figures S5 and S6.

mTOR signaling is the major nutrient sensor pathway that controls cell growth and proliferation in eukaryotes (Saxton and Sabatini, 2017). While inhibition of mTOR complex 1 (mTORC1) with rapamycin increases the life span of many organisms and delays many age-associated diseases, its upregulation has been strongly linked to the progression of many cancers and to the aging process (Cornu et al., 2013). Since reduction of mTOR signaling has also shown to be one of the most important mechanisms responsible for the enhancement of stem cell function after DR (Huang et al., 2012, Yilmaz et al., 2012), we reasoned that mTOR signaling could be responsible for the increase in the percentage of stem cells with long telomeres during starvation that we observed. To test this theory, we performed RNAi experiments for the main components of mTOR signaling followed by FISH for *smedwi-1* and TelQ-FISH. Planarians were either injected with *Smed-tor* double-stranded RNA (dsRNA) (to inactivate the mTOR pathway) (Gonzalez-Estevez et al., 2012b, Peiris et al., 2012, Tu et al., 2012) or with *Smed-smg-1* dsRNA (to overactivate the mTOR pathway) (Gonzalez-Estevez et al., 2012b). Only planarians without apparent morphological phenotype were processed for telomere quantification to avoid loosening integrity in the tissues during the aggressive TelQ-FISH protocol (Figures 6A, 6B, S5A, and S5B). However, they already displayed a significant decrease in the number of mitoses in the case of *tor* RNAi (Figures S5C and S5D), and an increase in the case of *smg-1* RNAi (Figures S5E and S5F). Downregulation of *tor* further enhanced the effect of starvation on stem cell telomere length when compared with control injected worms (Figures 6C–6E). We also noticed that downregulation of *tor* is able to increase the maximum telomere length in stem cells (Figure 6E). This effect was also observed when comparing 7dS with 20dS (Figure 5C). This suggests that starvation and/or mTOR downregulation leads some stem cells to elongate their telomeres.

Remarkably, when comparing *smg-1* RNAi under starvation with starved or fed control planarians (Figure 6F), we observed that the telomere length distribution in *smg-1*(*RNAi*) stem cells is more similar to the controls in feeding conditions. Thus *smg-1* RNAi abolishes the effects of starvation on telomere elongation in stem cells. Furthermore, *smg-1* RNAi under feeding conditions (Figures S5G and S5H) only shows a slight decrease in the median telomere length with respect to fed controls (Figures 6F–6H), which suggests that the effects of *smg-1* RNAi on telomere length that are independent of the planarian caloric status are minimal. In agreement with the phenotype of *smg-1* RNAi showing different degrees of penetrance in planarians (Gonzalez-Estevez et al., 2012b), we also obtained planarians in which the effects of starvation were either just slightly or strongly abolished (Figure 6H).

We also performed telomere qPCR on *gfp*(*RNAi*) and *tor*(*RNAi*) whole-planarian genomic DNA (Figure S6A) and TelQ-FISH on X1 (stem cells) sorted cells from *gfp*(*RNAi*) and *tor*(*RNAi*) planarians (Figures S6B and S6C), which confirmed our previous results showing that the median telomere fluorescence intensity of stem cells in *tor* RNAi is higher than that of controls.

We conclude that downregulation of *tor* enhances the effects of starvation on stem cell telomere length, while downregulation of *smg-1* (which increases mTOR signaling) does the opposite, abolishing the effects of starvation on telomere length in stem cells.

## Discussion

Telomere quantitative FISH has been widely used in a variety of tissues and organisms such as yeast, plants, zebrafish, or diverse mouse/human tissues. Planarians are an addition, and we envisage a wide use of the technique to study many aspects of aging, stem cell biology, DNA damage, and stress in the planarian model. Our work represents the first quantitative mapping of telomere length in whole planarians including in planarian stem cells. Other *in vitro* experimental telomere quantitative technologies such as Southern blot analysis of terminal restriction fragment lengths have been previously applied to whole planarians (Tan et al., 2012) but cannot provide any detail about location and cell type. We have also adapted telomere length quantification to FACS-sorted cells as an alternative method to complement the data on paraffin tissue sections. Furthermore, telomere qPCR represents an easy method to confirm overall results.

Telomere length has been utilized to localize stem cell compartments in diverse mouse stem cell tissues (Aida et al., 2008, Flores et al., 2008). Our work demonstrates that telomere length quantification is able to distinguish between planarian stem cells and their progeny, as well as progeny of different ages. Our work sheds new light on a subclass of stem cells that co-express *smedwi-1*, a marker of somatic stem cells and *nanos*, a marker of germ cells in metazoans (Kobayashi et al., 1996, Koprunner et al., 2001). In sexually reproducing *S*. *mediterranea*, *nanos* is necessary for the development, maintenance, and regeneration of the germline but is also expressed in the asexually reproducing *S*. *mediterranea* strain (Handberg-Thorsager and Salo, 2007, Sato et al., 2006, Wang et al., 2007). Although the distribution of *nanos* in asexual planarians follows that of presumptive testes primordia of sexual planarians, asexually reproducing planarians will never differentiate gonads. The reason why putative germ stem cells exist in exclusively asexually reproducing planarians is currently unknown. One explanation suggested by previous studies is that these cells are vestigial remains of sexual reproduction, which have not yet been lost. However, it is well established that stem cells and germ cells are very alike; morphologically both look the same (Sato et al., 2006) and both share the expression of multiple regulators of germ cell development and known regulators of stem cell pluripotency (reviewed in Rink, 2013). In addition, we observe that *nanos* expression co-localizes with *smedwi-1* in the asexual strain. This is in agreement with *nanos*^+^ cells in the asexual species *Dugesia japonica* co-labeling with the proliferative marker PCNA (Sato et al., 2006). In line with the conservative definition of germ cells as unipotent cells that give rise to one type of gamete (Yuan and Yamashita, 2010), it is currently thought that planarian germ cells (*nanos*^+^ cells) arise from somatic stem cells and represent a more differentiated type of neoblast (Sato et al., 2006, Wang et al., 2007). If we consider telomere length as a tool to locate the more primitive stem cell compartments, an alternative model arises in which *nanos*^+^ cells, which have the longest telomeres of all cells, would correspond to PSCs that could give rise to the rest of neoblasts in a hierarchical manner. It is a formal possibility that *nanos*^+^ cells could be arrested, quiescent, or reserved stem cells that would contribute to the whole pool of somatic stem cells only in some contexts when they become activated. Since *nanos*^+^ cells do not become mature germ cells, we would expect that the arrest of a Tert^+^ cell could lead to elongation of telomeres beyond that which would be seen in sexual animals. Further experiments are needed to clarify whether *nanos*^+^ cells can give rise to somatic stem cells.

We also analyzed the distribution of telomere length in the stem cells positive for the marker *tgs-1*, which is known to belong to a subpopulation that represents 25% of the total *smedwi-1*^+^ stem cells in the planarian body and contains PSCs among other stem cells (Zeng et al., 2018). Our data showed that both populations of stem cells *smedwi-1*^+^/*tgs-1*^+^ and *smedwi-1*^+^/*tgs-1*^−^ have the same telomere distribution and that *smedwi-1*^+^/*tgs-1*^+^ cells do not include the stem cells with the longest telomeres. Since it is not known how many stem cells positive for *tgs-1* are truly pluripotent, it could still be that *tgs-1*^+^ stem cells with the longest telomeres are pluripotent or can go back to be pluripotent in some contexts. Future studies involving *in vivo* experiments will need to address whether planarian stem cells with the longest telomeres are PSCs.

DR leads to maintenance or elongation of telomeres and increased telomerase activity in various adult mouse tissues (Vera et al., 2013). However, there is currently no evidence that DR or fasting may be influencing telomere length specifically on stem cells and thus contributing to the reported increased stem cell functionality after dietary intervention. Here we show that starvation increases the population of stem cells with long telomeres in the planarian model and that at least some stem cells are able to significantly elongate telomere length at 20dS when compared with 7dS. Considering that planarians maintain a stable pool of dynamically dividing stem cells and reduce the rate of differentiation over the course of starvation (Gonzalez-Estevez et al., 2012a), increasing telomere length seems a good strategy to avoid an increased risk of stem cell aging and, thus, aging in the whole planarian. Remarkably, previous work by means of Southern blot analysis on planarian genomic preparations has shown that global average telomere length increases after regeneration or fission following a long period of no regeneration/fission in asexual planarians (Tan et al., 2012). Thus, through telomere maintenance during regeneration/fission and starvation, two processes that are continuously occurring during planarian live history, planarians may be able to maintain a “young” population of stem cells, and therefore to be considered immortal.

Although downregulation of mTOR signaling is the major mechanism for the enhancement of stem cell function after DR (Huang et al., 2012, Yilmaz et al., 2012), only a few works suggest that mTOR signaling could be coupled to telomerase and telomere length regulation (Kawauchi et al., 2005). Our data show that while downregulation of mTOR enhances the effect of starvation on the telomeres of planarian stem cells, upregulation of mTOR signaling (by downregulating of the negative regulator of mTOR signaling, SMG-1) is able to abolish these effects. In planarians, mTOR signaling is a key regulator of the first response to wounding during regeneration (Gonzalez-Estevez et al., 2012b, Peiris et al., 2012, Tu et al., 2012). However, there are few data about how mTOR signaling regulates starvation in planarians. mTOR signaling is downregulated during starvation as observed in other organisms (Peiris et al., 2012). RNAi experiments on members of mTORC1 or rapamycin treatment during starvation have shown a reduction in the number of mitotic neoblasts and in the whole pool of *smedwi-1*^+^ stem cells (Gonzalez-Estevez et al., 2012b, Peiris et al., 2012). In a similar way, downregulation of planarian PTEN homologs (negative regulators of the pathway) increases the number of mitoses in starved planarians (Oviedo et al., 2008). Here we have also shown that while *tor* RNAi reduces the number of mitoses by day 42 of starvation, *smg-1* RNAi already increases mitoses by 24 days. Since telomeres shorten progressively with every cell division (Flores and Blasco, 2010), one possible explanation is that the telomere effects that we see here could be a secondary consequence of stem cell perturbation rather than a consequence of changes in mTOR signaling. However, while during starvation telomere length increases, which we observed is dependent on mTOR signaling, the number of mitoses and stem cells are maintained (Gonzalez-Estevez et al., 2012a). Indeed, it is known that other factors modulate telomere length, for instance oxidative stress, oxygen levels, and, in general, mild stress (Flores et al., 2008, von Zglinicki, 2002, von Zglinicki et al., 1995). Together these findings make feasible the idea of starvation and mTOR signaling directly regulating telomere length by other mechanisms than cell division. Any of these factors could also explain our results of the shortening of median telomere length from late to early post-mitotic progeny, two cell planarian populations that transition without cell division.

It has also been described that *tor*(*RNAi*) planarians are still able to degrow at the same rate as controls while having increased levels of cell death (Peiris et al., 2012), which indicates that some stem cells are dying. Remarkably our data show that the subpopulation of stem cells with short telomeres reduces during starvation (downregulation of mTOR) and disappears after *tor* RNAi (stronger downregulation of mTOR) (Figure S6D). A number of different models could explain how mTOR activity regulates the stem cell population telomere length distribution. These include the direct regulation of telomerase activity or through regulating which stem cells divide symmetrically or asymmetrically using existing telomere length as one of the inputs to this decision. Since mTOR signaling can determine fitness through cell competition (Bowling et al., 2018, Claveria and Torres, 2016), another possibility is that the stem cells with shorter telomeres are more likely to die/differentiate when mTOR activity is reduced on average while stem cells with long telomeres are competitively selected to self-renew. Future work using the experimental paradigm we establish here will provide further insight into how nutritional status affects adult stem cell biology.

## Experimental Procedures

### Animal Husbandry

Planarians in this work belong to the species *S. mediterranea* asexual strain and were maintained at 19°C in 1× Montjuïc salts (1.6 mM NaCl, 1.0 mM CaCl_2_, 1.0 mM MgSO_4_, 0.1 mM MgCl_2_, 0.1 mM KCl, 1.2 mM NaHCO_3_). Planarians were fed organic veal liver.

### RNAi Experiments

We generated templates with T7 promoters appended to both strands from *Smed-smg-1*, *Smed-tor*, and *Smed-tert*, synthesized dsRNA by *in vitro* transcription (Roche), and injected these into planarians as described previously (Gonzalez-Estevez et al., 2012b, Tan et al., 2012). Control animals were injected with *gfp* dsRNA, a sequence not present in *S*. *mediterranea*. A detailed protocol can be found in Supplemental Experimental Procedures.

### *In Situ* Hybridization on Planarian Sections

Single and double FISH were performed as previously described (Solana, 2018). A detailed protocol can be found in Supplemental Experimental Procedures.

### Immunohistochemistry after FISH on Planarian Sections

Immnunohistochemistry after FISH was performed as described by Solana (2018). A mouse polyclonal SMEDWI-1 antibody was generated using an already reported peptide (Guo et al., 2006) and affinity purified (GenScript). The antibody was used at a 1/100 dilution. An AlexaFluor 488 goat anti-mouse immunoglobulin G (H + L) Antibody (Life Technologies) was used as secondary antibody.

### Telomere Quantitative Fluorescent *In Situ* Hybridization

TelQ-FISH, image acquisition, and quantifications were performed as previously described for mouse tissue sections (Flores et al., 2008, Gonzalez-Suarez et al., 2000, Zijlmans et al., 1997). For a detailed protocol please refer to Supplemental Experimental Procedures.

### Statistical Analysis

Since most of the TelQ-FISH samples analyzed in herein (26/28) failed the D'Agostino and Pearson normality test (only *smedwi-1*^−^/SMEDWI-1^+^ [p = 0.4590] and *smedwi-1*^−^/*Smed-nanos*^+^ [p = 0.5591] passed the test) and neither followed a log-normal distribution, we used non-parametric statistical tests to evaluate significance. Kruskal-Wallis one-way analysis of variance and/or two-tailed Mann-Whitney U test was used to evaluate significance. Two-tailed Student's t test with equal sample variance was used for telomere qPCR and H3P quantification; error bars represent standard deviation of the mean. All the tests were performed with GraphPad Prism 7.0d.

## Author Contributions

M.I. and C.G.-E. performed most of the experiments; D.A.F., O.G.-G., S.S., and A.A.A. helped to perform some experiments; B.F.-V. and R.P. performed the telomere qPCRs; M.I., I.F., and C.G.-E. designed experiments and analyzed the data; C.G.-E. performed most of the telomere quantification analyses with the help of D.A.F. and O.G.-G.; M.d.M.d.M.B. contributed to the initial setting of planarian telomapping and obtained some preliminary data; I.F. and C.G.-E. directed the project; C.G.-E. wrote the manuscript; M.I., D.A.F., O.G.-G., A.A.A., and I.F. contributed to the editing of the manuscript.

## Supplemental Information

Document S1. Supplemental Experimental Procedures and Figures S1–S6

Document S2. Article plus Supplemental Information
