# Supplementary material for: Downregulation of mTOR Signaling Increases Stem Cell Population Telomere Length during Starvation of Immortal Planarians
Source: Stem Cell Reports. 2019 Jul 25;13(2):405–18. doi: 10.1016/j.stemcr.2019.06.005 (PMC6700675; doi:10.1016/j.stemcr.2019.06.005)
Supplement: Document S1. Supplemental Experimental Procedures and Figures S1–S6 [file mmc1.pdf]

Stem Cell Reports, Volume *13*

Supplemental Information

Downregulation of mTOR Signaling Increases Stem Cell Population Telomere Length during Starvation of Immortal Planarians

Marta Iglesias, Daniel A. Felix, Óscar Gutiérrez-Gutiérrez, Maria del Mar De Miguel-Bonet, Sounak Sahu, Beatriz Fernández-Varas, Rosario Perona, A. Aziz Aboobaker, Ignacio Flores, and Cristina González-Estévez

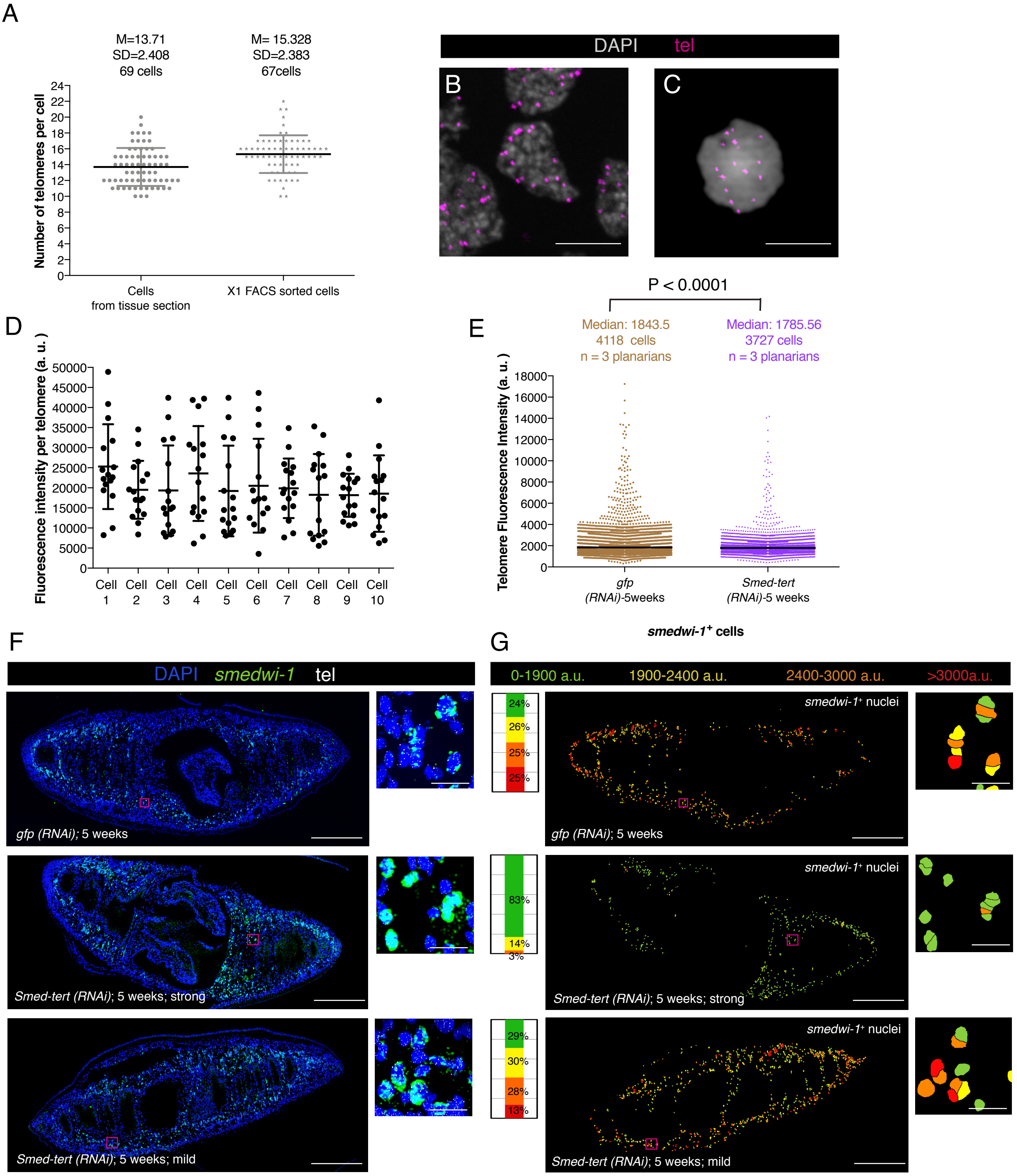
A
M=13.71
SD=2.408
69 cells
M= 15.328
SD=2.383
67cells
Number of telomeres per cell
Cells
from tissue section
X1 FACS sorted cells
DAPI tel
B
C
D
Fluorescence intensity per telomere (a. u.)
Cell 1
Cell 2
Cell 3
Cell 4
Cell 5
Cell 6
Cell 7
Cell 8
Cell 9
Cell 10
E
P < 0.0001
Median: 1843.5
4118 cells
n = 3 planarians
Median: 1785.56
3727 cells
n = 3 planarians
Telomere Fluorescence Intensity (a. u.)
gfp (RNAi)-5weeks
Smed-tert (RNAi)-5 weeks
F
DAPI smedwi-1 tel
gfp (RNAi); 5 weeks
Smed-tert (RNAi); 5 weeks; strong
Smed-tert (RNAi); 5 weeks; mild
G
smedwi-1+ cells
0-1900 a.u.
1900-2400 a.u.
2400-3000 a.u.
>3000a.u.
24%
26%
25%
25%
83%
14%
3%
29%
30%
28%
13%
smedwi-1+ nuclei
gfp (RNAi); 5 weeks
Smed-tert (RNAi); 5 weeks; strong
Smed-tert (RNAi); 5 weeks; mild

Figure S1. Validation of TelQ-FISH in planarians. Related to Figure 1. (A) Quantification of the number of telomeres in cells of a tissue section and in FACS sorted stem cells (X1 fraction). The mean (M) number of telomeres per cell and the total number of cells analyzed is displayed. SD, standard deviation. (B) Image of cells from a tissue section. The cell in the middle of the image shows 16 telomeres. (C) Image of a FACS sorted stem cell (X1) showing 16 telomeres. Scale bars indicate 10 μm. (D) Column scatter plot showing the total fluorescence intensity of every telomere from 10 different FACS sorted stem cells (X1). The error bars indicate deviation from the mean. Cell 6 shows the highest spread of values (longest error bar) while cell 9 shows the lowest (shortest error bar). (E) Column scatter plot showing all the cells pooled from a total of 3 planarians for each condition. The median telomere intensity is higher in *gfp(RNAi)* than in *tert(RNAi)* stem cells (two-tailed Mann-Whitney U test; $P < 0.0001$). n indicates the number of planarians analyzed; a. u. indicates arbitrary units. (F) Maximum projections for representative tissue sections from E of *gfp* injected planarians and *tert(RNAi)* injected planarians labeled with the stem cell marker *smedwi-1*. All are also labeled for telomeres and counterstained for DAPI. All represent 5 weeks of RNAi treatment. Two images are displayed for *tert* RNAi which represent different degrees of phenotype penetrance (mild and strong). The magenta squares indicate the area of magnification displayed next to the main images. tel indicates telomeres; anterior is to the left and dorsal is up. Scale bars indicate 250 μm in the main images and 30 μm in the high magnification images. (G) Stacked bar graphs and telomere intensity maps for the *smedwi-1*$^{+}$ cells in the representative tissue sections showed in F. The maps display the nuclei coloured according to their telomere fluorescence intensity (four categories of intensity). The stacked bar graphs represent the proportion of nuclei within a given category of intensity. *gfp(RNAi)* condition is chosen as the reference condition and set up to allocate in each range of intensity or category approximately one fourth of the total cells. *tert(RNAi)* condition shows a higher percentage of stem cells with short telomeres and a lower percentage of stem cells with long telomeres than in controls. Different degrees in the *tert(RNAi)* phenotype are presented (mild and strong). Magenta squares indicate the area of magnification displayed next to the main images. a.u. indicates arbitrary units. Scale bars indicate 250 μm in the main images and 30 μm in the high magnification images.

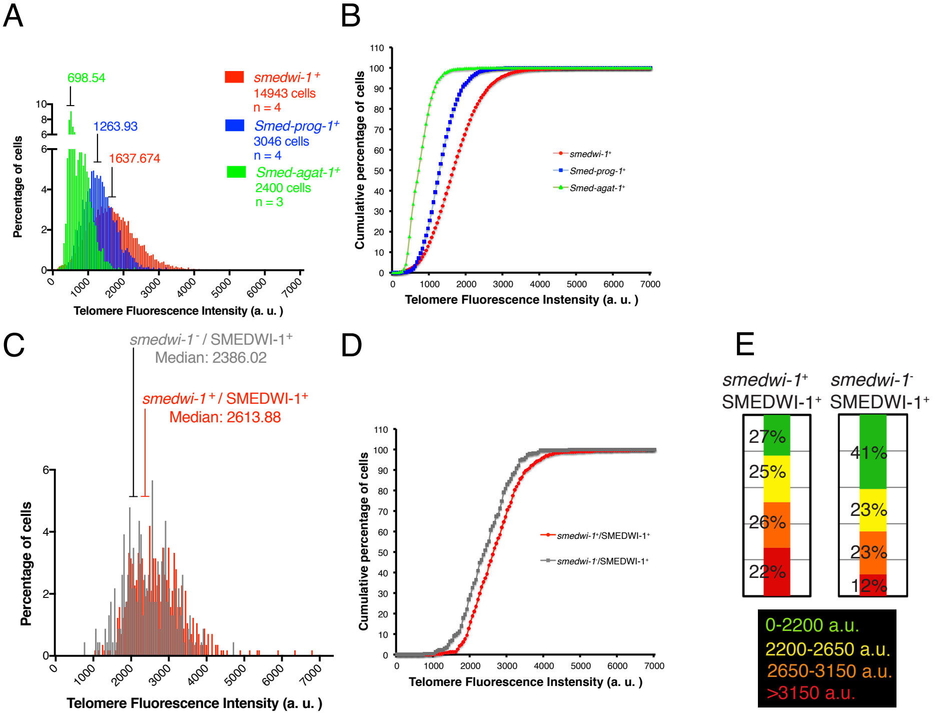

A
698.54
1263.93
1637.674
smedwi-1+
14943 cells
n = 4
Smed-prog-1+
3046 cells
n = 4
Smed-agat-1+
2400 cells
n = 3
Percentage of cells
Telomere Fluorescence Intensity (a. u.)
B
Cumulative percentage of cells
smedwi-1+
Smed-prog-1+
Smed-agat-1+
Telomere Fluorescence Instensity (a. u.)
C
smedwi-1- / SMEDWI-1+
Median: 2386.02
smedwi-1+ / SMEDWI-1+
Median: 2613.88
Percentage of cells
Telomere Fluorescence Intensity (a. u.)
D
Cumulative percentage of cells
smedwi-1+/SMEDWI-1+
smedwi-1-/SMEDWI-1+
Telomere Fluorescence Instensity (a. u.)
E
smedwi-1+
SMEDWI-1+
smedwi-1-
SMEDWI-1+
27%
25%
26%
22%
41%
23%
23%
12%
0-2200 a.u.
2200-2650 a.u.
2650-3150 a.u.
>3150 a.u.

Figure S2. Different ways of representing telomere intensity data. Related to Figure 2. (A-B) Frequency histogram (A) and cumulative frequency graph (B) showing the data displayed in Fig. 2C. Stem cell distribution of telomere fluorescence intensity shows a higher heterogeneity (σ^2=492936.355) when compared to early and late postmitotic progeny (σ^2=198232.864 and σ^2=90484.769, respectively) (C-D) Frequency histogram (C) and cumulative frequency graph (D) showing the data displayed in Figure 2E. The *smedwi-1*$^+$/SMEDWI-1$^+$ stem cell population (σ^2=502727.219) shows higher heterogeneity when compared to their immediate progeny *smedwi-1*$^-$/SMEDWI-1$^+$ population (σ^2=399559.689) (E) The stacked bar graph represent the proportion of nuclei within a given category of intensity for the data displayed in Figure 2E. *smedwi-1*$^+$/SMEDWI-1$^+$ is chosen as the reference condition and set up to allocate in each range of intensity or category approximately one fourth of the total cells. *smedwi-1*$^-$/SMEDWI-1$^+$ shows a higher percentage of cells with short telomeres and a lower percentage of cells with long telomeres when compared to the *smedwi-1*$^+$/SMEDWI-1$^+$ population.

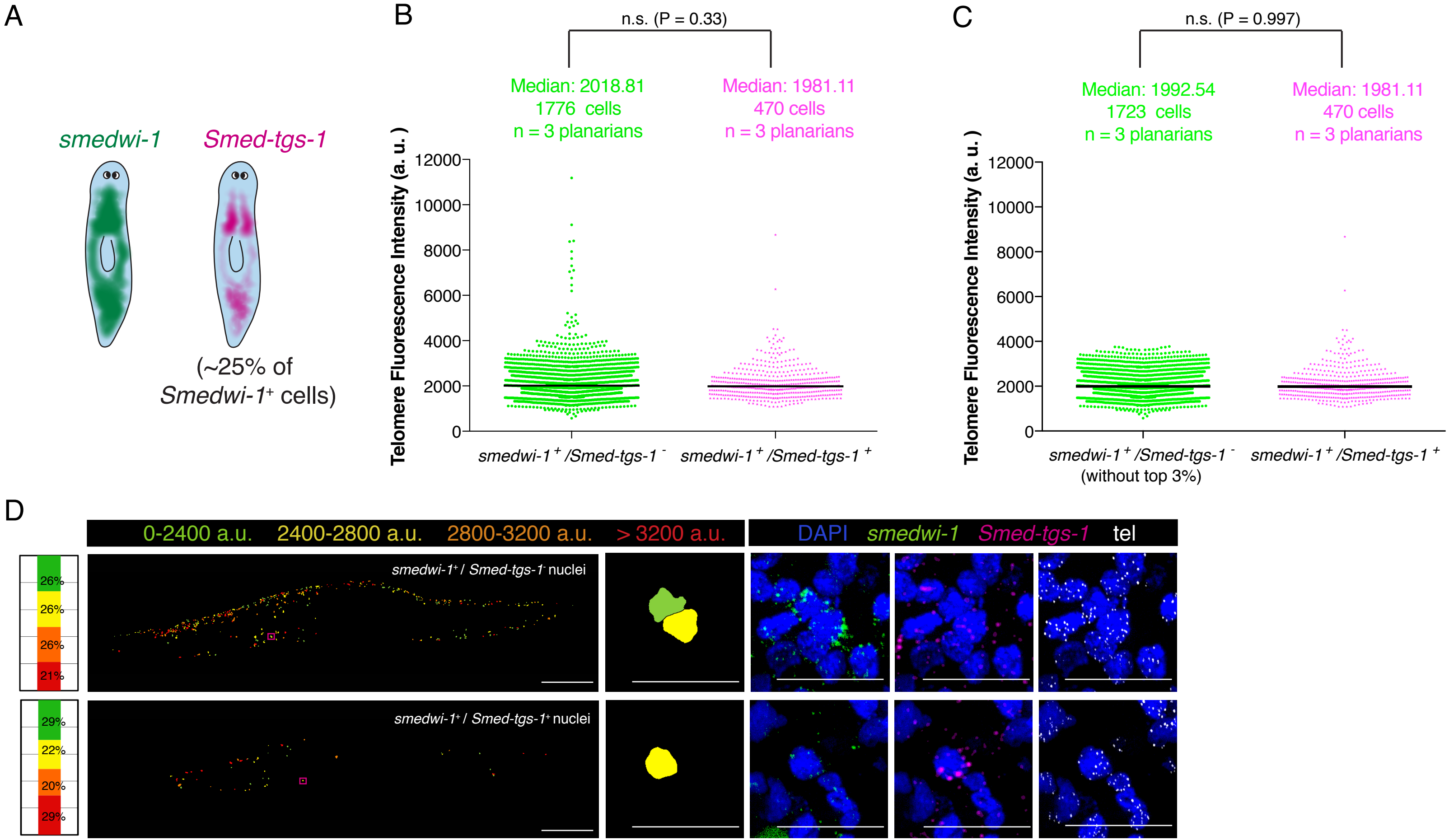
A
smedwi-1
Smed-tgs-1
(~25% of
Smedwi-1+ cells)
B
n.s. (P = 0.33)
Median: 2018.81
1776 cells
n = 3 planarians
Median: 1981.11
470 cells
n = 3 planarians
Telomere Fluorescence Intensity (a. u.)
12000
10000
8000
6000
4000
2000
0
smedwi-1+ /Smed-tgs-1-
smedwi-1+ /Smed-tgs-1+
C
n.s. (P = 0.997)
Median: 1992.54
1723 cells
n = 3 planarians
Median: 1981.11
470 cells
n = 3 planarians
Telomere Fluorescence Intensity (a. u.)
smedwi-1+ /Smed-tgs-1-
(without top 3%)
smedwi-1+ /Smed-tgs-1+
D
0-2400 a.u.
2400-2800 a.u.
2800-3200 a.u.
> 3200 a.u.
DAPI
smedwi-1
Smed-tgs-1
tel
26%
26%
26%
21%
smedwi-1+ / Smed-tgs-1- nuclei
29%
22%
20%
29%
smedwi-1+ / Smed-tgs-1+ nuclei

Figure S3. The *smedwi-1*$^+$ / *Smed-tgs-1*$^+$ population shows a similar telomere length distribution as the *smedwi-1*$^+$ /*Smed-tgs-1*$^-$ population. Related to Figure 4. (A) The schematic indicates the distribution of *smedwi-1*$^+$ cells (green) and *Smed-tgs-1*$^+$ cells (magenta) in a 7dS planarian. (B) Column scatter plot showing all the cells pooled from a total of 3 planarians for each condition. The median telomere intensity is not significantly different between *smedwi-1*$^+$/*Smed-tgs-1*$^-$ and *smedwi-1*$^+$/*Smed-tgs-1*$^+$ populations (two-tailed Mann-Whitney U test; P = 0.33). n indicates the number of planarians analyzed; a. u., arbitrary units; n.s., not significant. (C) Same column scatter plot as in B, this time comparing *smedwi-1*$^+$/*Smed-tgs-1*$^-$ cells which do not contain the top 3% of cells with the highest intensity values with the *smedwi-1*$^+$/*Smed-tgs-1*$^+$ population. There are not significant differences between both populations (two-tailed Mann-Whitney U test; P = 0.997). n indicates the number of planarians analyzed; a. u., arbitrary units; n.s., not significant. (D) Stacked bar graphs and telomere intensity maps from a representative tissue section of B broken down into two cell populations: *smedwi-1*$^+$/*Smed-tgs-1*$^-$ and *smedwi-1*$^+$/*Smed-tgs-1*$^+$ cells. The maps display the nuclei coloured according to their telomere fluorescence intensity (four categories of intensity). The stacked bar graphs represent the proportion of nuclei within a given category of intensity. *smedwi-1*$^+$/*Smed-tgs-1*$^-$ cells is chosen as the reference condition and set up to allocate in each range of intensity or category approximately one fourth of the total cells. Similar proportions are shown for both cell populations. Magenta squares indicate the area of magnification displayed next to the main images. a.u. indicates arbitrary units. Scale bars indicate 250 µm in the main images and 30 µm in the high magnification images.

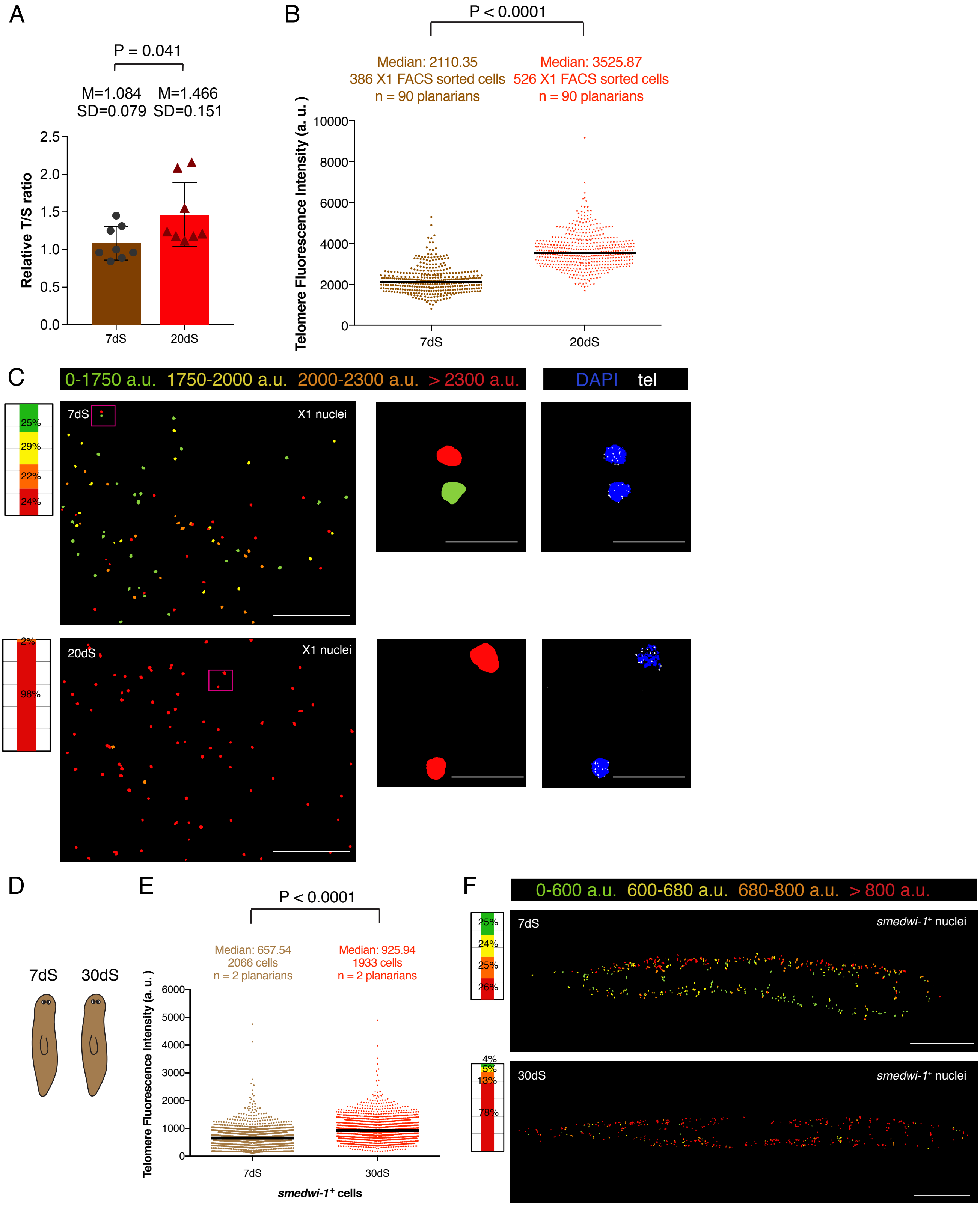
A
P = 0.041
M=1.084 M=1.466
SD=0.079 SD=0.151
Relative T/S ratio
7dS
20dS
B
P < 0.0001
Median: 2110.35
386 X1 FACS sorted cells
n = 90 planarians
Median: 3525.87
526 X1 FACS sorted cells
n = 90 planarians
Telomere Fluorescence Intensity (a. u.)
7dS
20dS
C
0-1750 a.u. 1750-2000 a.u. 2000-2300 a.u. > 2300 a.u.
DAPI tel
7dS
X1 nuclei
25%
29%
22%
24%
20dS
X1 nuclei
2%
98%
D
7dS
30dS
E
P < 0.0001
Median: 657.54
2066 cells
n = 2 planarians
Median: 925.94
1933 cells
n = 2 planarians
Telomere Fluorescence Intensity (a. u.)
7dS
30dS
smedwi-1+ cells
F
0-600 a.u. 600-680 a.u. 680-800 a.u. > 800 a.u.
7dS
smedwi-1+ nuclei
25%
24%
25%
26%
30dS
smedwi-1+ nuclei
4%
5%
13%
78%

Figure S4. Starved planarians show a higher percentage of stem cells with long telomeres than 7 days starved planarians, independently of body size. Related to Figure 5. (A) Telomere quantitative PCR (qPCR) comparing 7dS and 20dS whole planarians. qPCR was performed on genomic DNA for 8 biological replicates (5 planarians per replicate) per condition. The qPCR determines the ratio of telomere (T) repeat copy number to a single-copy (S) gene (genomic DNA from dd_Smed_v6_2426_0_1) copy number (T/S ratio) compared with a reference DNA sample at 7dS. The graph shows that 20dS planarians have a higher T/S ratio than 7dS (two-tailed Student's t-test with equal sample variance, $P < 0.05$) and thus higher telomere length. M, mean; SD, standard deviation. (B) Column scatter plot showing X1 FACS sorted cells that come from planarians at 7dS and 20dS (90 planarians per condition were used for the FACS). The median telomere intensity is higher in stem cells from 20dS than from 7dS planarians (two-tailed Mann-Whitney U test; $P < 0.0001$); a. u. indicates arbitrary units. (C) Stacked bar graphs and telomere intensity maps from representative fields of X1 FACS sorted cells of 7dS and 20dS planarians displayed in B. The maps display the nuclei coloured according to their telomere fluorescence intensity (four categories of intensity). The stacked bar graphs represent the proportion of nuclei within a given category of intensity. X1 at 7dS is chosen as the reference condition and set up to allocate in each range of intensity or category approximately one fourth of the total cells. X1 at 20dS shows a higher percentage of stem cells with long telomeres and a lower percentage of stem cells with short telomeres than X1 at 7dS. Magenta squares indicate the area of magnification displayed next to the main images. a.u. indicates arbitrary units. Scale bars indicate 250 µm in the main images and 30 µm in the high magnification images. (D) The schematic displays the process of starvation of planarians that had the same size at 7dS and 30dS. (E) Column scatter plot showing all the cells pooled from a total of 2 planarians per condition. The median telomere intensity is higher in 30dS stem cells than in 7dS (two-tailed Mann-Whitney U test; $P < 0.0001$). n indicates the number of planarians analyzed. (F) Telomere intensity maps and stacked bar graphs for representative tissue sections from E. The intensity maps display the nuclei coloured according to their telomere fluorescence intensity (four categories of intensity). The stacked bar graphs represent the proportion of nuclei within a given category of intensity. 7dS is chosen as the reference condition and set up to allocate in each range of intensity or category approximately one fourth of the total cells. 30dS show a higher percentage of stem cells with high telomere intensity. a.u. indicates arbitrary units. Scale bars indicate 1 mm. Anterior is to the left and dorsal is up.

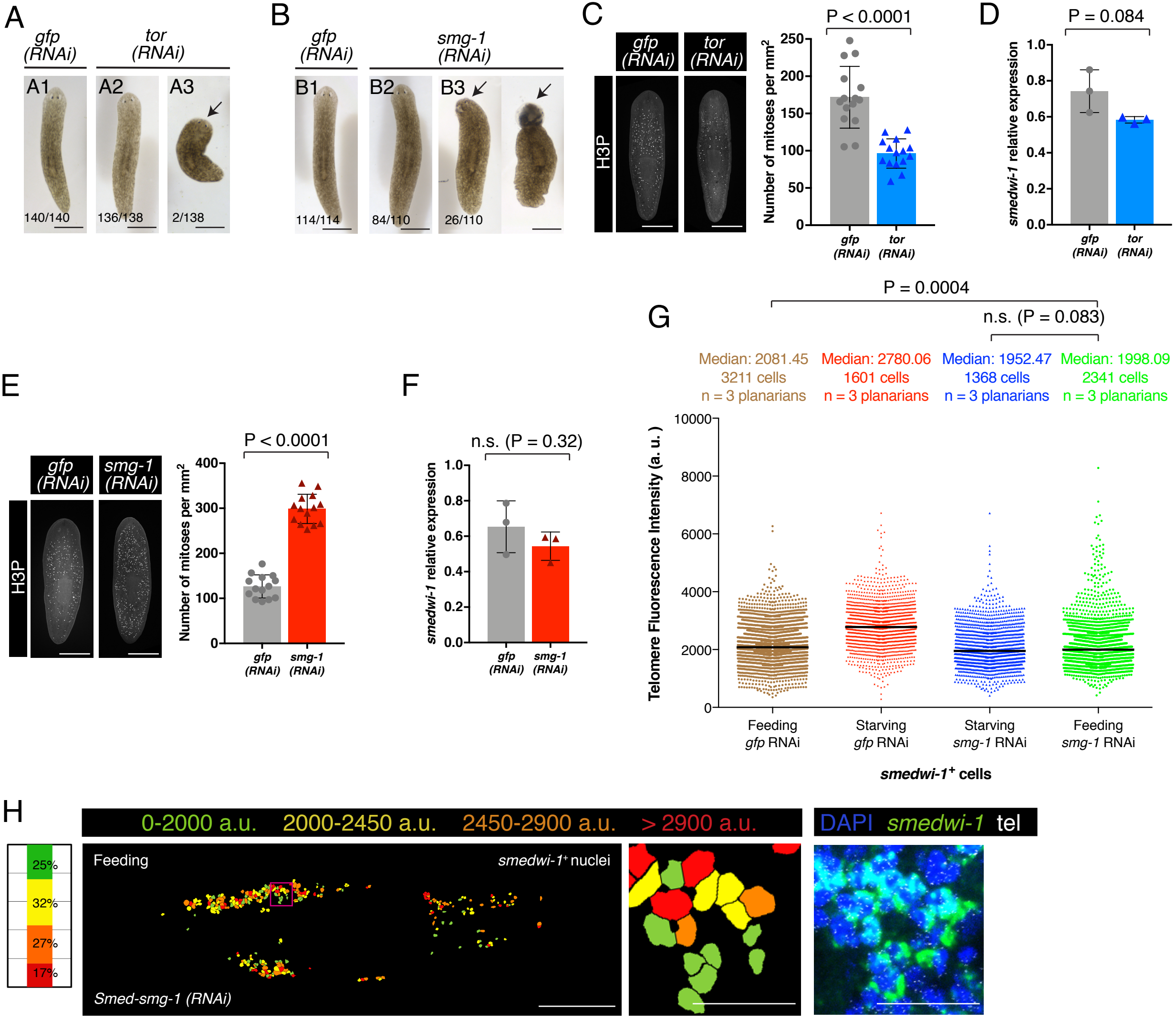

A
gfp (RNAi)
tor (RNAi)
A1
A2
A3
140/140
136/138
2/138
B
gfp (RNAi)
smg-1 (RNAi)
B1
B2
B3
114/114
84/110
26/110
C
gfp (RNAi)
tor (RNAi)
H3P
P < 0.0001
Number of mitoses per mm²
D
P = 0.084
smedwi-1 relative expression
E
gfp (RNAi)
smg-1 (RNAi)
H3P
P < 0.0001
Number of mitoses per mm²
F
n.s. (P = 0.32)
smedwi-1 relative expression
G
P = 0.0004
n.s. (P = 0.083)
Median: 2081.45
3211 cells
n = 3 planarians
Median: 2780.06
1601 cells
n = 3 planarians
Median: 1952.47
1368 cells
n = 3 planarians
Median: 1998.09
2341 cells
n = 3 planarians
Telomere Fluorescence Intensity (a. u.)
Feeding gfp RNAi
Starving gfp RNAi
Starving smg-1 RNAi
Feeding smg-1 RNAi
smedwi-1⁺ cells
H
0-2000 a.u.
2000-2450 a.u.
2450-2900 a.u.
> 2900 a.u.
25%
32%
27%
17%
Feeding
smedwi-1⁺ nuclei
Smed-smg-1 (RNAi)
DAPI
smedwi-1
tel

Figure S5. **Controls for *Smed-tor* and *Smed-smg-1* RNAi experiments. Related to Figure 6.** (A) Representative live images of *gfp(RNAi)* and *tor(RNAi)* planarians taken before fixation. Most of the *tor(RNAi)* planarians show no morphological phenotype at the time point chosen for fixation (day 42) (A2). Only A1 and A2 planarians were fixed for telomere length quantification. The arrow in A3 indicates head regression. Scale bars indicate 500 µm. (B) Representative live images of *gfp(RNAi)* and *smg-1(RNAi)* planarians taken before fixation. Most of the *smg-1(RNAi)* planarians show no morphological phenotype at the time point chosen for fixation (day 24) (B2). Only B1 and B2 planarians were fixed for telomere length quantification. The arrows in B3 indicate different degrees of tumour formation. Scale bars indicate 500 µm. (C) Representative images of *gfp(RNAi)* and *tor(RNAi)* planarians stained for anti-Histone H3 phosphorylated (anti-H3P) which labels mitotic stem cells at the time point chosen for fixation. The graph shows that *tor(RNAi)* planarians have lower number of mitoses than *gfp(RNAi)* animals (two-tailed Student's t-test with equal sample variance, $P < 0.0001$; $n = 15$ planarians per condition); the error bars indicate deviation from the mean; scale bars indicate 500 µm (D) Real time PCR shows that *smedwi-1* relative expression respect to the gene control is slightly lower in *tor(RNAi)* planarians than in the *gfp* (two-tailed Student's t-test with equal sample variance, $P = 0.084$; 3 biological replicates per condition) at the time point chosen for fixation; the error bars indicate deviation from the mean. (E) Representative images of *gfp(RNAi)* and *smg-1(RNAi)* planarians stained for anti-Histone H3 phosphorylated (anti-H3P) which labels mitotic stem cells at the time point chosen for fixation. The graph shows that *smg-1(RNAi)* planarians have higher number of mitoses than *gfp* animals (two-tailed Student's t-test with equal sample variance, $P < 0.0001$; $n = 14$ planarians per condition); the error bars indicate deviation from the mean; scale bars indicate 500 µm (F) Real time PCR shows that *smedwi-1* relative expression respect to the gene control is not significantly different in *smg-1* RNAi when compared to *gfp* controls (two-tailed Student's t-test with equal sample variance, $P = 0.32$; 3 biological replicates per condition) at the time point chosen for fixation; the error bars indicate deviation from the mean. (G) Column scatter plot shown in Figure 6F with an extra column that represents the "feeding *Smed-smg-1(RNAi)*" stem cell population. It shows all the cells pooled from a total of 3 planarians. The median telomere intensity is slightly lower in *Smed-smg-1(RNAi)* (feeding conditions) than in *gfp(RNAi)* (feeding conditions) stem cells (two-tailed Mann-Whitney U test; $P = 0.0004$). There are not significant differences between *Smed-smg-1(RNAi)* (feeding conditions) and *Smed-smg-1(RNAi)* (starving conditions) (two-tailed Mann-Whitney U test; $P = 0.083$). n indicates the number of planarians analyzed. (H) Stacked bar graph and telomere intensity map from a representative tissue section of *Smed-smg-1(RNAi)* (feeding conditions) planarians from G. The map displays the nuclei coloured according to their telomere fluorescence intensity (four categories of intensity). The stacked bar graphs represent the proportion of nuclei within a given category of intensity. *gfp(RNAi)* (feeding conditions) (Figure 6H) is chosen as the reference condition and set up to allocate in each range of intensity or category approximately one fourth of the total cells. Similar proportions are shown for *gfp(RNAi)* (feeding conditions) (Figure 6H) and *Smed-smg-1(RNAi)* (feeding conditions) cell populations. Magenta squares indicate the area of magnification displayed next to the main images. a.u. indicates arbitrary units. Scale bars indicate 200 µm in the main images and 30 µm in the high magnification images.

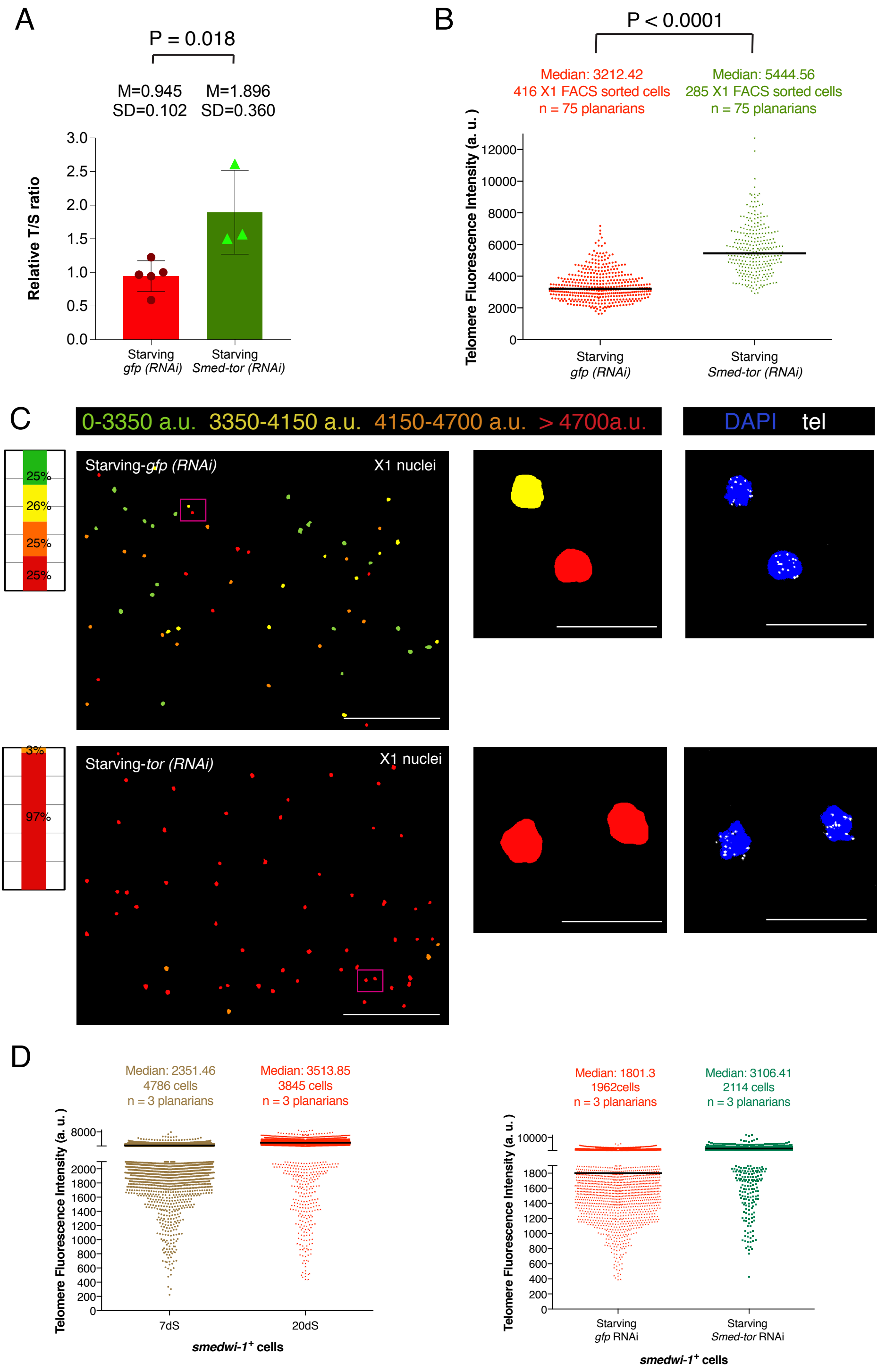

A
P = 0.018
M=0.945
SD=0.102
M=1.896
SD=0.360
Relative T/S ratio
3.0
2.5
2.0
1.5
1.0
0.5
0.0
Starving gfp (RNAi)
Starving Smed-tor (RNAi)
B
P < 0.0001
Median: 3212.42
416 X1 FACS sorted cells
n = 75 planarians
Median: 5444.56
285 X1 FACS sorted cells
n = 75 planarians
Telomere Fluorescence Intensity (a. u.)
12000
10000
8000
6000
4000
2000
0
Starving gfp (RNAi)
Starving Smed-tor (RNAi)
C
0-3350 a.u. 3350-4150 a.u. 4150-4700 a.u. > 4700a.u.
DAPI tel
25%
26%
25%
25%
Starving-gfp (RNAi)
X1 nuclei
3%
97%
Starving-tor (RNAi)
X1 nuclei
D
Median: 2351.46
4786 cells
n = 3 planarians
Median: 3513.85
3845 cells
n = 3 planarians
Telomere Fluorescence Intensity (a. u.)
8000
2000
1800
1600
1400
1200
1000
800
600
400
200
0
7dS
20dS
smedwi-1+ cells
Median: 1801.3
1962cells
n = 3 planarians
Median: 3106.41
2114 cells
n = 3 planarians
Telomere Fluorescence Intensity (a. u.)
10000
1800
1600
1400
1200
1000
800
600
400
200
0
Starving gfp RNAi
Starving Smed-tor RNAi
smedwi-1+ cells

Figure S6. *Smed-tor* RNAi further increases the effect of starvation on stem cell telomere length. Related to Figure 6. (A) Telomere quantitative PCR (qPCR) comparing *gfp(RNAi)* and *tor(RNAi)* whole planarians. qPCR was performed on genomic DNA for at least 3 biological replicates (5 planarians per replicate) per condition. The qPCR determines the ratio of telomere (T) repeat copy number to a single-copy (S) gene (genomic DNA from dd_Smed_v6_2426_0_1) copy number (T/S ratio) compared with a reference DNA sample *gfp* RNAi. The graph shows that *tor(RNAi)* planarians have a higher T/S ratio than *gfp(RNAi)* (two-tailed Student's t-test with equal sample variance, $P < 0.05$) and thus higher telomere length. M, mean; SD, standard deviation. (B) Column scatter plot showing X1 FACS sorted cells that come from *gfp* RNAi and *tor* RNAi (75 planarians per condition were used for the FACS). The median telomere intensity is higher in stem cells from *tor* RNAi than from *gfp* control planarians (two-tailed Mann-Whitney U test; $P < 0.0001$); a. u. indicates arbitrary units. (C) Stacked bar graphs and telomere intensity maps for representative fields of X1 FACS sorted cells from starving *gfp(RNAi)* and *tor(RNAi)* planarians. The maps display the nuclei coloured according to their telomere fluorescence intensity (four categories of intensity). The stacked bar graphs represent the proportion of nuclei within a given category of intensity. *gfp* RNAi is chosen as the reference condition and set up to allocate in each range of intensity or category approximately one fourth of the total cells. X1 cells from *tor(RNAi)* planarians show a higher percentage of stem cells with long telomeres and a lower percentage of stem cells with short telomeres than *gfp* control planarians. Magenta squares indicate the area of magnification displayed next to the main images. a.u. indicates arbitrary units. Scale bars indicate 250 µm in the main images and 30 µm in the high magnification images. (D) Column scatter plots showing the data from Figure 5C and Figure 6E respectively focusing on the stem cells with low telomere intensity.

Supplemental Experimental Procedures

Starvation experiments

Planarians were starved as stated in the text. In experiments displayed in Figure S4D-F, planarians at 7dS and 30dS had both the same area 4 mm^2 (5-5.5 mm length at 7dS and 5.5- 6mm length at 30dS). Graph paper placed under the Petri dish was used to pre-select animals of the same length and the final selection was done after measuring the areas with the Leica Application Suite (Leica) on photographs of live planarians taken under a stereomicrospe coupled with a Leica camera MC170 HD (Leica). For the rest of experiments planarians were 4-5.5 mm length at 7dS when starting the experiments and size selection was done on live animals by using graph paper.

RNAi experiments

At least 15 planarians per experiment were left to follow and corroborate the corresponding phenotypes. Planarians under feeding conditions were fed once a week, while planarians under starving conditions were left under starvation during the whole experiment. *tor* RNAi experiments could not be performed under feeding conditions since they are not able to maintain the pharynx and thus they cannot eat (Gonzalez-Estevez et al., 2012b).

Fixation, paraffin embedding and sectioning

Fixation of the planarians was performed by killing the animals at room temperature (RT) in 2% HCl diluted in 5/8 concentration of Holtfreter's standard saline solution for 5 minutes (min) followed by 2 washes of 5 min in Holtfreter 5/8 and fixation in 4% paraformaldehyde (16% Paraformaldehyde aqueous solution; Electron Microscopy Sciences) diluted in Holtfreter 5/8 for 4 hours at 4°C. After an overnight (ON) wash in Holtfreter 5/8, planarians were dehydrated through a series of ethanol washes diluted in RNase free miliQ water: 70% ethanol, 80% ethanol, 96% ethanol and 2 x 100% ethanol, 10 minutes each and a last step with 3 x xylene, 4 min each. The embedding of the samples was then performed either manually or with an Excelsior AS Tissue processor (Thermo Fisher Scientific). 7 μm sagittal sections were obtained for all the planarians and placed on Superfrost Plus slides (Menzel-Gläser; Thermo Fisher Scientific).

In situ hybridization on planarian sections

We generated templates with T7 promoters appended to the sense strand and hapten-labelled RNA probes were synthesized by using an *in vitro* labelling kit (Roche, Basel, Switzerland). The following oligos were used to obtain the templates:

smedwi-1F: 5'- ggccgcggAAGTGGTGGTATTCGAGAAGGA-3';

smedwi-1R: 5'- gccccggcCACGAATCGTAATCGGTTGTTCT-3';

Smed-nosF: 5'- ggccgcggCCTGAATCATTGAAGATGGCTA-3'

Smed-nosR: 5'- gccccggcCCAAGAGTGGATTGTGACATGCT-3'

Smed-prog1F: 5'-ggccgcggGTGATTGCGTTCGCGTATATT-3'

Smed-prog1R: 5'-gccccggcCATTTATCCAGCGCGTCATATTC-3'

Smed-Agat1F: 5'-ggccgcggGAAATGATTGAGTCCACCATGA-3'

Smed-Agat1R: 5'-gccccggcCTGCAATATCTGGATAAGGAGCA-3'

Smed-tgs-1F: 5'-ggccgcggAAAACGCAATCAAAGCAACTGA 3'

Smed-tgs-1R: 5'-gccccggcCAGTGAGAATGACGGATTCCTG 3'

Deparaffinization and rehydration of slide-mounted sections was performed either manually or with a Leica Autostainer XL (Leica Microsystems) through a series of ethanol washes diluted in RNase free miliQ water: 3 x xylene, 2 x 100% ethanol, 95% ethanol, 70% ethanol, 3 min each. In single fluorescent *in situ* hybridizations (FISH) permeabilization was performed in 10mM pH 6.0 citrate buffer by cooking the slides for 3 min in a pressure cooker. 1/100 anti-FITC-POD was used and tyramide signal amplification was performed by diluting the TSA plus reagent 1/50 in amplification buffer (TSA Plus Fluorescein or Cy5; Perkin Elmer) and incubating the slides for 10-20 min. For double FISH, the first POD was inactivated by incubating the slides in 2% H_2O_2 in PBST for one hour at RT followed by 6 washes of 10 min each in PBST and then 6 washes of 10 min each in Maleic buffer previous to apply the blocking solution. 1/100 anti-DIG-POD was used and tyramide signal amplification was performed by diluting the TSA plus reagent 1/50 in amplification buffer (TSA Plus Cy5; Perkin Elmer) and incubating the slides for 10-20 min. Nuclei counterstaining was performed by incubating the slides for 15 min in 5μg/ml DAPI.

Telomere Quantitative Fluorescent *in situ* hybridization (TelQ-FISH)

TelQ-FISH protocol on planarian paraffin sections started after either the FISH or the immunohistochemistry in the PBS washes that followed the DAPI staining. TelQ-FISH on FACS sorted cells started immediately after drying the slides. Slides (with paraffin sections or FACS sorted cells) were then fixed in 4% formalin in PBS for 2 min at RT, followed by 3 x PBS washes, 5 min each and a treatment with acidic pepsin for 10 min at 37°C. After washing 2 times for 5 min each in PBS, another 2 min fixation step with 4% formalin was performed and 2 x 5 min PBS washes. Then the slides went through a series of ethanol washes to dehydrate the tissue: 5 min ethanol 70%, 5 min ethanol 90% and 5 min ethanol 100%. Slides were air dried on the bench at RT for about 20 min or until totally dried. Then hybridization solution was added to each slide covered with a coverslip (10mM Tris pH 7.2, 8.56% Magnesium Buffer, 70% Deionized formamide, 0.25% Blocking reagent and 0.5 μg/ml Telomere CCCTAA PNA probe cy3-conjugated either from Applied Biosystems or from Panagene (TelC-Cy3 probe; since it has been shown that the planarian *S. mediterranea*, as other Platyhelminthes, have the same telomere repeat as vertebrates at their chromosome ends (Bombarova et al., 2009; Joffee, 1996) we used the vertebrate-specific telomere fluorescent peptide nucleic acid (PNA) probe). Tissue was denatured by placing the slides on a thermoblock at 80 °C for 3 min. Hybridization was then performed in a humid chamber in the dark for 2 hours at RT. Slides were washed 2 times for 15 min each with washing solution (70% formamide, 10mM Tris pH 7.2, 0.1% BSA) and then 3 times in PBST, 5 min each. The final step was an extra DAPI stain for 10 min. Slides were mounted in Vectashield (Vector Laboratories, Burlingame, California).

Telomere length image acquisition and quantification

Stacks (1 μm step size) for all the channels from equivalent tissue sections or FACS sorted cells were acquired either with a Leica TCS SP5 confocal microscope (Leica Microsystems; 16 bits images; 63x lens) or a Zeiss ApoTome.2 equipped with a Zeiss Axiocam 503 mono (Carl Zeiss, Jena; 14 bits images; 40x lens). Automatized high resolution z-stack imaging of several whole sections was possible by using the Matrix Screener Wizard from Leica at the Leica TCS SP5 confocal microscope or tile scan in the Zeiss ApoTome.2. Either the 561 nm DPSS laser was always maintained constant through all the slides from one same FISH or the exposition time in the case of the ApoTome. Cy3 samples with stronger signals from one same TelQ-FISH were used to set the intensity for the whole experimental scan to avoid having over-exposed signals. After automatically stitching all the tiles of each mosaic stack, a maximum projection (MP) for each channel was generated. In order to generate the binary masks containing nuclear areas based on the DAPI channel, the MP from the DAPI channel and the fluorescein/Cy5 channel needed first some processing by using a combination of Photoshop CS6 or CC (Abobe Systems Incorporated) and Fiji (Schindelin et al., 2012). Briefly, a Gaussian blur filter was applied to the DAPI MP to reduce the image noise or detail. Then the image was thresholded, transformed into a 1-bit binary image and watershed segmented to generate the DAPI mask. A Gaussian blur filter was also applied to the fluorescein/Cy5 image. A custom-made plugin (available upon request) for Fiji (Schindelin et al., 2012) was used to generate the binary mask for those nuclei positive for the fluorescein/Cy5 channels. In order to use the plugin, the fluorescein/Cy5 image was high percentage thresholded to obtain an image where the signal was kept at a minimum (few pixels per cell) and transformed into a 1-bit binary. The plugin generated a new mask (e.g., *smedwi-1* or *Smed-nanos* mask), with only those nuclei from the DAPI mask, which were positive for the specific markers. *smedwi-1*$^-$ masks were generated by substracting the *smedwi-1*$^+$ mask to the DAPI mask and using the arithmetic function "substract" in MetaMorph (version 6.3r6, Molecular Devices). By creating a multilayered image in Photoshop formed by the DAPI MP image, the DAPI mask, the telomere MP image, the fluorescein/Cy5 MP image and for instance *smedwi-1*$^+$, *Smed-nanos*$^+$ or *smedwi-1*$^-$ masks and changing the transparency of the layers as required, it was possible to improve the masks by manually fine segment those nuclei which were very close to each other (i. e. the brain cells and the pharynx cells), remove artifacts which were added by the plugin and to improve all the masks ensuring that all telomeres fell inside their nuclear area. The Cy3 image (telomeres) was only processed to remove general noise across the entire tissue section by applying the Detect Peaks feature from NIS-Elements software (Nikon Instruments) and left as originally taken at 16 or 14 bits ready for quantification.

Quantification was performed using the MetaMorph software (version 6.3r6, Molecular Devices). Briefly, 1-grey value was added to the original Cy3 image and then it was combined with a binarized DAPI mask through "logical and" arithmetic function. Then it was thresholded and Cy3 fluorescence intensity was measured as "average gray value" units (the total intensity per nucleus divided by the nucleus area) using the Integrated Morphometry Analysis module and shown as arbitrary units of fluorescence. Intensity values were exported to Excel for further analysis and to generate the cumulative frequency graphs and stacked bar charts. GraphPad Prism 7.0d was used to generate the column scatter plots and the frequency histograms. Arbitrary units of fluorescence cannot be compared between different scatter plots because differences in the protocols (e.g., single FISH versus FISH followed by immunohistochemistry), PNA aliquots and/or experimental design (e.g., wild type planarians versus GFP injected) lead to differences in the overall fluorescent intensity of the whole TelQ-FISH. All data obtained in the ApoTome (Figure 3 and Figure 4) was multiplied per 20 to bring the values into line with the ones obtained in the Confocal. The Configure Object Classifier module was used to classify and generate the telomere intensity maps colored according to their telomeric intensity.

Quantification of the number of telomeres per cell and intensity of the different telomeres in a cell

The number of telomeres per cell was calculated using Fiji (Schindelin et al., 2012). Each local maxima (telomere) was transformed into a single black pixel using the "find maxima" command. Then Regions of interest (ROI) were created based on nuclear DAPI staining and the "measure" command was then used to calculate "RawIntDen" (the sum of the values of the pixels in each ROI) which, divided by 255 (total black) results in the number of telomeres per nuclei. For measuring intensity per telomere an individual telomere ROI set for each nucleus was created and the sum of pixel-intensity (RawIntDen) for each ROI (telomere) measured using Fiji (Schindelin et al., 2012).

Whole-mount immunohistochemistry

Whole-mount immunohistochemistry was carried out as published elsewhere (Cebria and Newmark, 2005). An anti-Histone H3 phosphorylated at serine 10 (diluted 1/ 500; Santa Cruz, sc-8656-R) was used to detect mitotic stem cells. Whole planarian optical sections were obtained using a Zeiss AXIO Zoom.V16 (ApoTome.2) equipped with an Axiocam 506 camera (Carl Zeiss, Jena). Quantifications were done using the Object Counter 3D plugin from Fiji (Schindelin et al., 2012).

Real-time PCR

Real time PCR was performed as previously described (Gonzalez-Estevez et al., 2012a). The transcript with ID 5685 from Dresden transcriptome (PlanMine) (Rozanski et al., 2019) was used as internal control. 3 biological replicates (5 planarians per replicate) were used per sample. Each biological replicate was replicated three times. PCR reactions with performed using iTaq Universal SYBER® Green Supermix (BIO-RAD). Reactions were aliquoted using a QiAgility robot (Qiagen) and analyzed with a 7500 Real Time PCR System (Applied Biosystems).

Flurorescence-activated cell sorting (FACS)

Planarian dissociation and FACS were performed as described before (Hayashi et al., 2006), using BD FACSAria III. For TelQ-FISH, drops of 5 μl of sorted cells (subpopulation X1) at a concentration of 1000 cells/ml were placed on poly-lysine-coated slides and dried for 5 min at 37ºC. X1 subpopulation contains dividing stem cells since it disappears after lethal doses of gamma-irradiation (Hayashi et al., 2006).

Telomere quantitative PCR (telomere qPCR)

Genomic DNA extraction from 5 whole planarians per biological replicate was performed as previously described (Tan et al., 2012). Relative telomere length (TL) was measured by using an optimized version of the quantitative PCR method previously described (Cawthon, 2002). It determines the ratio of telomere (T) repeat copy number to a single-copy (S) gene copy number (called T/S ratio) in experimental samples as compared with a reference DNA sample. The single-copy gene used was the corresponding genomic sequence of transcript dd_Smed_v6_2426_0_1 (Smes_g4_19:2186986..2189312) in PlanMine (Rozanski et al., 2019). Telomere qPCRs and single-copy gene qPCRs were performed in separate wells using the following primers: TelF (5′-CGG TTT GTT TGG GTT TGG GTT TGG GTT TGG GTT TGG GTT-3′) and TelR (5′-GGC TTG CCT TAC CCT TAC CCT TAC CCT TAC CCT TAC CCT-3′) (O'Callaghan et al., 2008) used at a final concentration of 900 nM each; 2426F (5′-GTT GCT GGG CCA ATT AGG CG-3′) and 2426R (5′-CAA TTC GCT CTC TGA TCC GC-3′) used at final concentration of 300nM each. Each 10μl amplification reaction volume contained 1x Power SYBR Green PCR Master mix (Applied Biosystems) and 3 ng of genomic DNA samples. One of the replicates of 7dS (Figure S4A) and one of the replicates of *gfp (RNAi)* (Figure S6A) were used as reference DNA samples. Tubes containing 27, 9, 3, 1 and 0.333 ng of the reference DNA were included in each run to allow the quantification of the samples relative to the reference DNA by the standard curve method. Each biological replicate was run in triplicates. At least 3 biological replicates per condition were analysed. qPCRs were carried out on The StepOne Plus Real-Time PCR System (Applied Biosystems). PCR cycling conditions for the telomere amplification were 95°C for 10 min, followed by 40 cycles of 95°C for 15 sec, 58°C for 30 sec and 72 °C for 30 sec, and for the 2426 gene the cycling conditions were 95°C for 10 min, followed by 40 cycles of 95°C for 15 sec and 65°C for 1 min. Melting curve analysis was carried out at the end of each PCR experiment.

References

Bombarova, M., Vitkova, M., Spakulova, M., and Koubkova, B. (2009). Telomere analysis of platyhelminths and acanthocephalans by FISH and Southern hybridization. Genome *52*, 897-903.

Cawthon, R.M. (2002). Telomere measurement by quantitative PCR. Nucleic Acids Res *30*, e47.

Cebria, F., and Newmark, P.A. (2005). Planarian homologs of netrin and netrin receptor are required for proper regeneration of the central nervous system and the maintenance of nervous system architecture. Development *132*, 3691-3703.

Gonzalez-Estevez, C., Felix, D.A., Rodriguez-Esteban, G., and Aboobaker, A.A. (2012a). Decreased neoblast progeny and increased cell death during starvation-induced planarian degrowth. Int J Dev Biol *56*, 83-91.

Gonzalez-Estevez, C., Felix, D.A., Smith, M.D., Paps, J., Morley, S.J., James, V., Sharp, T.V., and Aboobaker, A.A. (2012b). SMG-1 and mTORC1 act antagonistically to regulate response to injury and growth in planarians. PLoS Genet *8*, e1002619.

Hayashi, T., Asami, M., Higuchi, S., Shibata, N., and Agata, K. (2006). Isolation of planarian X-ray-sensitive stem cells by fluorescence-activated cell sorting. Dev Growth Differ *48*, 371-380.

Joffee, B.S.I., Macgregor HC (1996). Ends of Chromosomes in Polycelis tenuis (Platyhelminthes) have telomere repeat TTAGGG. Chormosome Research *4*, 323-324.

O'Callaghan, N., Dhillon, V., Thomas, P., and Fenech, M. (2008). A quantitative real-time PCR method for absolute telomere length. BioTechniques *44*, 807-809.

Rozanski, A., Moon, H., Brandl, H., Martin-Duran, J.M., Grohme, M.A., Huttner, K., Bartscherer, K., Henry, I., and Rink, J.C. (2019). PlanMine 3.0-improvements to a mineable resource of flatworm biology and biodiversity. Nucleic Acids Res *47*, D812-D820.

Schindelin, J., Arganda-Carreras, I., Frise, E., Kaynig, V., Longair, M., Pietzsch, T., Preibisch, S., Rueden, C., Saalfeld, S., Schmid, B.*, et al.* (2012). Fiji: an open-source platform for biological-image analysis. Nat Methods *9*, 676-682.

Tan, T.C., Rahman, R., Jaber-Hijazi, F., Felix, D.A., Chen, C., Louis, E.J., and Aboobaker, A. (2012). Telomere maintenance and telomerase activity are differentially regulated in asexual and sexual worms. Proc Natl Acad Sci U S A *109*, 4209-4214.

Video S1. Tissue section stained for telomeres.
The video shows a zoom into a tissue section stained for telomeres with increased exposure in order to be able to observe all the telomeres from all the cells in all the planarian tissues.

Video S2. The highest telomere intensity in a given tissue section can be easily observed.
The video shows a zoom into the dorsal part of a tissue section. The brightest telomeres can be easily seen (arrows).
