# Supplementary material for: Downregulation of mTOR Signaling Increases Stem Cell Population Telomere Length during Starvation of Immortal Planarians
Source: Stem Cell Reports. 2019 Jul 25;13(2):405–18. doi: 10.1016/j.stemcr.2019.06.005 (PMC6700675; doi:10.1016/j.stemcr.2019.06.005)
Supplement: Document S2. Article plus Supplemental Information [file mmc4.pdf]

Stem Cell Reports
Article
OPEN ACCESS

Downregulation of mTOR Signaling Increases Stem Cell Population Telomere Length during Starvation of Immortal Planarians

Marta Iglesias,[1,2,6] Daniel A. Felix,[1] Óscar Gutiérrez-Gutiérrez,[1] Maria del Mar De Miguel-Bonet,[2] Sounak Sahu,[3] Beatriz Fernández-Varas,[4] Rosario Perona,[4,5] A. Aziz Aboobaker,[3] Ignacio Flores,[2,7,*] and Cristina González-Estévez[1,2,7,*]

[1]Leibniz Institute on Aging-Fritz Lipmann Institute (FLI), Beutenbergstrasse 11, 07745 Jena, Germany
[2]Centro Nacional de Investigaciones Cardiovasculares Carlos III (CNIC), Melchor Fernández Almagro 3, 28029 Madrid, Spain
[3]Department of Zoology, University of Oxford, South Parks Road, Oxford OX1 3PS, UK
[4]Instituto de Investigaciones Biomédicas CSIC/UAM, IDiPaz, Arturo Duperier 4, 28029 Madrid, Spain
[5]Ciber Network on Rare Diseases (CIBERER), C/ Alvaro de Bazan, 10, 46010 Valencia, Spain
[6]Present address: Sars Center for Marine Molecular Biology, University of Bergen, Thormøhlensgt. 55, 5006 Bergen, Norway
[7]Co-senior author
*Correspondence: iflores@cnic.es (I.F.), cristina.gonzalez@leibniz-fli.de (C.G.-E.)
https://doi.org/10.1016/j.stemcr.2019.06.005

SUMMARY

Reduction of caloric intake delays and prevents age-associated diseases and extends the life span in many organisms. It may be that these benefits are due to positive effects of caloric restriction on stem cell function. We use the planarian model *Schmidtea mediterranea*, an immortal animal that adapts to long periods of starvation by shrinking in size, to investigate the effects of starvation on telomere length. We show that the longest telomeres are a general signature of planarian adult stem cells. We also observe that starvation leads to an enrichment of stem cells with the longest telomeres and that this enrichment is dependent on mTOR signaling. We propose that one important effect of starvation for the rejuvenation of the adult stem cell pool is through increasing the median telomere length in somatic stem cells. Such a mechanism has broad implications for how dietary effects on aging are mediated at the whole-organism level.

INTRODUCTION

Calorie-restricted diets or periods of fasting (referred to as starvation in prokaryotes and invertebrates) extend the life span of many organisms and can also protect against age-related diseases in rodents, monkeys, and humans (Longo and Mattson, 2014). While there is a general consensus that diet is an important modulator of organismal physiology, molecular and physiological mechanisms through which this is mediated remain poorly described.

Many of our tissues are able to grow, respond to injury, and regenerate by relying on tissue-specific populations of adult stem cells. The regulation of self-renewal and differentiation of adult stem cells is therefore important for the function of many organs. It is now known that stem cell functionality decreases during aging contributing to age-associated pathologies and the overall aging process of the organism (Behrens et al., 2014). Diet is also an emerging important regulator of adult stem cell function. For instance, it has been reported that dietary restriction (DR) enhances stem cell functionality in muscle (Cerletti et al., 2012) and intestinal epithelium (Yilmaz et al., 2012) and improves repopulation capacity of hematopoietic stem cells in early mouse aging (Tang et al., 2016). Fasting, for example, increases the number of a subpopulation of crypt cells more primed to respond to repopulation of the intestine upon refeeding, protects mouse intestinal stem cells from lethal doses of chemotherapy (Richmond et al., 2015; Tinkum et al., 2015), and protects germline stem cells and extends reproductive longevity in *Caenorhabditis elegans* (Angelo and Van Gilst, 2009). While these examples demonstrate the effect of diet on stem cells, they do not provide much explanation of the potential mechanisms by which these effects are mediated.

Planarians are known for their astonishing power of full-body regeneration. The source of that power is the large population of adult stem cells or neoblasts in their bodies, and the planarian species *Schmidtea mediterranea* has become a consolidated model for the study of stem cells and regeneration (Aboobaker, 2011; Rink, 2013). The classical marker to label most proliferating planarian adult stem cells is a member of the Argonaute/PIWI protein family, *smedwi-1* (Reddien et al., 2005). We know that the *smedwi-1*-positive population is highly heterogeneous (van Wolfswinkel et al., 2014), with different degrees of potency and lineage commitment (Scimone et al., 2014). We also know that a fraction of *smedwi-1*$^+$ cells are pluripotent (cNeoblasts) (Wagner et al., 2011). However, we still do not know how many pluripotent stem cells (PSCs) there are and where they would be located in the planarian body. It also remains unclear which, if any, specific molecular signatures are present in pluripotent planarian stem cells, including epigenetic signatures (Mihaylova et al., 2018) or changes in genome structural features such as telomere length.

This is an open access article under the CC BY license (http://creativecommons.org/licenses/by/4.0/).

Planarians are able to undergo prolonged starvation and shrink in size (or "degrow"); however, they maintain activity levels and normal physiology, morphological scale, and the same regenerative power as fed (or growing) animals. Growth and degrowth are repeating cycles in the planarian life history since food availability fluctuates in nature (Felix et al., 2019; Pellettieri, 2019). Planarians maintain the adult stem cell population throughout the process of starvation, as cycling cells remain at 25%–30% of the total cell population (Baguñà, 1976; Gonzalez-Estevez et al., 2012a). Recent data suggest that during starvation either the balance between asymmetric and symmetric divisions is skewed toward symmetric divisions, or there is a relative increase in the self-renewing population of stem cells in detriment of differentiation (Gonzalez-Estevez et al., 2012a; Mangel et al., 2016), in a similar way to what is observed in the mammalian intestine (Yilmaz et al., 2012). This stem cell maintenance strategy allows for a rapid response to a more favorable nutritional environment, or a regenerative response to injury (Felix et al., 2019). At the organismal level planarians do not seem to age, as the stem cell pool seems to be able to infinitely replace damaged or old cells and to respond to injury. It is currently unknown how starvation regulates planarian stem cell biology.

One of the hallmarks of aging is telomere attrition (Lopez-Otin et al., 2013). Telomeres are structures that protect chromosomes from DNA degradation and DNA repair mechanisms. Telomerase maintains telomere length and prevents the end-replication problem in those highly proliferative cells where it is expressed, usually germ stem cells and adult somatic stem cells. However the somatic levels of telomerase are usually not enough to prevent aging-related telomere shortening (Flores and Blasco, 2010). Telomere length has been linked to stem cell pluripotency. Both activation of telomerase and telomere length are known to positively correlate with embryonic stem cell/induced PSC (iPSC) pluripotency, and are also required for somatic cell reprogramming to generate functional iPSCs (Huang et al., 2011; Pucci et al., 2013; Schneider et al., 2013). Telomere quantitative fluorescence *in situ* hybridization (TelQ-FISH or telomapping) applied to diverse mice and plant tissues known to contain stem cell niches has generated topographic telomere length maps showing gradients of telomere length, with the longest telomeres marking the adult stem compartment and the shortest telomeres in the more differentiated compartments within a given tissue (Aida et al., 2008; Flores et al., 2008; Garcia-Lavandeira et al., 2009; Gonzalez-Garcia et al., 2015).

In this work we perform telomere length quantification to identify different populations of cells according to telomere length and the population of stem cells with the longest telomeres (which could potentially be the PSCs) and to check whether or not starvation has a positive effect on the telomere length of stem cells. We adapted the TelQ-FISH technology to *S. mediterranea* paraffin tissue sections and cells sorted by FACS (fluorescence-activated cell sorting). By measuring telomere length in cells of the planarian body *in situ* we observed that planarian adult stem cells have longer telomeres than their differentiated progeny. We also find that the *smedwi-1*$^+$ stem cell population is highly heterogeneous for telomere length, correlating with their known heterogeneity with regard to potency and lineage commitment. We uncover a subclass of *smedwi-1*$^+$ cells with the longest telomeres that correspond to the presumptive planarian germ stem cells and co-express the germline marker *Smed-nanos*. During starvation we observe that the pool of stem cells with the longest telomeres is highly enriched and that this enrichment is dependent on inhibition of mTOR (mammalian target of rapamycin) signaling. We show that fasting is able to rejuvenate the stem cell pool in terms of telomere length through downregulation of mTOR signaling. Our data contribute to the understanding of stem cell biology and aging, and highlight the importance of conducting aging research in long-lived animals.

RESULTS

Planarian Stem Cells Display Higher Median Telomere Length Than Their Descendants

We adapted TelQ-FISH or telomapping (Flores et al., 2008; Gonzalez-Suarez et al., 2000; Zijlmans et al., 1997) to asexual *S. mediterranea* paraffin tissue sections and fixed FACS-sorted cells (Figure 1 and Video S1; Experimental Procedures and Supplemental Experimental Procedures). Using TelQ-FISH we were able to detect an average of ~15 telomeres of the 16 telomeres in FACS-sorted cells and ~14 telomeres per cell in tissue sections (Figures S1A–S1C). A higher number than 16 is expected in planarian cells duplicating their DNA (and therefore their telomeres). A lower number than 16 is expected if two or more telomeres cluster together forming telomere foci, a common feature in other species (Molenaar et al., 2003). As previously reported for other organisms (Gilson and Londono-Vallejo, 2007), we also observed length variation of the telomeres within a cell (Figure S1D). As a way to further validate TelQ-FISH in planarians, we performed fluorescent *in situ* hybridization (FISH) for *smedwi-1* as a stem cell marker (Reddien et al., 2005) in paraffin tissue sections after *Smed-tert* RNAi (telomerase reverse transcriptase [TERT] planarian homolog). We observed a decrease in stem cell telomere length of approximately 3% in 5 weeks after *tert* downregulation (Figures S1E–S1G), which is comparable with the reported overall erosion rate of 1% decrease per week of treatment (290 bp per week) (Tan et al., 2012).

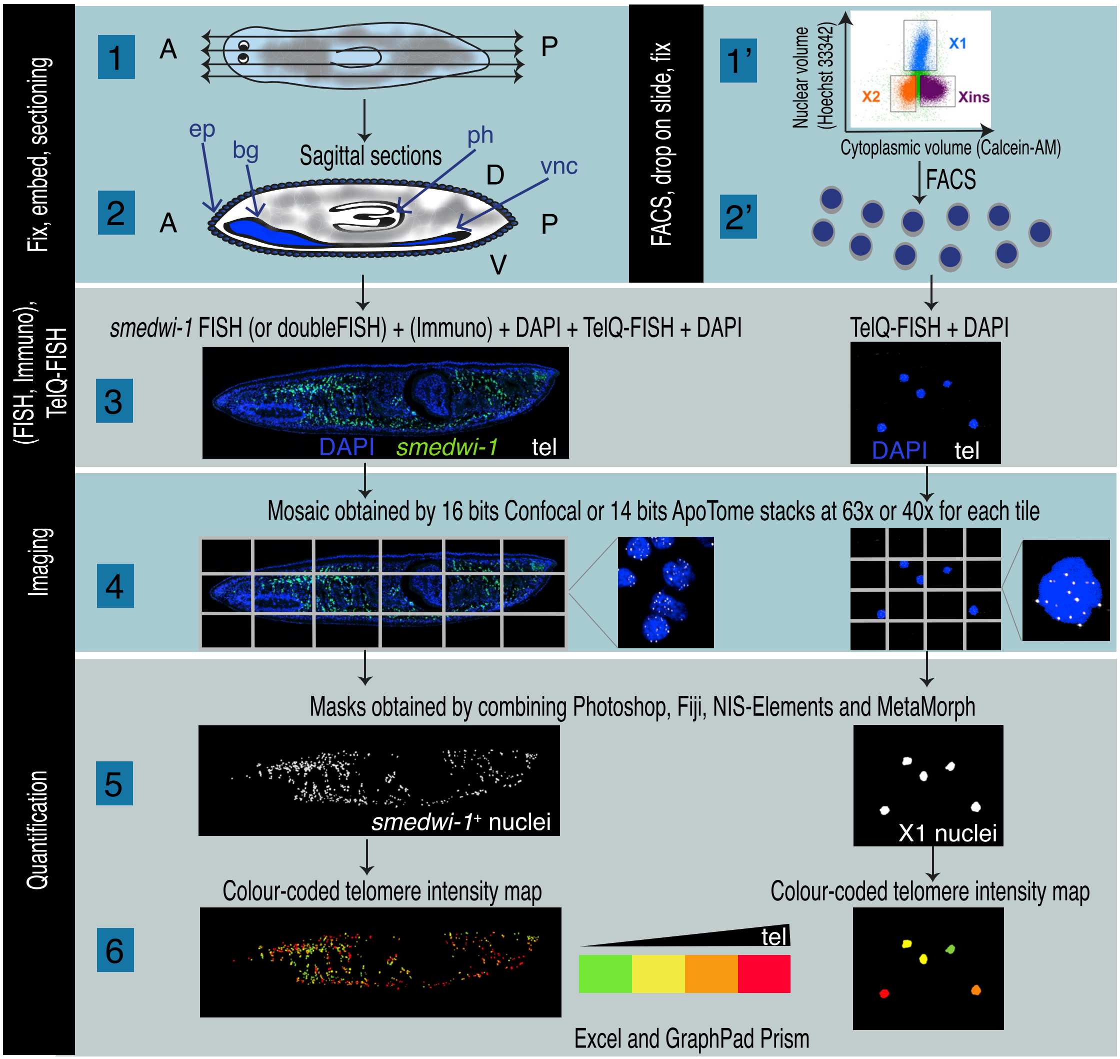

Figure 1. Experimental Flow Diagram

(1 and 2) Fixation, embedding, and sectioning of planarians. Schematic 1 shows the sagittal sectioning planes of paraffin embedded planarians represented by double arrows. The distribution of stem cells in the planarian body is indicated in gray. Schematic 2 represents a sagittal section of the planarian. A, anterior; bg, brain ganglia; D, dorsal; ep, epidermis; ph, pharynx; P, posterior; V, ventral; vnc, ventral nerve cord. (1′ and 2′) Alternatively, planarian cells can be FACS sorted, dried, and fixed on a slide. (3) The next step consists of either single or double FISH followed or not by immunohistochemistry, DAPI staining, and TelQ-FISH or just TelQ-FISH and DAPI staining in case of FACS-sorted cells. The images represent a tissue section after FISH for *smedwi-1* (stem cells in green), TelQ-FISH (telomeres in gray), and DAPI staining (nuclei in blue) and some FACS-sorted cells. (4) Generation of high-resolution images of the telomeres as seen at high magnifications. (5 and 6) Quantification of telomere intensity is done by combining several imaging software packages. Raw data are then exported for further analysis.
See also Figure S1.

However, Tan and colleagues saw a steep initial decline of telomere length, which we did not observe; this may be due to the fact that we analyzed only stem cells while they analyzed whole-planarian genomic DNA, and we used different methods to measure telomere length and possibly had different rates of stress during the RNAi experiment.

We first aimed to elucidate whether we could distinguish planarian stem cells from their progeny specifically by quantifying telomere length. To analyze those different cell populations, we performed FISH on planarian paraffin tissue sections using *smedwi-1* as a stem cell marker. To detect the non-dividing descendants of stem cells, we used two well-established differentiation assays in

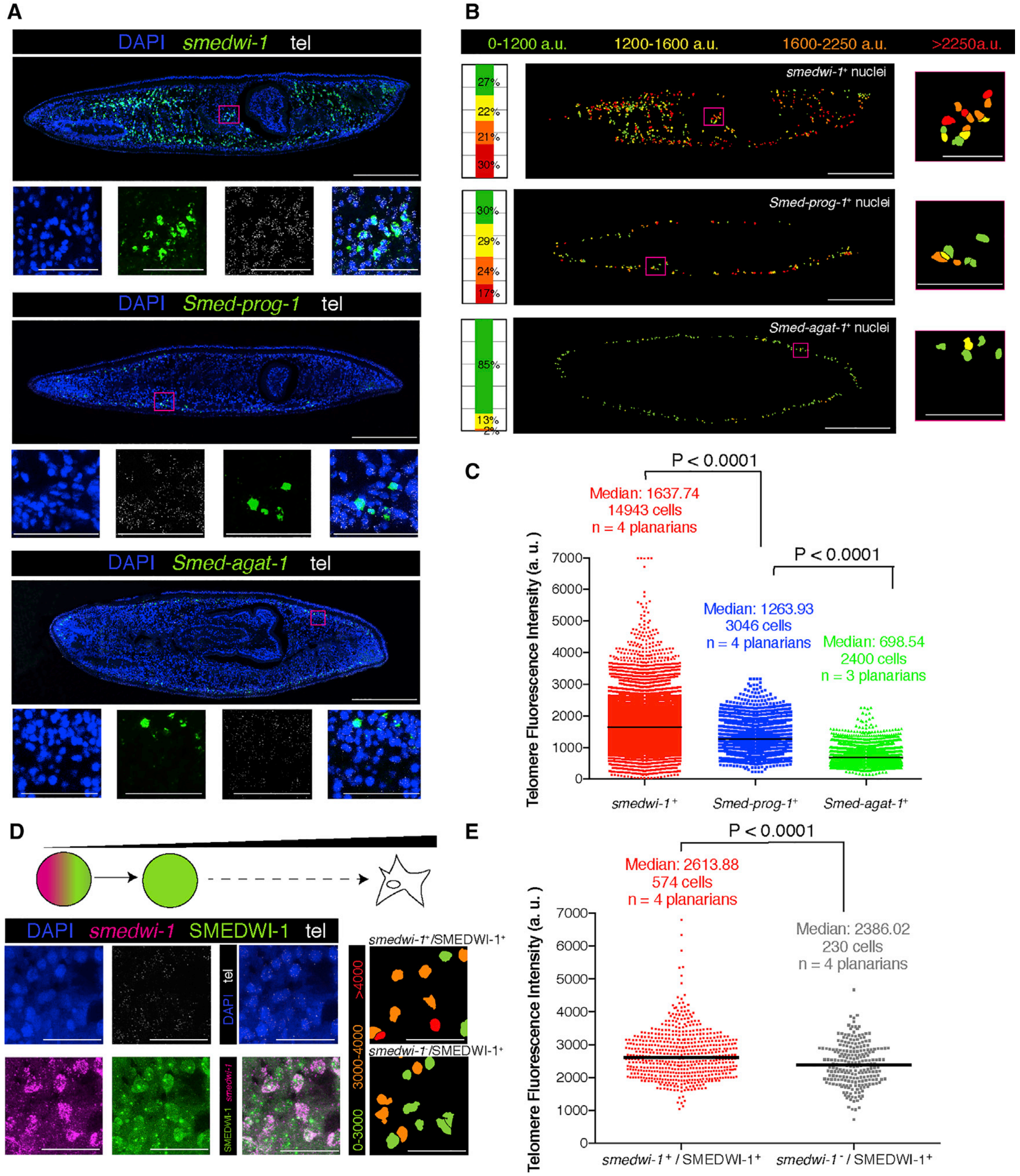

Figure 2. Planarian Stem Cells Display Higher Median Telomere Fluorescence Intensity than Immediate, Early, and Late Post-mitotic Descendant Cells

(A) Maximum confocal projections of representative planarian paraffin sections. Magenta box indicates the region shown below. Anterior is to the left, posterior is to the right, and dorsal is up.

(B) Telomere intensity maps and stacked bar graphs for the representative images in (A) show that stem cells have the longest telomeres when compared with post-mitotic progeny. The intensity maps display the nuclei colored according to their telomere fluorescence intensity. The stacked bar graphs represent the proportion of nuclei within a given category of intensity. *smedwi-1* is chosen as the reference marker and set up to allocate in each range of intensity approximately one-fourth of the total cells. Magenta box indicates the region shown below and corresponds to the high magnifications in (A).

(C) The column scatterplot shows all the pooled nuclei from the total of planarians and cells indicated; the median from *smedwi-1*$^+$ cells is higher than that of *prog-1*$^+$ cells (two-tailed Mann-Whitney U test; $p < 0.0001$), whereas the median from *prog-1*$^+$ cells is higher than that of

(legend continued on next page)

planarians. The first approach consisted in performing FISH for post-mitotic epidermal cell markers (e.g., *Smed-prog-1* and *Smed-agat-1*, which detect early and late post-mitotic descendant cells, respectively) (Eisenhoffer et al., 2008). We observed that the median telomere fluorescence intensity of *smedwi-1*$^+$ cells is the highest when compared with early post-mitotic progeny *prog-1*$^+$ cells and with late post-mitotic progeny *agat-1*$^+$ cells (Figures 2A–2C, S2A, and S2B). At the same time, the median telomere fluorescence of *prog-1*$^+$ cells is higher than that of *agat-1*$^+$ cells (Figures 2B and 2C).

The second approach embraces all stem cell descendants and uses a combination of FISH for *smedwi-1* (mRNA) and immunohistochemistry for SMEDWI-1 (protein) (Guo et al., 2006). SMEDWI-1 (protein) is present in all *smedwi-1* mRNA$^+$ dividing cells but also temporarily detectable in all post-mitotic descendant cells that are *smedwi-1*(mRNA)$^-$ (Wenemoser and Reddien, 2010). Telomere fluorescence intensity quantification in *smedwi-1*$^+$/SMEDWI-1$^+$ cells (stem cells) and in *smedwi-1*$^-$/SMEDWI-1$^+$ cells (very early descendant cells) showed that stem cells display median telomere fluorescence intensity higher than their immediate descendants (Figures 2D, 2E, and S2C–S2E). This supports the results from the other approach and also extends its conclusions from epidermis to all types of descendant cells.

In summary, telomere fluorescence intensity correlates with the degree of differentiation of the cells, with stem cells having the highest median telomere intensity, late post-mitotic descendant cells displaying the lowest, and early post-mitotic or immediate descendants showing intermediate median telomere intensities.

Planarian Germ Stem Cells Are a Subclass of *smedwi-1*$^+$ Cells with the Longest Telomeres

Combined application of telomere length quantification with detection of specific cell markers has been shown to be useful in identifying novel stem cell populations in mice (Garcia-Lavandeira et al., 2009). Indeed, we observed that stem cell distribution of telomere fluorescence intensity shows a higher heterogeneity when compared with early and late post-mitotic progeny (Figure S2A). Similarly, the *smedwi-1*$^+$/SMEDWI-1$^+$ stem cell population shows higher heterogeneity when compared with their immediate progeny *smedwi-1*$^-$/SMEDWI-1$^+$ population (Figure S2C). The data predicts that quantification of telomere length could be a useful technique to localize new subpopulations into the *smedwi-1*$^+$ stem cell pool, such as PSCs.

While performing telomere quantification of *smedwi-1*$^+$ cells, we noticed a subset of cells that had extremely high intensities of telomere fluorescence that stood out under a microscope with low magnification (Video S2). These were scattered cells in the dorsolateral parts of the planarian and distributed along the whole anteroposterior axis. We estimated that those cells represent approximately 3% of the total amount of *smedwi-1*$^+$ cells in the planarian, since taking the top 3% in intensity values of a pool of *smedwi-1*$^+$ cells from three planarians all displayed the dorsolateral distribution along the entire animal (Figure 3). Remarkably, the distribution of those rare cells resembled the expression pattern of the ortholog of the germline marker *nanos* in planarians (Handberg-Thorsager and Salo, 2007; Sato et al., 2006; Wang et al., 2007). Therefore, we hypothesized that the stem cells with the longest telomeres are *nanos*$^+$ cells. To address this, we performed double FISH for *smedwi-1* and *nanos* and subsequently TelQ-FISH on paraffin sections (Figures 4A and 4B). As expected, telomere fluorescence intensity quantification showed that the stem cells positive for both *smedwi-1* and *nanos* have a higher median telomere intensity than the rest of the *smedwi-1*$^+$ cells that are negative for *nanos* (Figure 4C). Of note, if we compare the cells negative for both markers, i.e., the cells in the planarians that are not stem cells, with any of the stem cell groups, we see that any of the three subclasses of stem cells display longer telomeres than non-stem cells (Figures 4B and 4C).

Recently, single-cell RNA sequencing of *smedwi-1*$^+$ cells has uncovered a subpopulation that contains PSCs among other stem cells (Zeng et al., 2018). To investigate its median telomere length we used the marker *Smed-tgs-1*, which

agat-1$^+$ cells (two-tailed Mann-Whitney U test; $p < 0.0001$). The median of *smedwi-1*$^+$ cells is the highest when compared with the other conditions (Kruskal-Wallis test; $p < 0.0001$).
(D) The schematic represents the process of stem cell differentiation. The images show a representative subregion from one of the zones used for quantification. The telomere intensity map displays all the nuclei shown in the representative image colored according to their telomere fluorescence intensity. The population of *smedwi-1*$^+$/SMEDWI-1$^+$ has an increased number of stem cells with strong telomere intensity (either orange or red categories) when compared with its immediate progeny.
(E) The graph shows all the nuclei pooled from all planarians analyzed (four zones per planarian). The median telomere intensity is lower in the SMEDWI-1$^+$/*smedwi-1*$^-$ when compared with SMEDWI-1$^+$/*smedwi-1*$^+$ (two-tailed Mann-Whitney U test; $p < 0.0001$).
Arbitrary units are not comparable between (C) and (E) for being two independent experiments. tel, telomeres; n, number of planarians analyzed; a.u., arbitrary units of fluorescence. Scale bars, 1 mm (A and B, main images), 220 μm (A and B, high-magnification images), and 60 μm (D).
See also Figure S2.

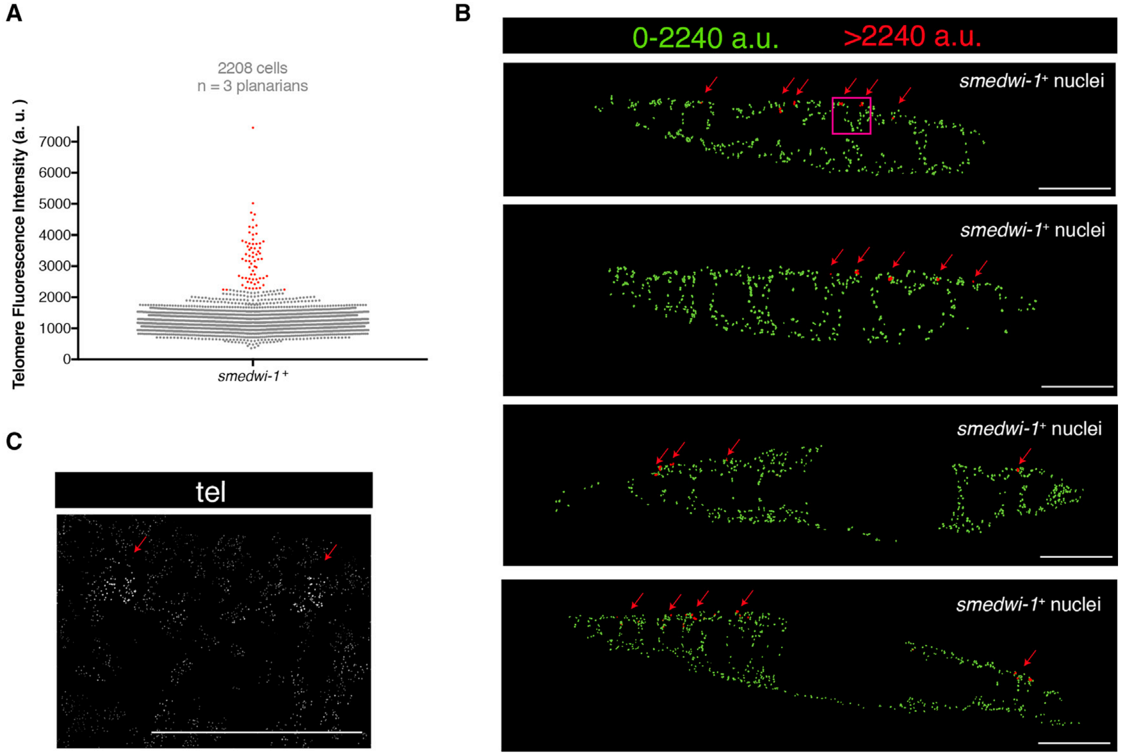

Figure 3. The Top 3% in Telomere Intensity of *smedwi-1*$^+$ Cells Are Distributed Laterodorsally along the Whole Planarian Length

(A) The column scatterplot shows all the pooled *smedwi-1*-positive nuclei. In red are the top 3% of cells in telomere intensity.

(B) All sections quantified in (A) displayed as telomere intensity maps for *smedwi-1*$^+$ stem cells. In red are the stem cells with the longest telomere intensity (top 3% in A; red arrows). Anterior is to the left, dorsal is up. The magenta box indicates the region in (C).

(C) The telomeres with the strongest intensity (red arrows) are easily distinguishable.

Scale bars, 200 μm (main images) and 30 μm (high-magnification images).

defines that subpopulation and represents 25% of the total *smedwi-1*$^+$ stem cells (Zeng et al., 2018) (Figure S3A). Double FISH for *tgs-1* and *smedwi-1* followed by TelQ-FISH showed no significant differences between the *smedwi-1*$^+$/*tgs-1*$^+$ and *smedwi-1*$^+$/*tgs-1*$^-$ subpopulations (Figures S3B–S3D) even after removing the *smedwi-1*$^+$ cells with the longest telomeres from the comparison (Figure S3C).

Starvation Increases the Percentage of Stem Cells with Long Telomeres through mTOR Signaling

To address how starvation affects stem cell biology, we studied telomere length on *smedwi-1*$^+$ cells comparing planarians that were starved for 7 days (7dS; standard metabolic status used to avoid background staining by food) with planarians that were starved for 20 days (20dS) (Figure 5A). Telomere fluorescence intensity quantification in *smedwi-1*$^+$ cells of 7dS planarians and in *smedwi-1*$^+$ of 20dS planarians showed significantly longer telomeres in 20dS planarian stem cells than those of planarians at 7dS, as reflected by higher median telomere fluorescence intensity (Figures 5B and 5C). Two alternative approaches to measure telomere length, telomere qPCR on whole-planarian genomic DNA (Figure S4A) and TelQ-FISH on X1 (stem cells) sorted cells (Figures S4B and S4C), confirmed the initial results showing that the median telomere fluorescence intensity of stem cells at 20dS is higher than that at 7dS.

During the process of starvation, planarians degrow in size (Gonzalez-Estevez et al., 2012a). To distinguish whether the observed effects of starvation on telomeres are due to the metabolic process itself or to the smaller size of the planarian, we compared size-matched planarians that were either at 7dS or 30dS. The median telomere fluorescence intensity of *smedwi-1*$^+$ cells at 30dS was higher than that of 7dS planarians (Figures S4D–S4F), similarly as with planarians of different metabolic status and size. In conclusion, starvation increases the percentage of stem cells with the longest telomeres.

mTOR signaling is the major nutrient sensor pathway that controls cell growth and proliferation in eukaryotes (Saxton and Sabatini, 2017). While inhibition of mTOR complex 1 (mTORC1) with rapamycin increases the life

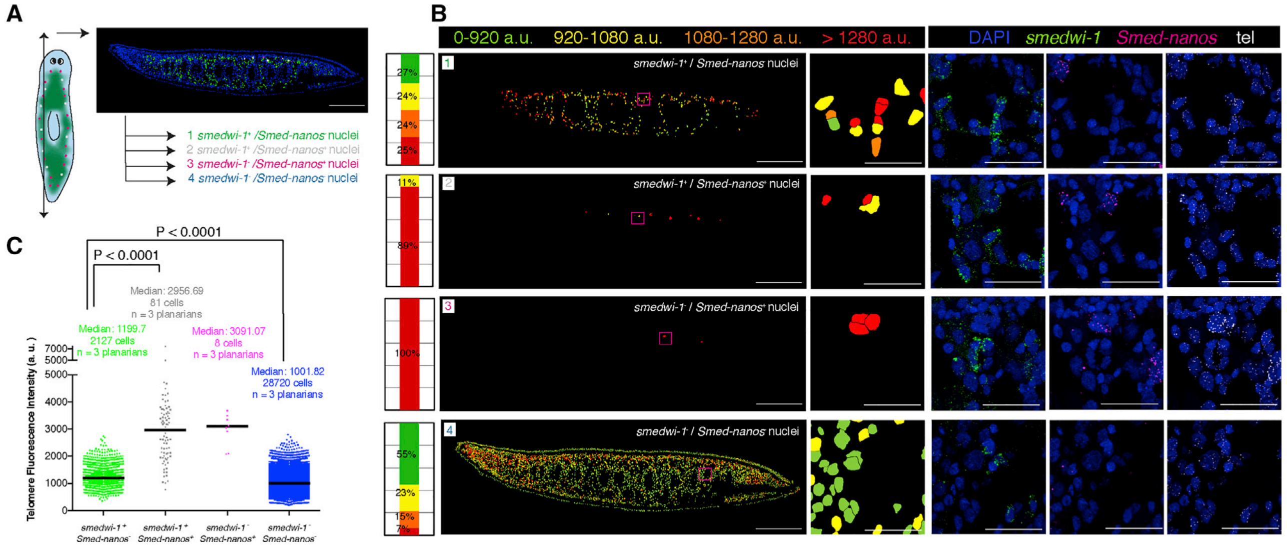

Figure 4. The Stem Cells with the Highest Telomere Intensity Are *nanos*+

(A) Schematic and tissue section indicate the distribution of four cell populations in a 7dS planarian. Double arrow indicates plane of sagittal sectioning.

(B) Representative tissue section with four telomere intensity maps that represent the four cell populations displayed in (A). The intensity maps show the nuclei colored according to their telomere fluorescence intensity. The stacked bar graphs represent the proportion of nuclei within a given category of intensity. *smedwi-1*+/*nanos*− is chosen as the reference population. Most of the stem cells with the longest telomere intensity are *nanos*+/*smedwi-1*+. Magenta box indicates the area shown. Anterior is to the left and dorsal is up.

(C) The column scatterplot shows all the pooled nuclei broken down into four cell populations. Any of the three subclasses of stem cells display longer telomeres than non-stem cells *smedwi-1*−/*nanos*− (two-tailed Mann-Whitney U test; $p < 0.0001$ for each of the three combinations). The median telomere intensity is higher in the *smedwi-1*+/*Smed-nanos*+ when compared with the *smedwi-1*+/*nanos*− (two-tailed Mann-Whitney U test; $p < 0.0001$). n, number of planarians analyzed.

Scale bars, 200 μm (main images) and 30 μm (high-magnification images). See also Figure S3.

span of many organisms and delays many age-associated diseases, its upregulation has been strongly linked to the progression of many cancers and to the aging process (Cornu et al., 2013). Since reduction of mTOR signaling has also shown to be one of the most important mechanisms responsible for the enhancement of stem cell function after DR (Huang et al., 2012; Yilmaz et al., 2012), we reasoned that mTOR signaling could be responsible for the increase in the percentage of stem cells with long telomeres during starvation that we observed. To test this theory, we performed RNAi experiments for the main components of mTOR signaling followed by FISH for *smedwi-1* and TelQ-FISH. Planarians were either injected with *Smed-tor* double-stranded RNA (dsRNA) (to inactivate the mTOR pathway) (Gonzalez-Estevez et al., 2012b; Peiris et al., 2012; Tu et al., 2012) or with *Smed-smg-1* dsRNA (to overactivate the mTOR pathway) (Gonzalez-Estevez et al., 2012b). Only planarians without apparent morphological phenotype were processed for telomere quantification to avoid loosening integrity in the tissues during the aggressive TelQ-FISH protocol (Figures 6A, 6B, S5A, and S5B). However, they already displayed a significant decrease in the number of mitoses in the case of *tor* RNAi (Figures S5C and S5D), and an increase in the case of *smg-1* RNAi (Figures S5E and S5F). Downregulation of *tor* further enhanced the effect of starvation on stem cell telomere length when compared with control injected worms (Figures 6C–6E). We also noticed that downregulation of *tor* is able to increase the maximum telomere length in stem cells (Figure 6E). This effect was also observed when comparing 7dS with 20dS (Figure 5C). This suggests that starvation and/or mTOR downregulation leads some stem cells to elongate their telomeres.

Remarkably, when comparing *smg-1* RNAi under starvation with starved or fed control planarians (Figure 6F), we observed that the telomere length distribution in *smg-1*(*RNAi*) stem cells is more similar to the controls in feeding conditions. Thus *smg-1* RNAi abolishes the effects of starvation on telomere elongation in stem cells. Furthermore, *smg-1* RNAi under feeding conditions (Figures S5G and S5H) only shows a slight decrease in the median telomere length with respect to fed controls (Figures 6F–6H), which suggests that the effects of *smg-1* RNAi on telomere length that are independent of the planarian caloric status are

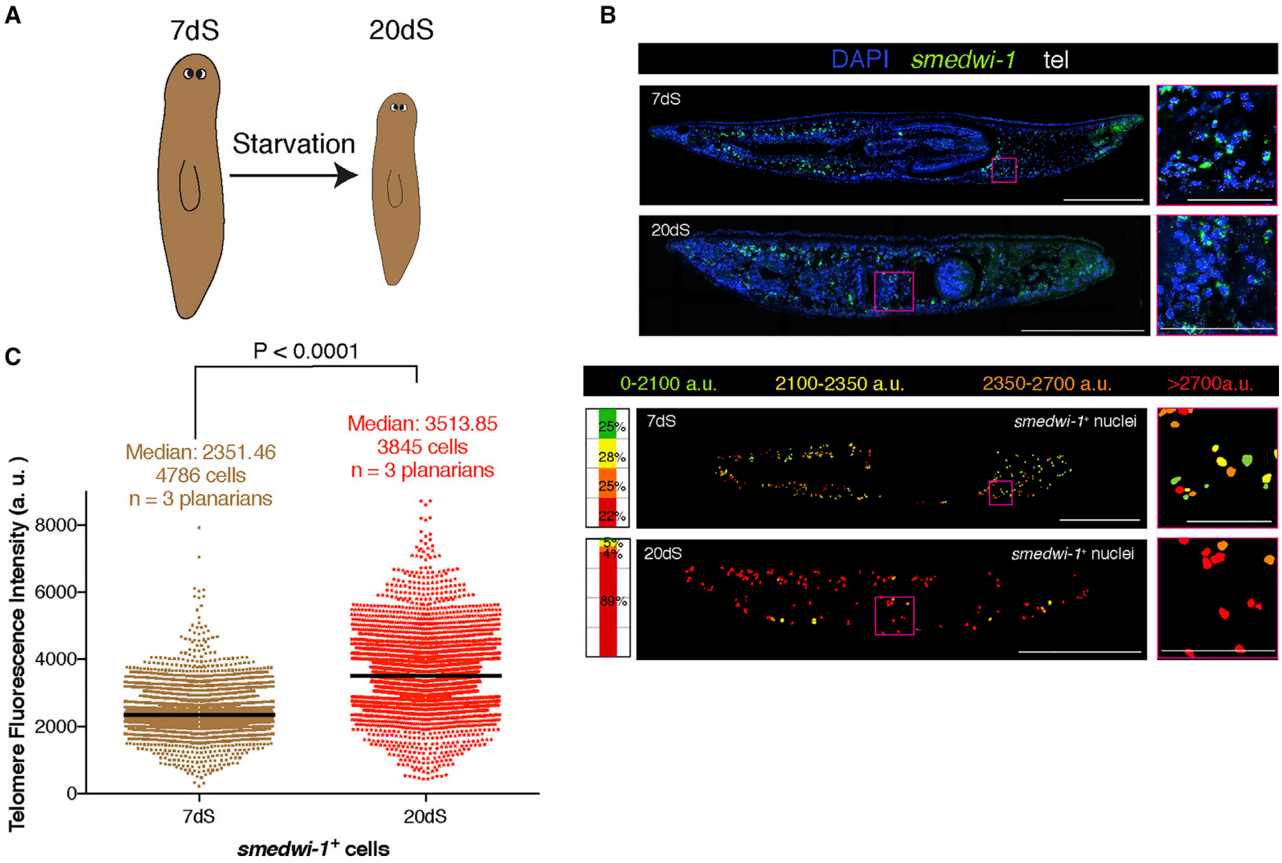

Figure 5. Starvation Increases the Percentage of Stem Cells with Long Telomeres

(A) During the process of starvation, planarians degrow in size.

(B) Maximum confocal projections for representative tissue sections of 7dS and 20dS planarian paraffin sections. Magenta box indicates the area shown. tel, telomeres; anterior is to the left and dorsal is up. Telomere intensity maps and stacked bar graphs for the representative tissue sections are also shown. The intensity maps display the nuclei colored according to their telomere fluorescence intensity. The stacked bar graphs represent the proportion of nuclei within a given category of intensity. 7dS is chosen as the reference condition. Magenta box indicates the area shown. Scale bars, 1 mm (main images) and 220 μm (high-magnification images).

(C) Column scatterplot showing all pooled nuclei. The median telomere intensity is higher in 20dS than in 7dS stem cells (two-tailed Mann-Whitney U test; $p < 0.0001$). n, number of planarians analyzed.

See also Figure S4.

minimal. In agreement with the phenotype of *smg-1* RNAi showing different degrees of penetrance in planarians (Gonzalez-Estevez et al., 2012b), we also obtained planarians in which the effects of starvation were either just slightly or strongly abolished (Figure 6H).

We also performed telomere qPCR on *gfp*(*RNAi*) and *tor*(*RNAi*) whole-planarian genomic DNA (Figure S6A) and TelQ-FISH on X1 (stem cells) sorted cells from *gfp*(*RNAi*) and *tor*(*RNAi*) planarians (Figures S6B and S6C), which confirmed our previous results showing that the median telomere fluorescence intensity of stem cells in *tor* RNAi is higher than that of controls.

We conclude that downregulation of *tor* enhances the effects of starvation on stem cell telomere length, while downregulation of *smg-1* (which increases mTOR signaling) does the opposite, abolishing the effects of starvation on telomere length in stem cells.

DISCUSSION

Telomere quantitative FISH has been widely used in a variety of tissues and organisms such as yeast, plants, zebrafish, or diverse mouse/human tissues. Planarians are an addition, and we envisage a wide use of the technique to study many aspects of aging, stem cell biology, DNA damage, and stress in the planarian model. Our work represents the first quantitative mapping of telomere length in whole

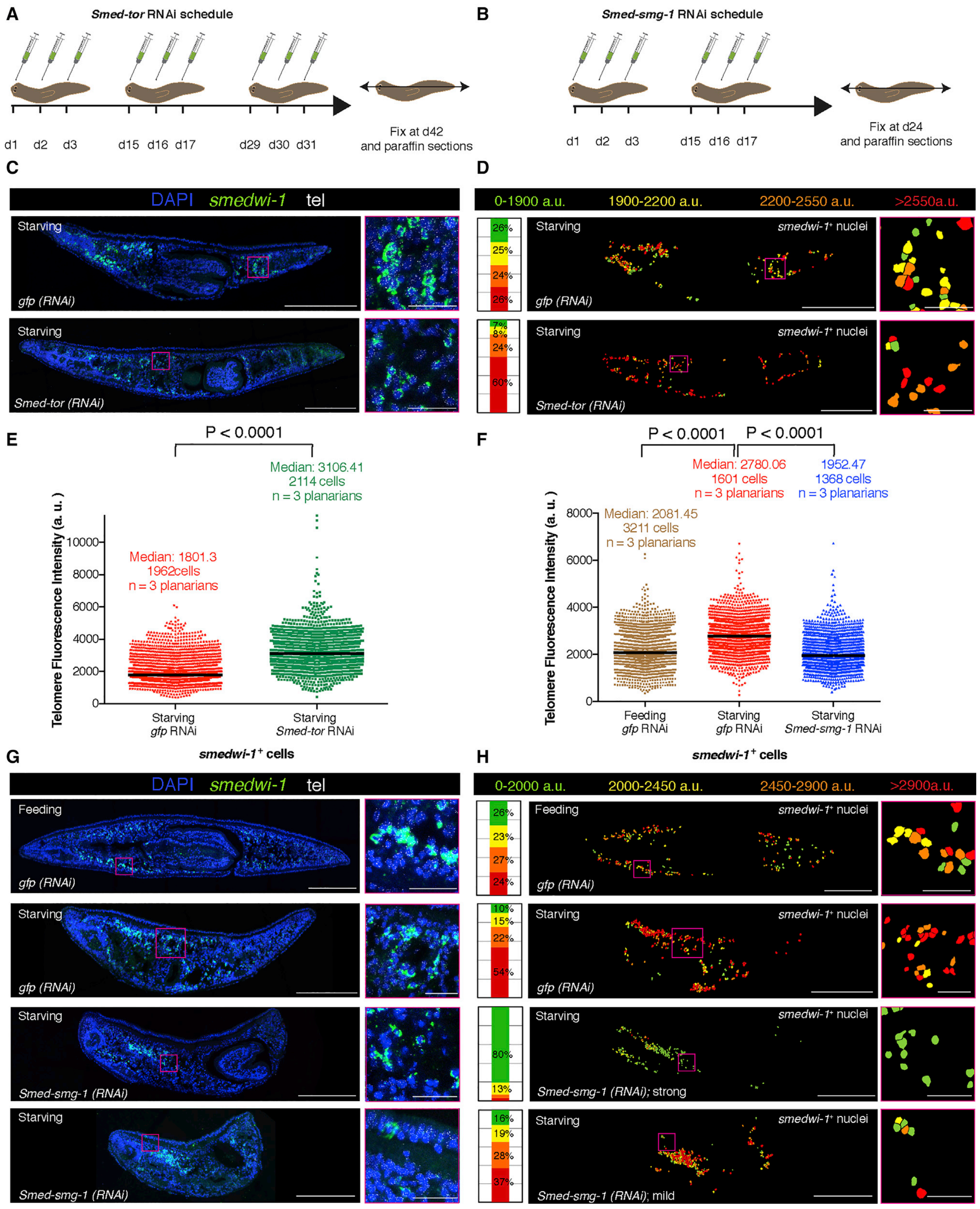

(legend on next page)

planarians including in planarian stem cells. Other *in vitro* experimental telomere quantitative technologies such as Southern blot analysis of terminal restriction fragment lengths have been previously applied to whole planarians (Tan et al., 2012) but cannot provide any detail about location and cell type. We have also adapted telomere length quantification to FACS-sorted cells as an alternative method to complement the data on paraffin tissue sections. Furthermore, telomere qPCR represents an easy method to confirm overall results.

Telomere length has been utilized to localize stem cell compartments in diverse mouse stem cell tissues (Aida et al., 2008; Flores et al., 2008). Our work demonstrates that telomere length quantification is able to distinguish between planarian stem cells and their progeny, as well as progeny of different ages. Our work sheds new light on a subclass of stem cells that co-express *smedwi-1*, a marker of somatic stem cells and *nanos*, a marker of germ cells in metazoans (Kobayashi et al., 1996; Koprunner et al., 2001). In sexually reproducing *S. mediterranea*, *nanos* is necessary for the development, maintenance, and regeneration of the germline but is also expressed in the asexually reproducing *S. mediterranea* strain (Handberg-Thorsager and Salo, 2007; Sato et al., 2006; Wang et al., 2007). Although the distribution of *nanos* in asexual planarians follows that of presumptive testes primordia of sexual planarians, asexually reproducing planarians will never differentiate gonads. The reason why putative germ stem cells exist in exclusively asexually reproducing planarians is currently unknown. One explanation suggested by previous studies is that these cells are vestigial remains of sexual reproduction, which have not yet been lost. However, it is well established that stem cells and germ cells are very alike; morphologically both look the same (Sato et al., 2006) and both share the expression of multiple regulators of germ cell development and known regulators of stem cell pluripotency (reviewed in Rink, 2013). In addition, we observe that *nanos* expression co-localizes with *smedwi-1* in the asexual strain. This is in agreement with *nanos*$^+$ cells in the asexual species *Dugesia japonica* co-labeling with the proliferative marker PCNA (Sato et al., 2006). In line with the conservative definition of germ cells as unipotent cells that give rise to one type of gamete (Yuan and Yamashita, 2010), it is currently thought that planarian germ cells (*nanos*$^+$ cells) arise from somatic stem cells and represent a more differentiated type of neoblast (Sato et al., 2006; Wang et al., 2007). If we consider telomere length as a tool to locate the more primitive stem cell compartments, an alternative model arises in which *nanos*$^+$ cells, which have the longest telomeres of all cells, would correspond to PSCs that could give rise to the rest of neoblasts in a hierarchical manner. It is a formal possibility that *nanos*$^+$ cells could be arrested, quiescent, or reserved stem cells that would contribute to the whole pool of somatic stem cells only in some contexts when they become activated. Since *nanos*$^+$ cells do not become mature germ cells, we would

Figure 6. mTOR Signaling Modulates the Effects of Starvation on Stem Cell Telomeres

(A) RNAi schedule for *tor* RNAi and controls.

(B) RNAi schedule for *smg-1* RNAi and controls.

(C) Maximum confocal projections for representative tissue sections of control planarians (starving) and *tor* RNAi (starving). Magenta box indicates the area shown.

(D) Telomere intensity maps and stacked bar graphs for the *smedwi-1*$^+$ cells in the representative tissue section shown in (C). The maps display the nuclei colored according to their telomere fluorescence intensity. The stacked bar graphs represent the proportion of nuclei within a given category of intensity. *gfp* RNAi is chosen as the reference condition. *tor* RNAi have a higher percentage of stem cells with long telomeres. Magenta box indicates the area shown.

(E) Column scatterplot showing all the pooled cells. The median telomere intensity is higher in *tor* RNAi than in controls (two-tailed Mann-Whitney U test; $p < 0.0001$).

(F) Column scatterplot showing all the pooled cells. The median telomere intensity is higher in controls starving than in controls feeding (two-tailed Mann-Whitney U test; $p < 0.0001$). *smg-1* RNAi (starving) abolishes the effects of starvation (two-tailed Mann-Whitney U test; $p < 0.0001$). *smg-1* RNAi (starving) has an even lower median than fed controls (two-tailed Mann-Whitney U test; $p < 0.0001$). Arbitrary units are not comparable between (E) and (F) for being two independent experiments.

(G) Maximum confocal projections for representative tissue sections of control planarians either under starving or feeding conditions and *smg-1*(*RNAi*) planarians under starving conditions. Two images are displayed for *smg-1* RNAi (starving), which represent different degrees of phenotype penetrance. Magenta box indicates the area shown.

(H) Telomere intensity maps and stacked bar graphs for the *smedwi-1*$^+$ cells in the representative tissue sections shown in (G). The maps display the nuclei colored according to their telomere fluorescence intensity. The stacked bar graphs represent the proportion of nuclei within a given category of intensity. *gfp* RNAi (feeding) is chosen as the reference. *gfp* RNAi (starving) shows a higher percentage of stem cells with long telomeres, whereas this effect is abolished when planarians are injected with *smg-1* RNAi. Magenta box indicates the area shown.

tel, telomeres; n, number of planarians analyzed; anterior is to the left and dorsal is up. Scale bars, 200 μm (main images) and 30 μm (high-magnification images). See also Figures S5 and S6.

expect that the arrest of a Tert+ cell could lead to elongation of telomeres beyond that which would be seen in sexual animals. Further experiments are needed to clarify whether *nanos*+ cells can give rise to somatic stem cells.

We also analyzed the distribution of telomere length in the stem cells positive for the marker *tgs-1*, which is known to belong to a subpopulation that represents 25% of the total *smedwi-1*+ stem cells in the planarian body and contains PSCs among other stem cells (Zeng et al., 2018). Our data showed that both populations of stem cells *smedwi-1*+/*tgs-1*+ and *smedwi-1*+/*tgs-1*− have the same telomere distribution and that *smedwi-1*+/*tgs-1*+ cells do not include the stem cells with the longest telomeres. Since it is not known how many stem cells positive for *tgs-1* are truly pluripotent, it could still be that *tgs-1*+ stem cells with the longest telomeres are pluripotent or can go back to be pluripotent in some contexts. Future studies involving *in vivo* experiments will need to address whether planarian stem cells with the longest telomeres are PSCs.

DR leads to maintenance or elongation of telomeres and increased telomerase activity in various adult mouse tissues (Vera et al., 2013). However, there is currently no evidence that DR or fasting may be influencing telomere length specifically on stem cells and thus contributing to the reported increased stem cell functionality after dietary intervention. Here we show that starvation increases the population of stem cells with long telomeres in the planarian model and that at least some stem cells are able to significantly elongate telomere length at 20dS when compared with 7dS. Considering that planarians maintain a stable pool of dynamically dividing stem cells and reduce the rate of differentiation over the course of starvation (Gonzalez-Estevez et al., 2012a), increasing telomere length seems a good strategy to avoid an increased risk of stem cell aging and, thus, aging in the whole planarian. Remarkably, previous work by means of Southern blot analysis on planarian genomic preparations has shown that global average telomere length increases after regeneration or fission following a long period of no regeneration/fission in asexual planarians (Tan et al., 2012). Thus, through telomere maintenance during regeneration/fission and starvation, two processes that are continuously occurring during planarian live history, planarians may be able to maintain a "young" population of stem cells, and therefore to be considered immortal.

Although downregulation of mTOR signaling is the major mechanism for the enhancement of stem cell function after DR (Huang et al., 2012; Yilmaz et al., 2012), only a few works suggest that mTOR signaling could be coupled to telomerase and telomere length regulation (Kawauchi et al., 2005). Our data show that while downregulation of mTOR enhances the effect of starvation on the telomeres of planarian stem cells, upregulation of mTOR signaling (by downregulating of the negative regulator of mTOR signaling, SMG-1) is able to abolish these effects. In planarians, mTOR signaling is a key regulator of the first response to wounding during regeneration (Gonzalez-Estevez et al., 2012b; Peiris et al., 2012; Tu et al., 2012). However, there are few data about how mTOR signaling regulates starvation in planarians. mTOR signaling is downregulated during starvation as observed in other organisms (Peiris et al., 2012). RNAi experiments on members of mTORC1 or rapamycin treatment during starvation have shown a reduction in the number of mitotic neoblasts and in the whole pool of *smedwi-1*+ stem cells (Gonzalez-Estevez et al., 2012b; Peiris et al., 2012). In a similar way, downregulation of planarian PTEN homologs (negative regulators of the pathway) increases the number of mitoses in starved planarians (Oviedo et al., 2008). Here we have also shown that while *tor* RNAi reduces the number of mitoses by day 42 of starvation, *smg-1* RNAi already increases mitoses by 24 days. Since telomeres shorten progressively with every cell division (Flores and Blasco, 2010), one possible explanation is that the telomere effects that we see here could be a secondary consequence of stem cell perturbation rather than a consequence of changes in mTOR signaling. However, while during starvation telomere length increases, which we observed is dependent on mTOR signaling, the number of mitoses and stem cells are maintained (Gonzalez-Estevez et al., 2012a). Indeed, it is known that other factors modulate telomere length, for instance oxidative stress, oxygen levels, and, in general, mild stress (Flores et al., 2008; von Zglinicki, 2002; von Zglinicki et al., 1995). Together these findings make feasible the idea of starvation and mTOR signaling directly regulating telomere length by other mechanisms than cell division. Any of these factors could also explain our results of the shortening of median telomere length from late to early post-mitotic progeny, two cell planarian populations that transition without cell division.

It has also been described that *tor*(*RNAi*) planarians are still able to degrow at the same rate as controls while having increased levels of cell death (Peiris et al., 2012), which indicates that some stem cells are dying. Remarkably our data show that the subpopulation of stem cells with short telomeres reduces during starvation (downregulation of mTOR) and disappears after *tor* RNAi (stronger downregulation of mTOR) (Figure S6D). A number of different models could explain how mTOR activity regulates the stem cell population telomere length distribution. These include the direct regulation of telomerase activity or through regulating which stem cells divide symmetrically or asymmetrically using existing telomere length as one of the inputs to this decision. Since mTOR signaling can determine fitness through cell competition (Bowling et al., 2018; Claveria and Torres, 2016), another possibility is that the stem cells

with shorter telomeres are more likely to die/differentiate when mTOR activity is reduced on average while stem cells with long telomeres are competitively selected to self-renew. Future work using the experimental paradigm we establish here will provide further insight into how nutritional status affects adult stem cell biology.

EXPERIMENTAL PROCEDURES

Animal Husbandry

Planarians in this work belong to the species *S. mediterranea* asexual strain and were maintained at 19°C in 1× Montjuïc salts (1.6 mM NaCl, 1.0 mM $CaCl_2$, 1.0 mM $MgSO_4$, 0.1 mM $MgCl_2$, 0.1 mM KCl, 1.2 mM $NaHCO_3$). Planarians were fed organic veal liver.

RNAi Experiments

We generated templates with T7 promoters appended to both strands from *Smed-smg-1*, *Smed-tor*, and *Smed-tert*, synthesized dsRNA by *in vitro* transcription (Roche), and injected these into planarians as described previously (Gonzalez-Estevez et al., 2012b; Tan et al., 2012). Control animals were injected with *gfp* dsRNA, a sequence not present in *S. mediterranea*. A detailed protocol can be found in Supplemental Experimental Procedures.

In Situ Hybridization on Planarian Sections

Single and double FISH were performed as previously described (Solana, 2018). A detailed protocol can be found in Supplemental Experimental Procedures.

Immunohistochemistry after FISH on Planarian Sections

Immnunohistochemistry after FISH was performed as described by Solana (2018). A mouse polyclonal SMEDWI-1 antibody was generated using an already reported peptide (Guo et al., 2006) and affinity purified (GenScript). The antibody was used at a 1/100 dilution. An AlexaFluor 488 goat anti-mouse immunoglobulin G (H + L) Antibody (Life Technologies) was used as secondary antibody.

Telomere Quantitative Fluorescent *In Situ* Hybridization

TelQ-FISH, image acquisition, and quantifications were performed as previously described for mouse tissue sections (Flores et al., 2008; Gonzalez-Suarez et al., 2000; Zijlmans et al., 1997). For a detailed protocol please refer to Supplemental Experimental Procedures.

Statistical Analysis

Since most of the TelQ-FISH samples analyzed in herein (26/28) failed the D'Agostino and Pearson normality test (only *smedwi-1*$^-$/SMEDWI-1$^+$ [p = 0.4590] and *smedwi-1*$^-$/*Smed-nanos*$^+$ [p = 0.5591] passed the test) and neither followed a log-normal distribution, we used non-parametric statistical tests to evaluate significance. Kruskal-Wallis one-way analysis of variance and/or two-tailed Mann-Whitney U test was used to evaluate significance. Two-tailed Student's t test with equal sample variance was used for telomere qPCR and H3P quantification; error bars represent standard deviation of the mean. All the tests were performed with GraphPad Prism 7.0d.

SUPPLEMENTAL INFORMATION

Supplemental Information can be found online at https://doi.org/10.1016/j.stemcr.2019.06.005.

AUTHOR CONTRIBUTIONS

M.I. and C.G.-E. performed most of the experiments; D.A.F., O.G.-G., S.S., and A.A.A. helped to perform some experiments; B.F.-V. and R.P. performed the telomere qPCRs; M.I., I.F., and C.G.-E. designed experiments and analyzed the data; C.G.-E. performed most of the telomere quantification analyses with the help of D.A.F. and O.G.-G.; M.d.M.d.M.B. contributed to the initial setting of planarian telomapping and obtained some preliminary data; I.F. and C.G.-E. directed the project; C.G.-E. wrote the manuscript; M.I., D.A.F., O.G.-G., A.A.A., and I.F. contributed to the editing of the manuscript.

ACKNOWLEDGMENTS

We would like to thank the Imaging Core Facility and the Core Histology Service from the Leibniz Institute on Aging-Fritz Lipmann Institute (FLI) for their technical support. We would also like to thank E. Arza and A.M. Santos from the CNIC Microscopy Unit and also J. Solana, C. Martín-Durán, R. Romero, L. Sastre, E.G. Arias-Salgado, and all past and current members in the C.G.-E. and I.F. labs. C.G.-E. was funded by a Contrato de Investigadores Miguel Servet (CP12/03214) and by the FLI. The FLI is a member of the Leibniz Association and is financially supported by the Federal Government of Germany and the State of Thuringia. O.G.-G. was funded by an LGSA scholarship. R.P. and B.F.-V. were funded by a grant (PI17-01401) from Fondo de Investigaciones Sanitarias (Instituto de Salud Carlos III, Spain) and FEDER funds. I.F. was funded by grants from Ministerio de Ciencia, Innovación y Universidades (SAF2016-80406-R), Comunidad de Madrid (S2017/BMD-3875), and the Red Temática de Investigación Cooperativa en Enfermedades Cardiovasculares (RD12/0042/0045). The CNIC is supported by the Ministerio de Ciencia, Innovación y Universidades and the Pro CNIC Foundation, and is a Severo Ochoa Center of Excellence (SEV-2015-0505). A.A.A. was funded by grants from the BBSRC (BB/K007564/1) and MRC (MR/M000133/1), and S.S. by a University of Oxford Clarendon Fund Scholarship.

Received: September 22, 2018
Revised: June 22, 2019
Accepted: June 24, 2019
Published: July 25, 2019

REFERENCES

Aboobaker, A.A. (2011). Planarian stem cells: a simple paradigm for regeneration. Trends Cell Biol. *21*, 304–311.

Aida, J., Izumiyama-Shimomura, N., Nakamura, K., Ishikawa, N., Poon, S.S., Kammori, M., Sawabe, M., Arai, T., Matsuura, M.,

Fujiwara, M., et al. (2008). Basal cells have longest telomeres measured by tissue Q-FISH method in lingual epithelium. Exp. Gerontol. *43*, 833–839.

Angelo, G., and Van Gilst, M.R. (2009). Starvation protects germline stem cells and extends reproductive longevity in *C. elegans*. Science *326*, 954–958.

Baguñà, J. (1976). Mitosis in the intact and regenerating planarian *Dugesia mediterranea* n. sp. I. Mitotic studies during growth, feeding and starvation. J. Exp. Zool. *195*, 53–64.

Behrens, A., van Deursen, J.M., Rudolph, K.L., and Schumacher, B. (2014). Impact of genomic damage and ageing on stem cell function. Nat. Cell Biol. *16*, 201–207.

Bowling, S., Di Gregorio, A., Sancho, M., Pozzi, S., Aarts, M., Signore, M., Schneider, M.D., Barbera, J.P.M., Gil, J., and Rodriguez, T.A. (2018). P53 and mTOR signalling determine fitness selection through cell competition during early mouse embryonic development. Nat. Commun. *9*, 1763.

Cerletti, M., Jang, Y.C., Finley, L.W., Haigis, M.C., and Wagers, A.J. (2012). Short-term calorie restriction enhances skeletal muscle stem cell function. Cell Stem Cell *10*, 515–519.

Claveria, C., and Torres, M. (2016). Cell competition: mechanisms and physiological roles. Annu. Rev. Cell Dev. Biol. *32*, 411–439.

Cornu, M., Albert, V., and Hall, M.N. (2013). mTOR in aging, metabolism, and cancer. Curr. Opin. Genet. Dev. *23*, 53–62.

Eisenhoffer, G.T., Kang, H., and Sanchez Alvarado, A. (2008). Molecular analysis of stem cells and their descendants during cell turnover and regeneration in the planarian *Schmidtea mediterranea*. Cell Stem Cell *3*, 327–339.

Felix, D.A., Gutierrez-Gutierrez, O., Espada, L., Thems, A., and Gonzalez-Estevez, C. (2019). It is not all about regeneration: planarians striking power to stand starvation. Semin. Cell Dev. Biol. *87*, 169–181.

Flores, I., and Blasco, M.A. (2010). The role of telomeres and telomerase in stem cell aging. FEBS Lett. *584*, 3826–3830.

Flores, I., Canela, A., Vera, E., Tejera, A., Cotsarelis, G., and Blasco, M.A. (2008). The longest telomeres: a general signature of adult stem cell compartments. Genes Dev. *22*, 654–667.

Garcia-Lavandeira, M., Quereda, V., Flores, I., Saez, C., Diaz-Rodriguez, E., Japon, M.A., Ryan, A.K., Blasco, M.A., Dieguez, C., Malumbres, M., et al. (2009). A GRFa2/Prop1/stem (GPS) cell niche in the pituitary. PLoS One *4*, e4815.

Gilson, E., and Londono-Vallejo, A. (2007). Telomere length profiles in humans: all ends are not equal. Cell Cycle *6*, 2486–2494.

Gonzalez-Estevez, C., Felix, D.A., Rodriguez-Esteban, G., and Aboobaker, A.A. (2012a). Decreased neoblast progeny and increased cell death during starvation-induced planarian degrowth. Int. J. Dev. Biol. *56*, 83–91.

Gonzalez-Estevez, C., Felix, D.A., Smith, M.D., Paps, J., Morley, S.J., James, V., Sharp, T.V., and Aboobaker, A.A. (2012b). SMG-1 and mTORC1 act antagonistically to regulate response to injury and growth in planarians. PLoS Genet. *8*, e1002619.

Gonzalez-Garcia, M.P., Pavelescu, I., Canela, A., Sevillano, X., Leehy, K.A., Nelson, A.D., Ibanes, M., Shippen, D.E., Blasco, M.A., and Cano-Delgado, A.I. (2015). Single-cell telomere-length quantification couples telomere length to meristem activity and stem cell development in *Arabidopsis*. Cell Rep. *11*, 977–989.

Gonzalez-Suarez, E., Samper, E., Flores, J.M., and Blasco, M.A. (2000). Telomerase-deficient mice with short telomeres are resistant to skin tumorigenesis. Nat. Genet. *26*, 114–117.

Guo, T., Peters, A.H., and Newmark, P.A. (2006). A Bruno-like gene is required for stem cell maintenance in planarians. Dev. Cell *11*, 159–169.

Handberg-Thorsager, M., and Salo, E. (2007). The planarian nanos-like gene Smednos is expressed in germline and eye precursor cells during development and regeneration. Dev. Genes Evol. *217*, 403–411.

Huang, J., Nguyen-McCarty, M., Hexner, E.O., Danet-Desnoyers, G., and Klein, P.S. (2012). Maintenance of hematopoietic stem cells through regulation of Wnt and mTOR pathways. Nat. Med. *18*, 1778–1785.

Huang, J., Wang, F., Okuka, M., Liu, N., Ji, G., Ye, X., Zuo, B., Li, M., Liang, P., Ge, W.W., et al. (2011). Association of telomere length with authentic pluripotency of ES/iPS cells. Cell Res. *21*, 779–792.

Kawauchi, K., Ihjima, K., and Yamada, O. (2005). IL-2 increases human telomerase reverse transcriptase activity transcriptionally and posttranslationally through phosphatidylinositol 3'-kinase/Akt, heat shock protein 90, and mammalian target of rapamycin in transformed NK cells. J. Immunol. *174*, 5261–5269.

Kobayashi, S., Yamada, M., Asaoka, M., and Kitamura, T. (1996). Essential role of the posterior morphogen nanos for germline development in Drosophila. Nature *380*, 708–711.

Koprunner, M., Thisse, C., Thisse, B., and Raz, E. (2001). A zebrafish nanos-related gene is essential for the development of primordial germ cells. Genes Dev. *15*, 2877–2885.

Longo, V.D., and Mattson, M.P. (2014). Fasting: molecular mechanisms and clinical applications. Cell Metab. *19*, 181–192.

Lopez-Otin, C., Blasco, M.A., Partridge, L., Serrano, M., and Kroemer, G. (2013). The hallmarks of aging. Cell *153*, 1194–1217.

Mangel, M., Bonsall, M.B., and Aboobaker, A. (2016). Feedback control in planarian stem cell systems. BMC Syst. Biol. *10*, 17.

Mihaylova, Y., Abnave, P., Kao, D., Hughes, S., Lai, A., Jaber-Hijazi, F., Kosaka, N., and Aboobaker, A.A. (2018). Conservation of epigenetic regulation by the MLL3/4 tumour suppressor in planarian pluripotent stem cells. Nat. Commun. *9*, 3633.

Molenaar, C., Wiesmeijer, K., Verwoerd, N.P., Khazen, S., Eils, R., Tanke, H.J., and Dirks, R.W. (2003). Visualizing telomere dynamics in living mammalian cells using PNA probes. EMBO J. *22*, 6631–6641.

Oviedo, N.J., Pearson, B.J., Levin, M., and Sanchez Alvarado, A. (2008). Planarian PTEN homologs regulate stem cells and regeneration through TOR signaling. Dis. Model. Mech. *1*, 131–143, discussion 141.

Peiris, T.H., Weckerle, F., Ozamoto, E., Ramirez, D., Davidian, D., Garcia-Ojeda, M.E., and Oviedo, N.J. (2012). TOR signaling regulates planarian stem cells and controls localized and organismal growth. J. Cell Sci. *125*, 1657–1665.

Pellettieri, J. (2019). Regenerative tissue remodeling in planarians - The mysteries of morphallaxis. Semin. Cell Dev. Biol. *87*, 13–21.

Pucci, F., Gardano, L., and Harrington, L. (2013). Short telomeres in ESCs lead to unstable differentiation. Cell Stem Cell *12*, 479–486.

Reddien, P.W., Oviedo, N.J., Jennings, J.R., Jenkin, J.C., and Sanchez Alvarado, A. (2005). SMEDWI-2 is a PIWI-like protein that regulates planarian stem cells. Science *310*, 1327–1330.

Richmond, C.A., Shah, M.S., Deary, L.T., Trotier, D.C., Thomas, H., Ambruzs, D.M., Jiang, L., Whiles, B.B., Rickner, H.D., Montgomery, R.K., et al. (2015). Dormant intestinal stem cells are regulated by PTEN and nutritional status. Cell Rep. *13*, 2403–2411.

Rink, J.C. (2013). Stem cell systems and regeneration in planaria. Dev. Genes Evol. *223*, 67–84.

Sato, K., Shibata, N., Orii, H., Amikura, R., Sakurai, T., Agata, K., Kobayashi, S., and Watanabe, K. (2006). Identification and origin of the germline stem cells as revealed by the expression of nanos-related gene in planarians. Dev. Growth Differ. *48*, 615–628.

Saxton, R.A., and Sabatini, D.M. (2017). mTOR signaling in growth, metabolism, and disease. Cell *169*, 361–371.

Schneider, R.P., Garrobo, I., Foronda, M., Palacios, J.A., Marion, R.M., Flores, I., Ortega, S., and Blasco, M.A. (2013). TRF1 is a stem cell marker and is essential for the generation of induced pluripotent stem cells. Nat. Commun. *4*, 1946.

Scimone, M.L., Kravarik, K.M., Lapan, S.W., and Reddien, P.W. (2014). Neoblast specialization in regeneration of the planarian *Schmidtea mediterranea*. Stem Cell Rep. *3*, 339–352.

Solana, J. (2018). RNA in situ hybridization on planarian paraffin sections. Methods Mol. Biol. *1774*, 393–404.

Tan, T.C., Rahman, R., Jaber-Hijazi, F., Felix, D.A., Chen, C., Louis, E.J., and Aboobaker, A. (2012). Telomere maintenance and telomerase activity are differentially regulated in asexual and sexual worms. Proc. Natl. Acad. Sci. U S A *109*, 4209–4214.

Tang, D., Tao, S., Chen, Z., Koliesnik, I.O., Calmes, P.G., Hoerr, V., Han, B., Gebert, N., Zornig, M., Loffler, B., et al. (2016). Dietary restriction improves repopulation but impairs lymphoid differentiation capacity of hematopoietic stem cells in early aging. J. Exp. Med. *213*, 535–553.

Tinkum, K.L., Stemler, K.M., White, L.S., Loza, A.J., Jeter-Jones, S., Michalski, B.M., Kuzmicki, C., Pless, R., Stappenbeck, T.S., Piwnica-Worms, D., et al. (2015). Fasting protects mice from lethal DNA damage by promoting small intestinal epithelial stem cell survival. Proc. Natl. Acad. Sci. U S A *112*, E7148–E7154.

Tu, K.C., Pearson, B.J., and Sanchez Alvarado, A. (2012). TORC1 is required to balance cell proliferation and cell death in planarians. Dev. Biol. *365*, 458–469.

van Wolfswinkel, J.C., Wagner, D.E., and Reddien, P.W. (2014). Single-cell analysis reveals functionally distinct classes within the planarian stem cell compartment. Cell Stem Cell *15*, 326–339.

Vera, E., Bernardes de Jesus, B., Foronda, M., Flores, J.M., and Blasco, M.A. (2013). Telomerase reverse transcriptase synergizes with calorie restriction to increase health span and extend mouse longevity. PLoS One *8*, e53760.

von Zglinicki, T. (2002). Oxidative stress shortens telomeres. Trends Biochem. Sci. *27*, 339–344.

von Zglinicki, T., Saretzki, G., Docke, W., and Lotze, C. (1995). Mild hyperoxia shortens telomeres and inhibits proliferation of fibroblasts: a model for senescence? Exp. Cell Res. *220*, 186–193.

Wagner, D.E., Wang, I.E., and Reddien, P.W. (2011). Clonogenic neoblasts are pluripotent adult stem cells that underlie planarian regeneration. Science *332*, 811–816.

Wang, Y., Zayas, R.M., Guo, T., and Newmark, P.A. (2007). Nanos function is essential for development and regeneration of planarian germ cells. Proc. Natl. Acad. Sci. U S A *104*, 5901–5906.

Wenemoser, D., and Reddien, P.W. (2010). Planarian regeneration involves distinct stem cell responses to wounds and tissue absence. Dev. Biol. *344*, 979–991.

Yilmaz, O.H., Katajisto, P., Lamming, D.W., Gultekin, Y., Bauer-Rowe, K.E., Sengupta, S., Birsoy, K., Dursun, A., Yilmaz, V.O., Selig, M., et al. (2012). mTORC1 in the Paneth cell niche couples intestinal stem-cell function to calorie intake. Nature *486*, 490–495.

Yuan, H., and Yamashita, Y.M. (2010). Germline stem cells: stems of the next generation. Curr. Opin. Cell Biol. *22*, 730–736.

Zeng, A., Li, H., Guo, L., Gao, X., McKinney, S., Wang, Y., Yu, Z., Park, J., Semerad, C., Ross, E., et al. (2018). Prospectively isolated Tetraspanin(+) neoblasts are adult pluripotent stem cells underlying planaria regeneration. Cell *173*, 1593–1608.e20.

Zijlmans, J.M., Martens, U.M., Poon, S.S., Raap, A.K., Tanke, H.J., Ward, R.K., and Lansdorp, P.M. (1997). Telomeres in the mouse have large inter-chromosomal variations in the number of T2AG3 repeats. Proc. Natl. Acad. Sci. U S A *94*, 7423–7428.

Stem Cell Reports, Volume *13*

Supplemental Information

Downregulation of mTOR Signaling Increases Stem Cell Population Telomere Length during Starvation of Immortal Planarians

Marta Iglesias, Daniel A. Felix, Óscar Gutiérrez-Gutiérrez, Maria del Mar De Miguel-Bonet, Sounak Sahu, Beatriz Fernández-Varas, Rosario Perona, A. Aziz Aboobaker, Ignacio Flores, and Cristina González-Estévez

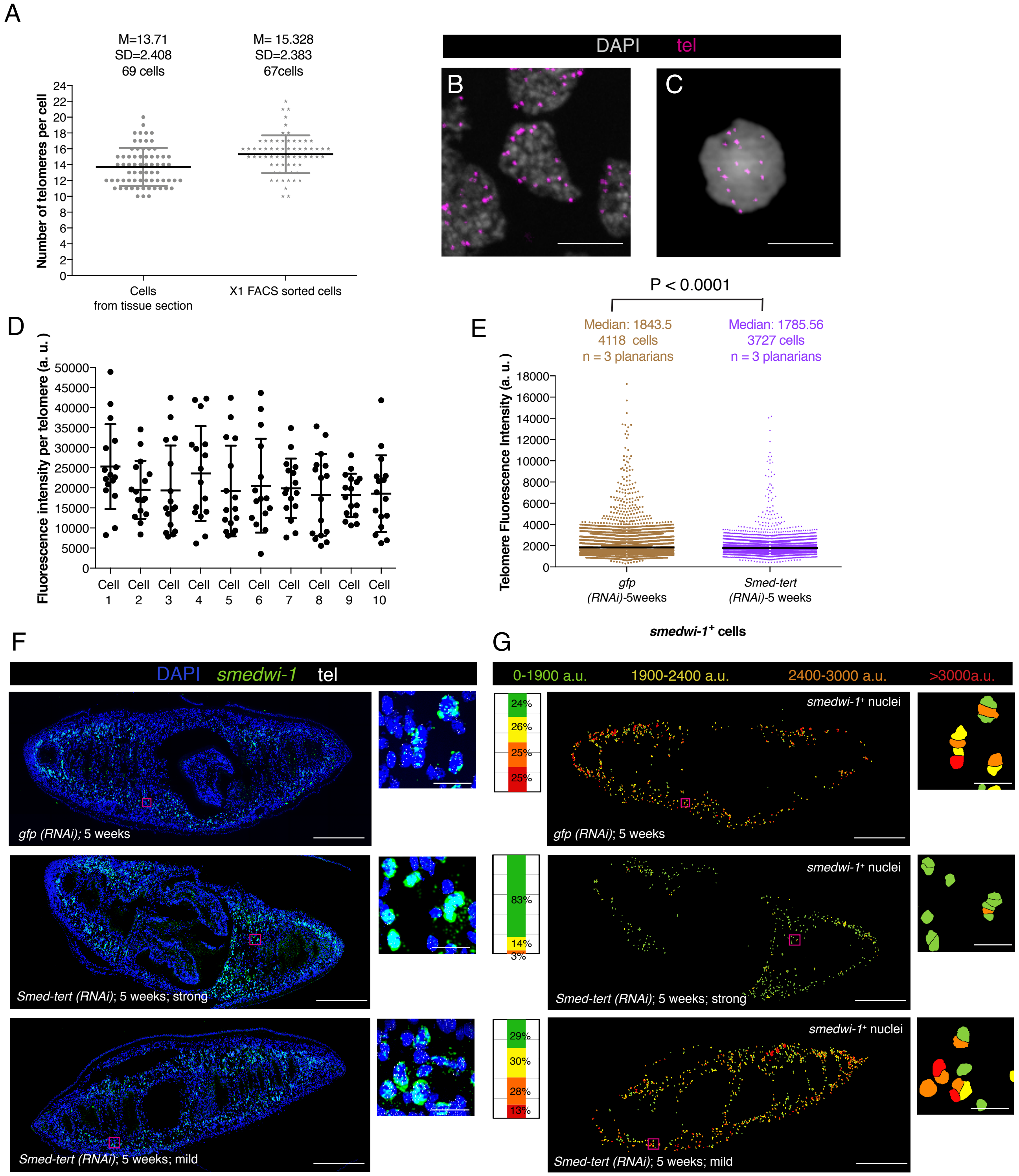
A
M=13.71
SD=2.408
69 cells
M= 15.328
SD=2.383
67cells
Number of telomeres per cell
Cells from tissue section
X1 FACS sorted cells
DAPI tel
B
C
D
Fluorescence intensity per telomere (a. u.)
Cell 1
Cell 2
Cell 3
Cell 4
Cell 5
Cell 6
Cell 7
Cell 8
Cell 9
Cell 10
E
P < 0.0001
Median: 1843.5
4118 cells
n = 3 planarians
Median: 1785.56
3727 cells
n = 3 planarians
Telomere Fluorescence Intensity (a. u.)
gfp (RNAi)-5weeks
Smed-tert (RNAi)-5 weeks
F
DAPI smedwi-1 tel
gfp (RNAi); 5 weeks
Smed-tert (RNAi); 5 weeks; strong
Smed-tert (RNAi); 5 weeks; mild
G
smedwi-1+ cells
0-1900 a.u.
1900-2400 a.u.
2400-3000 a.u.
>3000a.u.
smedwi-1+ nuclei
24%
26%
25%
25%
83%
14%
3%
29%
30%
28%
13%

Figure S1. Validation of TelQ-FISH in planarians. Related to Figure 1. (A) Quantification of the number of telomeres in cells of a tissue section and in FACS sorted stem cells (X1 fraction). The mean (M) number of telomeres per cell and the total number of cells analyzed is displayed. SD, standard deviation. (B) Image of cells from a tissue section. The cell in the middle of the image shows 16 telomeres. (C) Image of a FACS sorted stem cell (X1) showing 16 telomeres. Scale bars indicate 10 µm. (D) Column scatter plot showing the total fluorescence intensity of every telomere from 10 different FACS sorted stem cells (X1). The error bars indicate deviation from the mean. Cell 6 shows the highest spread of values (longest error bar) while cell 9 shows the lowest (shortest error bar). (E) Column scatter plot showing all the cells pooled from a total of 3 planarians for each condition. The median telomere intensity is higher in *gfp(RNAi)* than in *tert(RNAi)* stem cells (two-tailed Mann-Whitney U test; $P < 0.0001$). n indicates the number of planarians analyzed; a. u. indicates arbitrary units. (F) Maximum projections for representative tissue sections from E of *gfp* injected planarians and *tert(RNAi)* injected planarians labeled with the stem cell marker *smedwi-1*. All are also labeled for telomeres and counterstained for DAPI. All represent 5 weeks of RNAi treatment. Two images are displayed for *tert* RNAi which represent different degrees of phenotype penetrance (mild and strong). The magenta squares indicate the area of magnification displayed next to the main images. tel indicates telomeres; anterior is to the left and dorsal is up. Scale bars indicate 250 µm in the main images and 30 µm in the high magnification images. (G) Stacked bar graphs and telomere intensity maps for the *smedwi-1*$^+$ cells in the representative tissue sections showed in F. The maps display the nuclei coloured according to their telomere fluorescence intensity (four categories of intensity). The stacked bar graphs represent the proportion of nuclei within a given category of intensity. *gfp(RNAi)* condition is chosen as the reference condition and set up to allocate in each range of intensity or category approximately one fourth of the total cells. *tert(RNAi)* condition shows a higher percentage of stem cells with short telomeres and a lower percentage of stem cells with long telomeres than in controls. Different degrees in the *tert(RNAi)* phenotype are presented (mild and strong). Magenta squares indicate the area of magnification displayed next to the main images. a.u. indicates arbitrary units. Scale bars indicate 250 µm in the main images and 30 µm in the high magnification images.

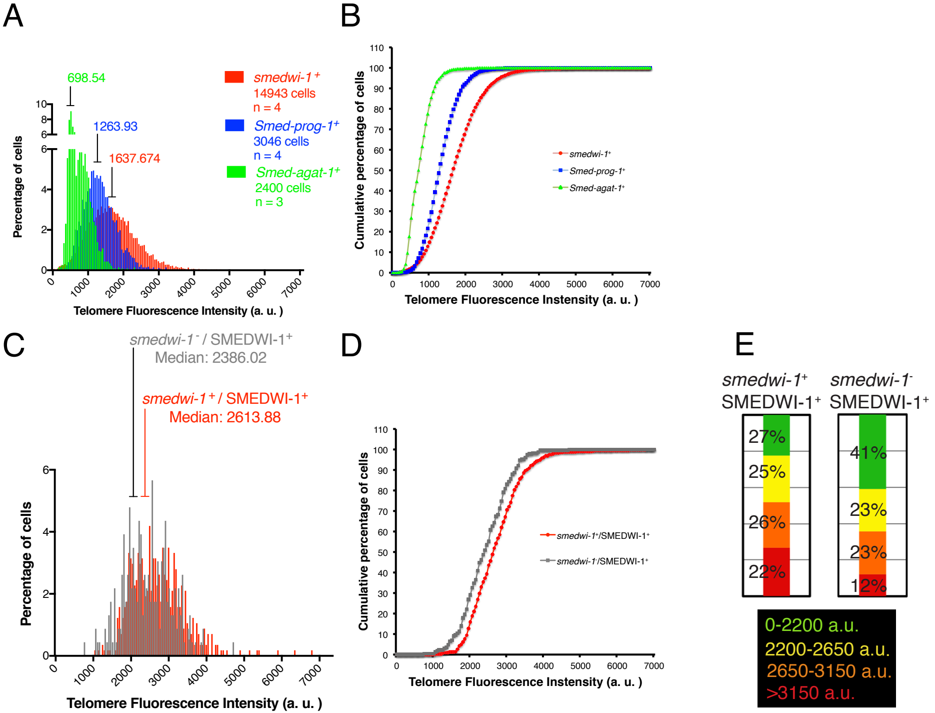
A
698.54
1263.93
1637.674
smedwi-1+
14943 cells
n = 4
Smed-prog-1+
3046 cells
n = 4
Smed-agat-1+
2400 cells
n = 3
Percentage of cells
Telomere Fluorescence Intensity (a. u.)
B
Cumulative percentage of cells
Telomere Fluorescence Instensity (a. u.)
smedwi-1+
Smed-prog-1+
Smed-agat-1+
C
smedwi-1- / SMEDWI-1+
Median: 2386.02
smedwi-1+ / SMEDWI-1+
Median: 2613.88
Percentage of cells
Telomere Fluorescence Intensity (a. u.)
D
Cumulative percentage of cells
Telomere Fluorescence Instensity (a. u.)
smedwi-1+/SMEDWI-1+
smedwi-1-/SMEDWI-1+
E
smedwi-1+ SMEDWI-1+
smedwi-1- SMEDWI-1+
27%
25%
26%
22%
41%
23%
23%
12%
0-2200 a.u.
2200-2650 a.u.
2650-3150 a.u.
>3150 a.u.

Figure S2. Different ways of representing telomere intensity data. Related to Figure 2. (A-B) Frequency histogram (A) and cumulative frequency graph (B) showing the data displayed in Fig. 2C. Stem cell distribution of telomere fluorescence intensity shows a higher heterogeneity (σ^2=492936.355) when compared to early and late postmitotic progeny (σ^2=198232.864 and σ^2=90484.769, respectively) (C-D) Frequency histogram (C) and cumulative frequency graph (D) showing the data displayed in Figure 2E. The *smedwi-1*$^+$/SMEDWI-1$^+$ stem cell population (σ^2=502727.219) shows higher heterogeneity when compared to their immediate progeny *smedwi-1*$^-$/SMEDWI-1$^+$ population (σ^2=399559.689) (E) The stacked bar graph represent the proportion of nuclei within a given category of intensity for the data displayed in Figure 2E. *smedwi-1*$^+$/SMEDWI-1$^+$ is chosen as the reference condition and set up to allocate in each range of intensity or category approximately one fourth of the total cells. *smedwi-1*$^-$/SMEDWI-1$^+$ shows a higher percentage of cells with short telomeres and a lower percentage of cells with long telomeres when compared to the *smedwi-1*$^+$/SMEDWI-1$^+$ population.

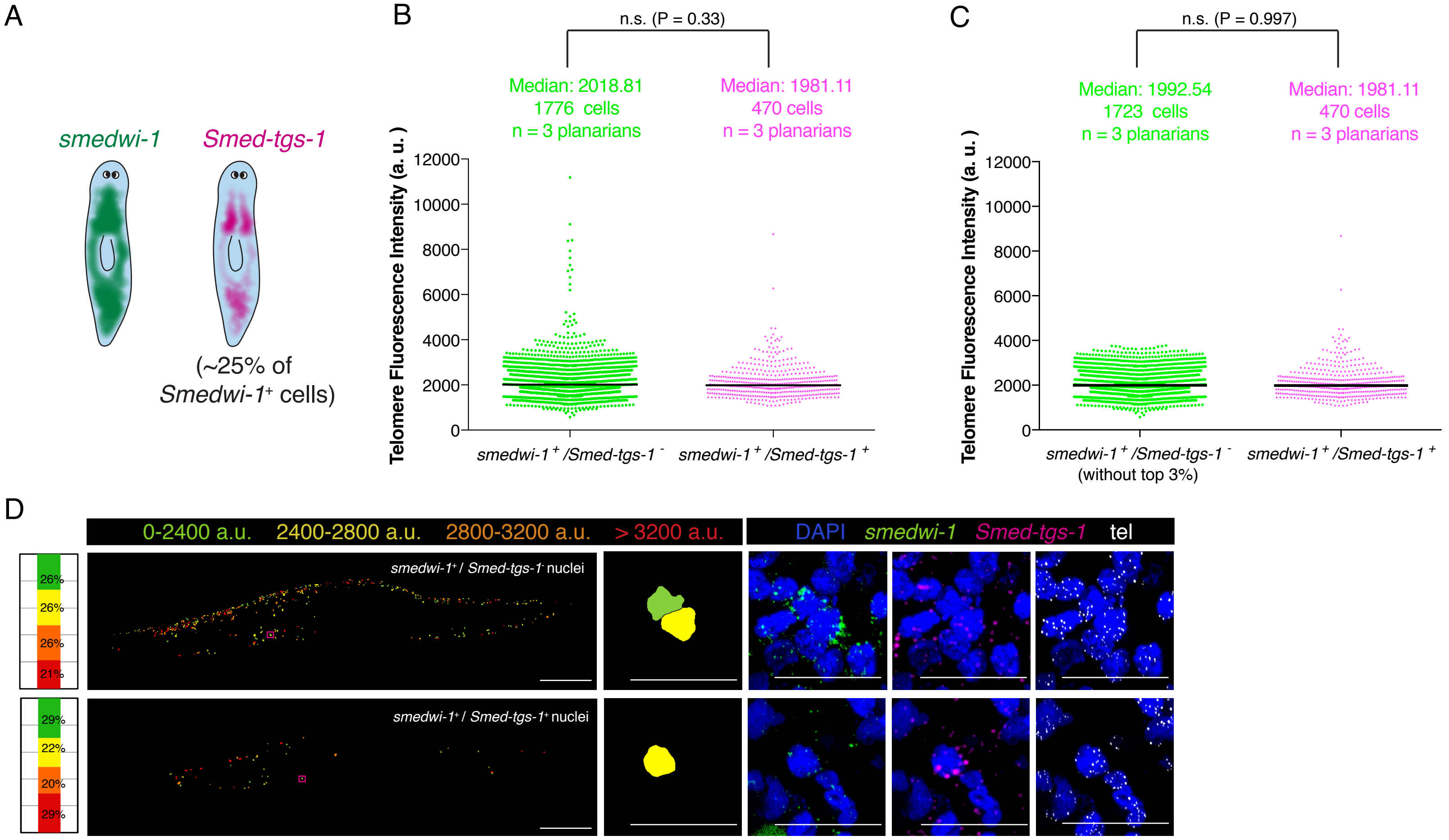

A
smedwi-1
Smed-tgs-1
(~25% of Smedwi-1⁺ cells)
B
n.s. (P = 0.33)
Median: 2018.81
1776 cells
n = 3 planarians
Median: 1981.11
470 cells
n = 3 planarians
Telomere Fluorescence Intensity (a. u.)
12000
10000
8000
6000
4000
2000
0
smedwi-1 + /Smed-tgs-1 -
smedwi-1 + /Smed-tgs-1 +
C
n.s. (P = 0.997)
Median: 1992.54
1723 cells
n = 3 planarians
Median: 1981.11
470 cells
n = 3 planarians
Telomere Fluorescence Intensity (a. u.)
12000
10000
8000
6000
4000
2000
0
smedwi-1 + /Smed-tgs-1 -
(without top 3%)
smedwi-1 + /Smed-tgs-1 +
D
0-2400 a.u.
2400-2800 a.u.
2800-3200 a.u.
> 3200 a.u.
DAPI
smedwi-1
Smed-tgs-1
tel
26%
26%
26%
21%
smedwi-1⁺ / Smed-tgs-1⁻ nuclei
29%
22%
20%
29%
smedwi-1⁺ / Smed-tgs-1⁺ nuclei

Figure S3. The *smedwi-1*$^+$ / *Smed-tgs-1*$^+$ population shows a similar telomere length distribution as the *smedwi-1*$^+$ /*Smed-tgs-1*$^-$ population. Related to Figure 4. (A) The schematic indicates the distribution of *smedwi-1*$^+$ cells (green) and *Smed-tgs-1*$^+$ cells (magenta) in a 7dS planarian. (B) Column scatter plot showing all the cells pooled from a total of 3 planarians for each condition. The median telomere intensity is not significantly different between *smedwi-1*$^+$/*Smed-tgs-1*$^-$ and *smedwi-1*$^+$/*Smed-tgs-1*$^+$ populations (two-tailed Mann-Whitney U test; P = 0.33). n indicates the number of planarians analyzed; a. u., arbitrary units; n.s., not significant. (C) Same column scatter plot as in B, this time comparing *smedwi-1*$^+$/*Smed-tgs-1*$^-$ cells which do not contain the top 3% of cells with the highest intensity values with the *smedwi-1*$^+$/*Smed-tgs-1*$^+$ population. There are not significant differences between both populations (two-tailed Mann-Whitney U test; P = 0.997). n indicates the number of planarians analyzed; a. u., arbitrary units; n.s., not significant. (D) Stacked bar graphs and telomere intensity maps from a representative tissue section of B broken down into two cell populations: *smedwi-1*$^+$/*Smed-tgs-1*$^-$ and *smedwi-1*$^+$/*Smed-tgs-1*$^+$ cells. The maps display the nuclei coloured according to their telomere fluorescence intensity (four categories of intensity). The stacked bar graphs represent the proportion of nuclei within a given category of intensity. *smedwi-1*$^+$/*Smed-tgs-1*$^-$ cells is chosen as the reference condition and set up to allocate in each range of intensity or category approximately one fourth of the total cells. Similar proportions are shown for both cell populations. Magenta squares indicate the area of magnification displayed next to the main images. a.u. indicates arbitrary units. Scale bars indicate 250 µm in the main images and 30 µm in the high magnification images.

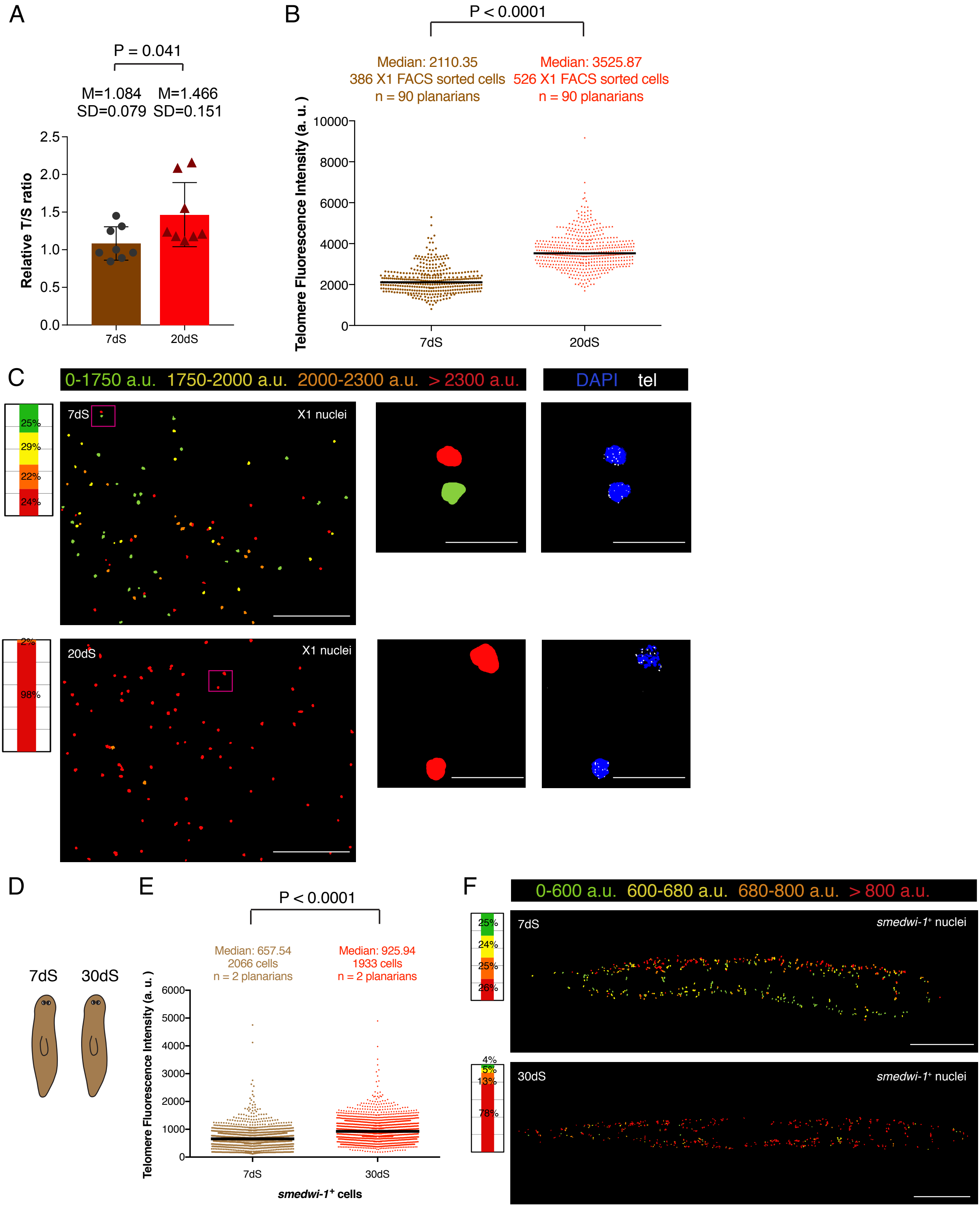
A
P = 0.041
M=1.084 M=1.466
SD=0.079 SD=0.151
Relative T/S ratio
2.5
2.0
1.5
1.0
0.5
0.0
7dS
20dS
B
P < 0.0001
Median: 2110.35
386 X1 FACS sorted cells
n = 90 planarians
Median: 3525.87
526 X1 FACS sorted cells
n = 90 planarians
Telomere Fluorescence Intensity (a. u.)
10000
8000
6000
4000
2000
0
7dS
20dS
C
0-1750 a.u. 1750-2000 a.u. 2000-2300 a.u. > 2300 a.u.
DAPI tel
25%
29%
22%
24%
7dS
X1 nuclei
2%
98%
20dS
X1 nuclei
D
7dS
30dS
E
P < 0.0001
Median: 657.54
2066 cells
n = 2 planarians
Median: 925.94
1933 cells
n = 2 planarians
Telomere Fluorescence Intensity (a. u.)
6000
5000
4000
3000
2000
1000
0
7dS
30dS
smedwi-1+ cells
F
0-600 a.u. 600-680 a.u. 680-800 a.u. > 800 a.u.
25%
24%
25%
26%
7dS
smedwi-1+ nuclei
4%
5%
13%
78%
30dS
smedwi-1+ nuclei

Figure S4. Starved planarians show a higher percentage of stem cells with long telomeres than 7 days starved planarians, independently of body size. Related to Figure 5. (A) Telomere quantitative PCR (qPCR) comparing 7dS and 20dS whole planarians. qPCR was performed on genomic DNA for 8 biological replicates (5 planarians per replicate) per condition. The qPCR determines the ratio of telomere (T) repeat copy number to a single-copy (S) gene (genomic DNA from dd_Smed_v6_2426_0_1) copy number (T/S ratio) compared with a reference DNA sample at 7dS. The graph shows that 20dS planarians have a higher T/S ratio than 7dS (two-tailed Student's t-test with equal sample variance, $P < 0.05$) and thus higher telomere length. M, mean; SD, standard deviation. (B) Column scatter plot showing X1 FACS sorted cells that come from planarians at 7dS and 20dS (90 planarians per condition were used for the FACS). The median telomere intensity is higher in stem cells from 20dS than from 7dS planarians (two-tailed Mann-Whitney U test; $P < 0.0001$); a. u. indicates arbitrary units. (C) Stacked bar graphs and telomere intensity maps from representative fields of X1 FACS sorted cells of 7dS and 20dS planarians displayed in B. The maps display the nuclei coloured according to their telomere fluorescence intensity (four categories of intensity). The stacked bar graphs represent the proportion of nuclei within a given category of intensity. X1 at 7dS is chosen as the reference condition and set up to allocate in each range of intensity or category approximately one fourth of the total cells. X1 at 20dS shows a higher percentage of stem cells with long telomeres and a lower percentage of stem cells with short telomeres than X1 at 7dS. Magenta squares indicate the area of magnification displayed next to the main images. a.u. indicates arbitrary units. Scale bars indicate 250 µm in the main images and 30 µm in the high magnification images. (D) The schematic displays the process of starvation of planarians that had the same size at 7dS and 30dS. (E) Column scatter plot showing all the cells pooled from a total of 2 planarians per condition. The median telomere intensity is higher in 30dS stem cells than in 7dS (two-tailed Mann-Whitney U test; $P < 0.0001$). n indicates the number of planarians analyzed. (F) Telomere intensity maps and stacked bar graphs for representative tissue sections from E. The intensity maps display the nuclei coloured according to their telomere fluorescence intensity (four categories of intensity). The stacked bar graphs represent the proportion of nuclei within a given category of intensity. 7dS is chosen as the reference condition and set up to allocate in each range of intensity or category approximately one fourth of the total cells. 30dS show a higher percentage of stem cells with high telomere intensity. a.u. indicates arbitrary units. Scale bars indicate 1 mm. Anterior is to the left and dorsal is up.

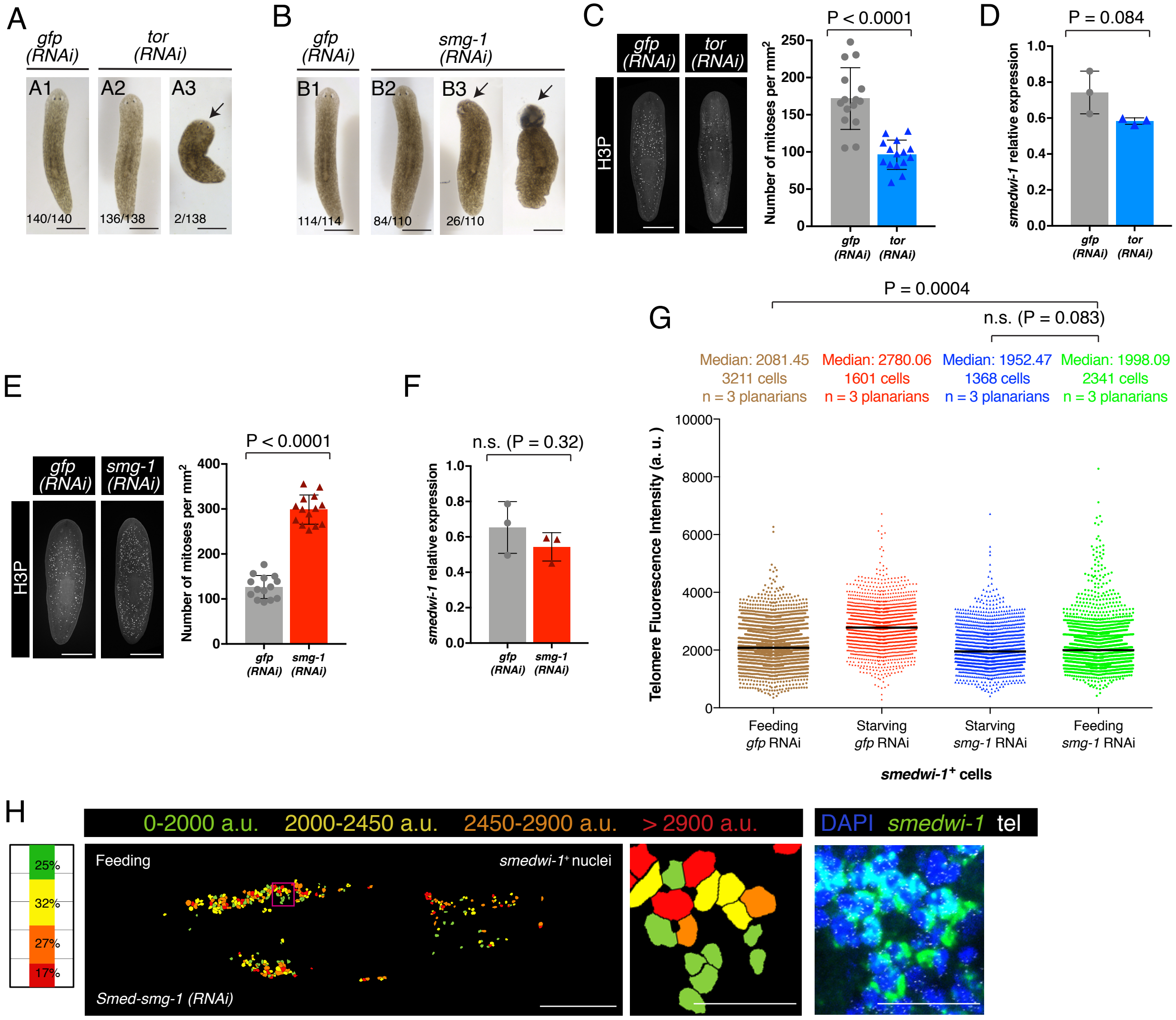

A
gfp (RNAi)
tor (RNAi)
A1
A2
A3
140/140
136/138
2/138
B
gfp (RNAi)
smg-1 (RNAi)
B1
B2
B3
114/114
84/110
26/110
C
gfp (RNAi)
tor (RNAi)
H3P
P < 0.0001
Number of mitoses per mm2
D
P = 0.084
smedwi-1 relative expression
E
gfp (RNAi)
smg-1 (RNAi)
H3P
P < 0.0001
Number of mitoses per mm2
F
n.s. (P = 0.32)
smedwi-1 relative expression
G
P = 0.0004
n.s. (P = 0.083)
Median: 2081.45
3211 cells
n = 3 planarians
Median: 2780.06
1601 cells
n = 3 planarians
Median: 1952.47
1368 cells
n = 3 planarians
Median: 1998.09
2341 cells
n = 3 planarians
Telomere Fluorescence Intensity (a. u.)
Feeding gfp RNAi
Starving gfp RNAi
Starving smg-1 RNAi
Feeding smg-1 RNAi
smedwi-1+ cells
H
0-2000 a.u.
2000-2450 a.u.
2450-2900 a.u.
> 2900 a.u.
25%
32%
27%
17%
Feeding
smedwi-1+ nuclei
Smed-smg-1 (RNAi)
DAPI
smedwi-1
tel

Figure S5. **Controls for *Smed-tor* and *Smed-smg-1* RNAi experiments. Related to Figure 6.** (A) Representative live images of *gfp(RNAi)* and *tor(RNAi)* planarians taken before fixation. Most of the *tor(RNAi)* planarians show no morphological phenotype at the time point chosen for fixation (day 42) (A2). Only A1 and A2 planarians were fixed for telomere length quantification. The arrow in A3 indicates head regression. Scale bars indicate 500 µm. (B) Representative live images of *gfp(RNAi)* and *smg-1(RNAi)* planarians taken before fixation. Most of the *smg-1(RNAi)* planarians show no morphological phenotype at the time point chosen for fixation (day 24) (B2). Only B1 and B2 planarians were fixed for telomere length quantification. The arrows in B3 indicate different degrees of tumour formation. Scale bars indicate 500 µm. (C) Representative images of *gfp(RNAi)* and *tor(RNAi)* planarians stained for anti-Histone H3 phosphorylated (anti-H3P) which labels mitotic stem cells at the time point chosen for fixation. The graph shows that *tor(RNAi)* planarians have lower number of mitoses than *gfp(RNAi)* animals (two-tailed Student's t-test with equal sample variance, $P < 0.0001$; $n = 15$ planarians per condition); the error bars indicate deviation from the mean; scale bars indicate 500 µm (D) Real time PCR shows that *smedwi-1* relative expression respect to the gene control is slightly lower in *tor(RNAi)* planarians than in the *gfp* (two-tailed Student's t-test with equal sample variance, $P = 0.084$; 3 biological replicates per condition) at the time point chosen for fixation; the error bars indicate deviation from the mean. (E) Representative images of *gfp(RNAi)* and *smg-1(RNAi)* planarians stained for anti-Histone H3 phosphorylated (anti-H3P) which labels mitotic stem cells at the time point chosen for fixation. The graph shows that *smg-1(RNAi)* planarians have higher number of mitoses than *gfp* animals (two-tailed Student's t-test with equal sample variance, $P < 0.0001$; $n = 14$ planarians per condition); the error bars indicate deviation from the mean; scale bars indicate 500 µm (F) Real time PCR shows that *smedwi-1* relative expression respect to the gene control is not significantly different in *smg-1* RNAi when compared to *gfp* controls (two-tailed Student's t-test with equal sample variance, $P = 0.32$; 3 biological replicates per condition) at the time point chosen for fixation; the error bars indicate deviation from the mean. (G) Column scatter plot shown in Figure 6F with an extra column that represents the "feeding *Smed-smg-1(RNAi)*" stem cell population. It shows all the cells pooled from a total of 3 planarians. The median telomere intensity is slightly lower in *Smed-smg-1(RNAi)* (feeding conditions) than in *gfp(RNAi)* (feeding conditions) stem cells (two-tailed Mann-Whitney U test; $P = 0.0004$). There are not significant differences between *Smed-smg-1(RNAi)* (feeding conditions) and *Smed-smg-1(RNAi)* (starving conditions) (two-tailed Mann-Whitney U test; $P = 0.083$). n indicates the number of planarians analyzed. (H) Stacked bar graph and telomere intensity map from a representative tissue section of *Smed-smg-1(RNAi)* (feeding conditions) planarians from G. The map displays the nuclei coloured according to their telomere fluorescence intensity (four categories of intensity). The stacked bar graphs represent the proportion of nuclei within a given category of intensity. *gfp(RNAi)* (feeding conditions) (Figure 6H) is chosen as the reference condition and set up to allocate in each range of intensity or category approximately one fourth of the total cells. Similar proportions are shown for *gfp(RNAi)* (feeding conditions) (Figure 6H) and *Smed-smg-1(RNAi)* (feeding conditions) cell populations. Magenta squares indicate the area of magnification displayed next to the main images. a.u. indicates arbitrary units. Scale bars indicate 200 µm in the main images and 30 µm in the high magnification images.

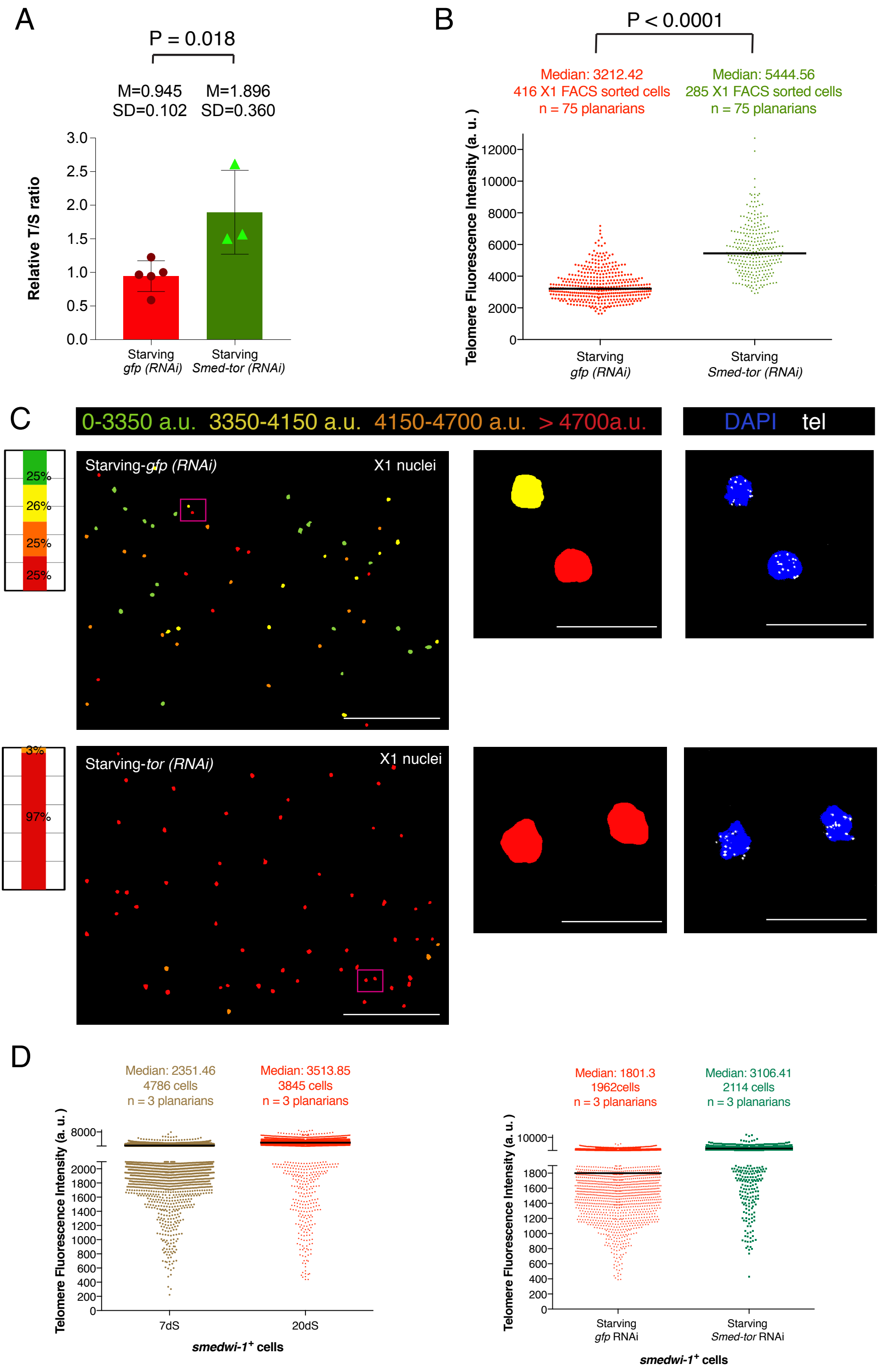

A
P = 0.018
M=0.945
SD=0.102
M=1.896
SD=0.360
Relative T/S ratio
3.0
2.5
2.0
1.5
1.0
0.5
0.0
Starving gfp (RNAi)
Starving Smed-tor (RNAi)
B
P < 0.0001
Median: 3212.42
416 X1 FACS sorted cells
n = 75 planarians
Median: 5444.56
285 X1 FACS sorted cells
n = 75 planarians
Telomere Fluorescence Intensity (a. u.)
12000
10000
8000
6000
4000
2000
0
Starving gfp (RNAi)
Starving Smed-tor (RNAi)
C
0-3350 a.u. 3350-4150 a.u. 4150-4700 a.u. > 4700a.u.
DAPI tel
25%
26%
25%
25%
Starving-gfp (RNAi)
X1 nuclei
3%
97%
Starving-tor (RNAi)
X1 nuclei
D
Median: 2351.46
4786 cells
n = 3 planarians
Median: 3513.85
3845 cells
n = 3 planarians
Telomere Fluorescence Intensity (a. u.)
8000
2000
1800
1600
1400
1200
1000
800
600
400
200
0
7dS
20dS
smedwi-1+ cells
Median: 1801.3
1962cells
n = 3 planarians
Median: 3106.41
2114 cells
n = 3 planarians
Telomere Fluorescence Intensity (a. u.)
10000
1800
1600
1400
1200
1000
800
600
400
200
0
Starving gfp RNAi
Starving Smed-tor RNAi
smedwi-1+ cells

Figure S6. *Smed-tor* RNAi further increases the effect of starvation on stem cell telomere length. Related to Figure 6. (A) Telomere quantitative PCR (qPCR) comparing *gfp(RNAi)* and *tor(RNAi)* whole planarians. qPCR was performed on genomic DNA for at least 3 biological replicates (5 planarians per replicate) per condition. The qPCR determines the ratio of telomere (T) repeat copy number to a single-copy (S) gene (genomic DNA from dd_Smed_v6_2426_0_1) copy number (T/S ratio) compared with a reference DNA sample *gfp* RNAi. The graph shows that *tor(RNAi)* planarians have a higher T/S ratio than *gfp(RNAi)* (two-tailed Student's t-test with equal sample variance, $P < 0.05$) and thus higher telomere length. M, mean; SD, standard deviation. (B) Column scatter plot showing X1 FACS sorted cells that come from *gfp* RNAi and *tor* RNAi (75 planarians per condition were used for the FACS). The median telomere intensity is higher in stem cells from *tor* RNAi than from *gfp* control planarians (two-tailed Mann-Whitney U test; $P < 0.0001$); a. u. indicates arbitrary units. (C) Stacked bar graphs and telomere intensity maps for representative fields of X1 FACS sorted cells from starving *gfp(RNAi)* and *tor(RNAi)* planarians. The maps display the nuclei coloured according to their telomere fluorescence intensity (four categories of intensity). The stacked bar graphs represent the proportion of nuclei within a given category of intensity. *gfp* RNAi is chosen as the reference condition and set up to allocate in each range of intensity or category approximately one fourth of the total cells. X1 cells from *tor(RNAi)* planarians show a higher percentage of stem cells with long telomeres and a lower percentage of stem cells with short telomeres than *gfp* control planarians. Magenta squares indicate the area of magnification displayed next to the main images. a.u. indicates arbitrary units. Scale bars indicate 250 µm in the main images and 30 µm in the high magnification images. (D) Column scatter plots showing the data from Figure 5C and Figure 6E respectively focusing on the stem cells with low telomere intensity.

Supplemental Experimental Procedures

Starvation experiments

Planarians were starved as stated in the text. In experiments displayed in Figure S4D-F, planarians at 7dS and 30dS had both the same area 4 mm^2 (5-5.5 mm length at 7dS and 5.5- 6mm length at 30dS). Graph paper placed under the Petri dish was used to pre-select animals of the same length and the final selection was done after measuring the areas with the Leica Application Suite (Leica) on photographs of live planarians taken under a stereomicrospe coupled with a Leica camera MC170 HD (Leica). For the rest of experiments planarians were 4-5.5 mm length at 7dS when starting the experiments and size selection was done on live animals by using graph paper.

RNAi experiments

At least 15 planarians per experiment were left to follow and corroborate the corresponding phenotypes. Planarians under feeding conditions were fed once a week, while planarians under starving conditions were left under starvation during the whole experiment. *tor* RNAi experiments could not be performed under feeding conditions since they are not able to maintain the pharynx and thus they cannot eat (Gonzalez-Estevez et al., 2012b).

Fixation, paraffin embedding and sectioning

Fixation of the planarians was performed by killing the animals at room temperature (RT) in 2% HCl diluted in 5/8 concentration of Holtfreter's standard saline solution for 5 minutes (min) followed by 2 washes of 5 min in Holtfreter 5/8 and fixation in 4% paraformaldehyde (16% Paraformaldehyde aqueous solution; Electron Microscopy Sciences) diluted in Holtfreter 5/8 for 4 hours at 4°C. After an overnight (ON) wash in Holtfreter 5/8, planarians were dehydrated through a series of ethanol washes diluted in RNase free miliQ water: 70% ethanol, 80% ethanol, 96% ethanol and 2 x 100% ethanol, 10 minutes each and a last step with 3 x xylene, 4 min each. The embedding of the samples was then performed either manually or with an Excelsior AS Tissue processor (Thermo Fisher Scientific). 7 μm sagittal sections were obtained for all the planarians and placed on Superfrost Plus slides (Menzel-Gläser; Thermo Fisher Scientific).

In situ hybridization on planarian sections

We generated templates with T7 promoters appended to the sense strand and hapten-labelled RNA probes were synthesized by using an *in vitro* labelling kit (Roche, Basel, Switzerland). The following oligos were used to obtain the templates:

smedwi-1F: 5'- ggccgcggAAGTGGTGGTATTCGAGAAGGA-3';

smedwi-1R: 5'- gccccggcCACGAATCGTAATCGGTTGTTCT-3';

Smed-nosF: 5'- ggccgcggCCTGAATCATTGAAGATGGCTA-3'

Smed-nosR: 5'- gccccggcCCAAGAGTGGATTGTGACATGCT-3'

Smed-prog1F: 5'-ggccgcggGTGATTGCGTTCGCGTATATT-3'

Smed-prog1R: 5'-gccccggcCATTTATCCAGCGCGTCATATTC-3'

Smed-Agat1F: 5'-ggccgcggGAAATGATTGAGTCCACCATGA-3'

Smed-Agat1R: 5'-gccccggcCTGCAATATCTGGATAAGGAGCA-3'

Smed-tgs-1F: 5'-ggccgcggAAAACGCAATCAAAGCAACTGA 3'

Smed-tgs-1R: 5'-gccccggcCAGTGAGAATGACGGATTCCTG 3'

Deparaffinization and rehydration of slide-mounted sections was performed either manually or with a Leica Autostainer XL (Leica Microsystems) through a series of ethanol washes diluted in RNase free miliQ water: 3 x xylene, 2 x 100% ethanol, 95% ethanol, 70% ethanol, 3 min each. In single fluorescent *in situ* hybridizations (FISH) permeabilization was performed in 10mM pH 6.0 citrate buffer by cooking the slides for 3 min in a pressure cooker. 1/100 anti-FITC-POD was used and tyramide signal amplification was performed by diluting the TSA plus reagent 1/50 in amplification buffer (TSA Plus Fluorescein or Cy5; Perkin Elmer) and incubating the slides for 10-20 min. For double FISH, the first POD was inactivated by incubating the slides in 2% H_2O_2 in PBST for one hour at RT followed by 6 washes of 10 min each in PBST and then 6 washes of 10 min each in Maleic buffer previous to apply the blocking solution. 1/100 anti-DIG-POD was used and tyramide signal amplification was performed by diluting the TSA plus reagent 1/50 in amplification buffer (TSA Plus Cy5; Perkin Elmer) and incubating the slides for 10-20 min. Nuclei counterstaining was performed by incubating the slides for 15 min in 5μg/ml DAPI.

Telomere Quantitative Fluorescent *in situ* hybridization (TelQ-FISH)

TelQ-FISH protocol on planarian paraffin sections started after either the FISH or the immunohistochemistry in the PBS washes that followed the DAPI staining. TelQ-FISH on FACS sorted cells started immediately after drying the slides. Slides (with paraffin sections or FACS sorted cells) were then fixed in 4% formalin in PBS for 2 min at RT, followed by 3 x PBS washes, 5 min each and a treatment with acidic pepsin for 10 min at 37°C. After washing 2 times for 5 min each in PBS, another 2 min fixation step with 4% formalin was performed and 2 x 5 min PBS washes. Then the slides went through a series of ethanol washes to dehydrate the tissue: 5 min ethanol 70%, 5 min ethanol 90% and 5 min ethanol 100%. Slides were air dried on the bench at RT for about 20 min or until totally dried. Then hybridization solution was added to each slide covered with a coverslip (10mM Tris pH 7.2, 8.56% Magnesium Buffer, 70% Deionized formamide, 0.25% Blocking reagent and 0.5 μg/ml Telomere CCCTAA PNA probe cy3-conjugated either from Applied Biosystems or from Panagene (TelC-Cy3 probe; since it has been shown that the planarian *S. mediterranea*, as other Platyhelminthes, have the same telomere repeat as vertebrates at their chromosome ends (Bombarova et al., 2009; Joffee, 1996) we used the vertebrate-specific telomere fluorescent peptide nucleic acid (PNA) probe). Tissue was denatured by placing the slides on a thermoblock at 80 °C for 3 min. Hybridization was then performed in a humid chamber in the dark for 2 hours at RT. Slides were washed 2 times for 15 min each with washing solution (70% formamide, 10mM Tris pH 7.2, 0.1% BSA) and then 3 times in PBST, 5 min each. The final step was an extra DAPI stain for 10 min. Slides were mounted in Vectashield (Vector Laboratories, Burlingame, California).

Telomere length image acquisition and quantification

Stacks (1 μm step size) for all the channels from equivalent tissue sections or FACS sorted cells were acquired either with a Leica TCS SP5 confocal microscope (Leica Microsystems; 16 bits images; 63x lens) or a Zeiss ApoTome.2 equipped with a Zeiss Axiocam 503 mono (Carl Zeiss, Jena; 14 bits images; 40x lens). Automatized high resolution z-stack imaging of several whole sections was possible by using the Matrix Screener Wizard from Leica at the Leica TCS SP5 confocal microscope or tile scan in the Zeiss ApoTome.2. Either the 561 nm DPSS laser was always maintained constant through all the slides from one same FISH or the exposition time in the case of the ApoTome. Cy3 samples with stronger signals from one same TelQ-FISH were used to set the intensity for the whole experimental scan to avoid having over-exposed signals. After automatically stitching all the tiles of each mosaic stack, a maximum projection (MP) for each channel was generated. In order to generate the binary masks containing nuclear areas based on the DAPI channel, the MP from the DAPI channel and the fluorescein/Cy5 channel needed first some processing by using a combination of Photoshop CS6 or CC (Abobe Systems Incorporated) and Fiji (Schindelin et al., 2012). Briefly, a Gaussian blur filter was applied to the DAPI MP to reduce the image noise or detail. Then the image was thresholded, transformed into a 1-bit binary image and watershed segmented to generate the DAPI mask. A Gaussian blur filter was also applied to the fluorescein/Cy5 image. A custom-made plugin (available upon request) for Fiji (Schindelin et al., 2012) was used to generate the binary mask for those nuclei positive for the fluorescein/Cy5 channels. In order to use the plugin, the fluorescein/Cy5 image was high percentage thresholded to obtain an image where the signal was kept at a minimum (few pixels per cell) and transformed into a 1-bit binary. The plugin generated a new mask (e.g., *smedwi-1* or *Smed-nanos* mask), with only those nuclei from the DAPI mask, which were positive for the specific markers. *smedwi-1*$^-$ masks were generated by substracting the *smedwi-1*$^+$ mask to the DAPI mask and using the arithmetic function "substract" in MetaMorph (version 6.3r6, Molecular Devices). By creating a multilayered image in Photoshop formed by the DAPI MP image, the DAPI mask, the telomere MP image, the fluorescein/Cy5 MP image and for instance *smedwi-1*$^+$, *Smed-nanos*$^+$ or *smedwi-1*$^-$ masks and changing the transparency of the layers as required, it was possible to improve the masks by manually fine segment those nuclei which were very close to each other (i. e. the brain cells and the pharynx cells), remove artifacts which were added by the plugin and to improve all the masks ensuring that all telomeres fell inside their nuclear area. The Cy3 image (telomeres) was only processed to remove general noise across the entire tissue section by applying the Detect Peaks feature from NIS-Elements software (Nikon Instruments) and left as originally taken at 16 or 14 bits ready for quantification.

Quantification was performed using the MetaMorph software (version 6.3r6, Molecular Devices). Briefly, 1-grey value was added to the original Cy3 image and then it was combined with a binarized DAPI mask through "logical and" arithmetic function. Then it was thresholded and Cy3 fluorescence intensity was measured as "average gray value" units (the total intensity per nucleus divided by the nucleus area) using the Integrated Morphometry Analysis module and shown as arbitrary units of fluorescence. Intensity values were exported to Excel for further analysis and to generate the cumulative frequency graphs and stacked bar charts. GraphPad Prism 7.0d was used to generate the column scatter plots and the frequency histograms. Arbitrary units of fluorescence cannot be compared between different scatter plots because differences in the protocols (e.g., single FISH versus FISH followed by immunohistochemistry), PNA aliquots and/or experimental design (e.g., wild type planarians versus GFP injected) lead to differences in the overall fluorescent intensity of the whole TelQ-FISH. All data obtained in the ApoTome (Figure 3 and Figure 4) was multiplied per 20 to bring the values into line with the ones obtained in the Confocal. The Configure Object Classifier module was used to classify and generate the telomere intensity maps colored according to their telomeric intensity.

Quantification of the number of telomeres per cell and intensity of the different telomeres in a cell

The number of telomeres per cell was calculated using Fiji (Schindelin et al., 2012). Each local maxima (telomere) was transformed into a single black pixel using the "find maxima" command. Then Regions of interest (ROI) were created based on nuclear DAPI staining and the "measure" command was then used to calculate "RawIntDen" (the sum of the values of the pixels in each ROI) which, divided by 255 (total black) results in the number of telomeres per nuclei. For measuring intensity per telomere an individual telomere ROI set for each nucleus was created and the sum of pixel-intensity (RawIntDen) for each ROI (telomere) measured using Fiji (Schindelin et al., 2012).

Whole-mount immunohistochemistry

Whole-mount immunohistochemistry was carried out as published elsewhere (Cebria and Newmark, 2005). An anti-Histone H3 phosphorylated at serine 10 (diluted 1/ 500; Santa Cruz, sc-8656-R) was used to detect mitotic stem cells. Whole planarian optical sections were obtained using a Zeiss AXIO Zoom.V16 (ApoTome.2) equipped with an Axiocam 506 camera (Carl Zeiss, Jena). Quantifications were done using the Object Counter 3D plugin from Fiji (Schindelin et al., 2012).

Real-time PCR

Real time PCR was performed as previously described (Gonzalez-Estevez et al., 2012a). The transcript with ID 5685 from Dresden transcriptome (PlanMine) (Rozanski et al., 2019) was used as internal control. 3 biological replicates (5 planarians per replicate) were used per sample. Each biological replicate was replicated three times. PCR reactions with performed using iTaq Universal SYBER® Green Supermix (BIO-RAD). Reactions were aliquoted using a QiAgility robot (Qiagen) and analyzed with a 7500 Real Time PCR System (Applied Biosystems).

Flurorescence-activated cell sorting (FACS)

Planarian dissociation and FACS were performed as described before (Hayashi et al., 2006), using BD FACSAria III. For TelQ-FISH, drops of 5 µl of sorted cells (subpopulation X1) at a concentration of 1000 cells/ml were placed on poly-lysine-coated slides and dried for 5 min at 37ºC. X1 subpopulation contains dividing stem cells since it disappears after lethal doses of gamma-irradiation (Hayashi et al., 2006).

Telomere quantitative PCR (telomere qPCR)

Genomic DNA extraction from 5 whole planarians per biological replicate was performed as previously described (Tan et al., 2012). Relative telomere length (TL) was measured by using an optimized version of the quantitative PCR method previously described (Cawthon, 2002). It determines the ratio of telomere (T) repeat copy number to a single-copy (S) gene copy number (called T/S ratio) in experimental samples as compared with a reference DNA sample. The single-copy gene used was the corresponding genomic sequence of transcript dd_Smed_v6_2426_0_1 (Smes_g4_19:2186986..2189312) in PlanMine (Rozanski et al., 2019). Telomere qPCRs and single-copy gene qPCRs were performed in separate wells using the following primers: TelF (5′-CGG TTT GTT TGG GTT TGG GTT TGG GTT TGG GTT TGG GTT-3′) and TelR (5′-GGC TTG CCT TAC CCT TAC CCT TAC CCT TAC CCT TAC CCT-3′) (O'Callaghan et al., 2008) used at a final concentration of 900 nM each; 2426F (5′-GTT GCT GGG CCA ATT AGG CG-3′) and 2426R (5′-CAA TTC GCT CTC TGA TCC GC-3′) used at final concentration of 300nM each. Each 10µl amplification reaction volume contained 1x Power SYBR Green PCR Master mix (Applied Biosystems) and 3 ng of genomic DNA samples. One of the replicates of 7dS (Figure S4A) and one of the replicates of *gfp (RNAi)* (Figure S6A) were used as reference DNA samples. Tubes containing 27, 9, 3, 1 and 0.333 ng of the reference DNA were included in each run to allow the quantification of the samples relative to the reference DNA by the standard curve method. Each biological replicate was run in triplicates. At least 3 biological replicates per condition were analysed. qPCRs were carried out on The StepOne Plus Real-Time PCR System (Applied Biosystems). PCR cycling conditions for the telomere amplification were 95°C for 10 min, followed by 40 cycles of 95°C for 15 sec, 58°C for 30 sec and 72 °C for 30 sec, and for the 2426 gene the cycling conditions were 95°C for 10 min, followed by 40 cycles of 95°C for 15 sec and 65°C for 1 min. Melting curve analysis was carried out at the end of each PCR experiment.

References

Bombarova, M., Vitkova, M., Spakulova, M., and Koubkova, B. (2009). Telomere analysis of platyhelminths and acanthocephalans by FISH and Southern hybridization. Genome *52*, 897-903.

Cawthon, R.M. (2002). Telomere measurement by quantitative PCR. Nucleic Acids Res *30*, e47.

Cebria, F., and Newmark, P.A. (2005). Planarian homologs of netrin and netrin receptor are required for proper regeneration of the central nervous system and the maintenance of nervous system architecture. Development *132*, 3691-3703.

Gonzalez-Estevez, C., Felix, D.A., Rodriguez-Esteban, G., and Aboobaker, A.A. (2012a). Decreased neoblast progeny and increased cell death during starvation-induced planarian degrowth. Int J Dev Biol *56*, 83-91.

Gonzalez-Estevez, C., Felix, D.A., Smith, M.D., Paps, J., Morley, S.J., James, V., Sharp, T.V., and Aboobaker, A.A. (2012b). SMG-1 and mTORC1 act antagonistically to regulate response to injury and growth in planarians. PLoS Genet *8*, e1002619.

Hayashi, T., Asami, M., Higuchi, S., Shibata, N., and Agata, K. (2006). Isolation of planarian X-ray-sensitive stem cells by fluorescence-activated cell sorting. Dev Growth Differ *48*, 371-380.

Joffee, B.S.I., Macgregor HC (1996). Ends of Chromosomes in Polycelis tenuis (Platyhelminthes) have telomere repeat TTAGGG. Chormosome Research *4*, 323-324.

O'Callaghan, N., Dhillon, V., Thomas, P., and Fenech, M. (2008). A quantitative real-time PCR method for absolute telomere length. BioTechniques *44*, 807-809.

Rozanski, A., Moon, H., Brandl, H., Martin-Duran, J.M., Grohme, M.A., Huttner, K., Bartscherer, K., Henry, I., and Rink, J.C. (2019). PlanMine 3.0-improvements to a mineable resource of flatworm biology and biodiversity. Nucleic Acids Res *47*, D812-D820.

Schindelin, J., Arganda-Carreras, I., Frise, E., Kaynig, V., Longair, M., Pietzsch, T., Preibisch, S., Rueden, C., Saalfeld, S., Schmid, B.*, et al.* (2012). Fiji: an open-source platform for biological-image analysis. Nat Methods *9*, 676-682.

Tan, T.C., Rahman, R., Jaber-Hijazi, F., Felix, D.A., Chen, C., Louis, E.J., and Aboobaker, A. (2012). Telomere maintenance and telomerase activity are differentially regulated in asexual and sexual worms. Proc Natl Acad Sci U S A *109*, 4209-4214.

Video S1. Tissue section stained for telomeres.
The video shows a zoom into a tissue section stained for telomeres with increased exposure in order to be able to observe all the telomeres from all the cells in all the planarian tissues.

Video S2. The highest telomere intensity in a given tissue section can be easily observed.
The video shows a zoom into the dorsal part of a tissue section. The brightest telomeres can be easily seen (arrows).
